# Supplementary material for: Multiple glutathione-S-transferases detoxify diverse glucosinolate-based defenses of Brassicales plants in a generalist lepidopteran herbivore (Spodoptera littoralis)
Source: Commun Biol. 2025 Jun 17;8:931. doi: 10.1038/s42003-025-08346-8 (PMC12174363; doi:10.1038/s42003-025-08346-8)
Supplement: Supplementary file 2 — Supplementary information [file 42003_2025_8346_MOESM2_ESM.pdf]

Supplementary Information

Multiple glutathione-*S*-transferases detoxify diverse glucosinolate-based defenses of Brassicales plants in a generalist lepidopteran herbivore (*Spodoptera littoralis*)

Ruo Sun, Samantha Römhild, Yoko Nakamura, Michael Reichelt, Katrin Luck, Duc Tam Mai, Beate Rothe, Jonathan Gershenzon, Daniel Giddings Vassão

Supplementary Note 1 NMR analyses of isothiocyanate (ITC) conjugates.

Supplementary Note 2 Preparation of artificial diet for *Spodoptera littoralis*

Supplementary Table 1. Glucosinolate (GSL) content in *Arabidopsis thaliana* wild-type and mutant plants

Supplementary Table 2. Specific activities of recombinant *Spodoptera littoralis* glutathione-*S*-transferase (GST) proteins toward isothiocyanates (ITCs)

Supplementary Table 3. HPLC-MS/MS parameters used for the multiple reaction monitoring (MRM) analyses

Supplementary Table 4. Chemical standards and compound information

Supplementary Table 5. Basic information of *GST* genes and GST proteins

Supplementary Figure 1 Inhibition of *Spodoptera littoralis* larval growth by isothiocyanate (ITC) and insecticides.

Supplementary Figure 2 Quantitative conversion of 4MSOB ITC into its conjugates during *S. littoralis* feeding.

Supplementary Figure 3 Metabolism of ITCs by *S. littoralis* via the mercapturic acid pathway and lysine conjugation.

Supplementary Figure 4 NMR spectra

Supplementary Figure 5 The chromatographic analysis of standard isothiocyanate (ITC) conjugates via UHPLC-qTOFMS.

Supplementary Figure 6 The detectable ITCs and ITC conjugates found in *S. littoralis* fed on glucosinolate (GSL)-containing *Arabidopsis thaliana* plants.

Supplementary Figure 7 Enzymatic activity and phylogenetic analysis of glutathione-*S*-transferases (GSTs) catalyzing the conjugation of GSH with ITCs from this study and the literature.

Supplementary Figure 8 Inducibility of *GST* genes in *S. littoralis* larval midgut caused by feeding on artificial diets containing ITCs.

Supplementary Figure 9 SDS-PAGE analysis of purified *S. littoralis* His-tagged GST proteins expressed in the *Escherichia coli* BL21 (DE3) strain.

Supplementary Figure 10 The original electrophoretic gels

Supplementary Note 1. NMR analyses of isothiocyanate conjugates

Characterization of of ITC conjugates

4MTB ITC-Cyclic-Cys: ^{1}H-NMR (500 MHz, MeOH-d_3, 238 K): δ ppm: 4.64 (*dd, J*= 8.3, 3.4 Hz, 1H), 3.89 (*dd, J*= 11.1, 8.8 Hz, 1H), 3.69 (*dd, J*= 11.1, 3.4 Hz, 1H), 3.50-3.36 (*m*, 2H), 2.54 (*t*, *J*= 7.1 Hz, 2H), 2.08 (*s*, 3H), 1.76 (*m*, 2H), 1.68 (*m*, 2H). ^{13}C-NMR (126 MHz, MeOH-d_3, 238 K): δ ppm: 175.2, 171.6, 65.2, 46.0, 35.9, 34.0, 27.9, 26.8, 14.9.

2PE ITC-Cyclic-Cys: ^{1}H-NMR (500 MHz, MeOH-d_3, 238 K): δ ppm: 7.33 (*m,* 2H), 7.31 (*m,* 2H), 7.24 (*m,* 1H), 4.63 (*dd, J*= 8.6, 3.1 Hz, 1H), 3.87 (*dd, J*= 11.1, 8.6 Hz, 1H), 3.69 (*dd, J*= 11.1, 3.1 Hz, 1H), 3.71 (*m*, 1H), 3.62 (*m*, 1H), 3.05-2.88 (*m*, 2H). ^{13}C-NMR (126 MHz, MeOH-d_3, 238 K): δ ppm: 175.2, 171.8, 139.1, 129.9, 129.6, 127.8, 65.2, 47.6, 35.8, 34.9.

4MTB ITC-CysGly: ^{1}H-NMR (500 MHz, D_2O, 298 K): δ ppm: 4.26 (*dd, J*= 6.5, 6.1 Hz, 1H), 3.84 (*dd, J*= 15.1, 6.1 Hz, 1H), 3.80 (*d, J*= 17.1 Hz, 1H), 3.69 (*dd, J*= 15.1, 6.5 Hz, 1H), 3.63 (*dd*, *J*= 6.9, 6.9 Hz, 2H), 3.59 (*d, J*= 17.1 Hz, 1H), 2.48 (*t, J*= 7.3 Hz, 2H), 2.00 (*s*, 3H), 1.67 (*m*, 2H), 1.57 (*m*, 2H). ^{13}C-NMR (126 MHz, D_2O, 298 K): δ ppm: 194.9, 175.9, 167.7, 52.8, 47.1, 43.4, 34.3, 32.7, 26.2, 25.5, 14.0.

Benzyl ITC-CysGly: ^{1}H-NMR (500 MHz, D_2O, 298 K): δ ppm: 7.33-7.23 (*m*, 5H), 4.79 (*brs*, 2H), 4.21 (*dd, J*= 6.4, 6.4 Hz, 1H), 3.81 (*dd, J*= 15.2, 6.4 Hz, 1H), 3.75 (*d, J*= 17.2 Hz, 1H), 3.67 (*dd, J*= 15.2, 6.4 Hz, 1H), 3.51 (*d, J*= 17.2 Hz, 1H). ^{13}C-NMR (126 MHz, D_2O, 298 K): δ ppm: 196.0, 176.0, 168.1, 136.3, 128.8, 127.8, 127.7, 52.9, 50.6, 43.3, 34.7.

4MTB ITC-Cys: ^{1}H-NMR (500 MHz, D_2O, 298 K): δ ppm: 4.05 (*dd, J*= 6.8, 4.4 Hz, 1H), 4.00 (*dd, J*= 15.0, 4.4 Hz, 1H), 3.71-3.60 (*m*, 2H), 3.61 (*m*, 1H), 2.50 (*t, J*= 7.1 Hz, 2H), 2.02 (*s*, 3H), 1.69 (*m*, 2H), 1.59 (*m*, 2H). ^{13}C-NMR (126 MHz, D_2O, 298 K): δ ppm: 195.9, 172.6, 54.4, 47.2, 34.7, 32.7, 26.3, 25.6, 14.0.

Benzyl ITC-Cys: ^{1}H-NMR (500 MHz, MeOH-d_3, 298 K): δ ppm: 7.35-7.23 (*m*, 5H), 4.89 (*m*, 2H), 4.08 (*dd, J*= 14.8, 4.3 Hz, 1H), 3.93 (*dd, J*= 7.0, 4.3 Hz, 1H), 3.65 (*dd, J*= 14.8, 7.0 Hz, 1H). ^{13}C-NMR (126 MHz, MeOH-d_3, 298 K): δ ppm: 197.9, 172.3, 138.2, 129.4, 129.1, 128.5, 56.0, 51.8, 36.6.

Butyl ITC-Cys: ^{1}H-NMR (500 MHz, D_2O, 298 K): δ ppm: 4.04 (*dd, J*= 6.8, 4.2 Hz, 1H), 4.00 (*dd, J*= 15.0, 4.2 Hz, 1H), 3.61 (*m*, 2H), 3.60 (*dd*, *J*= 15.0, 6.8 Hz, 1H), 1.56 (*m*, 2H), 1.28 (*m*, 2H), 0.83 (*t, J*= 7.4 Hz, 3H). ^{13}C-NMR (126 MHz, D_2O, 298 K): δ ppm: 195.5, 172.3, 54.4, 47.6, 34.7, 29.2, 19.5, 12.9.

Benzyl ITC-Lys: ^{1}H-NMR (500 MHz, MeOH-d_3, 298 K): δ ppm: 7.41-7.20 (*m*, 5H), 4.92 (*m*, 2H), 3.44 (*dd, J*= 6.7, 5.3 Hz, 1H), 2.82 (*dd, J*= 7.3, 7.3 Hz, 1H), 1.88-1.68 (*m*, 2H), 1.66-1.51 (*m*, 2H), 1.44 (*m*, 2H). ^{13}C-NMR (126 MHz, MeOH-d_3, 298 K): δ ppm: 185.0, 176.5, 137.7, 129.4, 128.5, 128.4, 56.5, 44.9, 41.3, 33.2, 29.9, 23.6.

NMR spectra are listed in Supplementary Figure 4

Supplementary Note 2

Preparation of artificial diet for *Spodoptera littoralis*

Ingredients

Component	Source / Catalog Number
Fine bean flour	Cellar der Boländer (fine)
Coarse (groat) bean flour	Cellar der Boländer (grob)
Ascorbic acid (Vitamin C)	Fluka 54660; Roth 6288.2
Ethyl-4-benzoic acid	Aldrich 19128-0
α-Tocopherol (Vitamin E)	Fluka 95240
Sunflower oil (Mazola-Keimöl)	Commercial source (e.g., supermarket)
Formaldehyde (37%)	Roth 4979.1
Agar-Agar	Roth 2266.2 / Kobe I Agar: Roth 5210.2

Preparatory Steps

1. Prepare an oil mix by dissolving 5 g of α-tocopherol in 70 mL of sunflower oil. Store at 4 °C until use.
2. Freshly prepare 3.7% formaldehyde solution by diluting 0.4 mL of 37% formaldehyde in 3.6 mL of distilled water.
3. Weigh:
   - 9 g ascorbic acid
   - 5 g ethyl-4-benzoic acid
   - Approximately 10 mL of the tocopherol–oil mixture (corresponds to ~9 g)
   - 350 g fine bean flour
   - 150 g coarse (groat) bean flour

Diet Preparation Protocol

1. Combine the fine and coarse bean flours in a single beaker and mix thoroughly.

2. In a 3 L low-form beaker, dissolve 75 g agar in 1000 mL of prewarmed distilled water. Heat the mixture in a microwave for 5–8 minutes, stirring intermittently, until the agar reaches a full boil. Ensure boiling, as underheated agar results in a soft, suboptimal diet texture.
3. Add an additional 600 mL of prewarmed water to the hot agar and mix well.
4. Gradually incorporate the flour blend into the agar solution while mixing with a handheld electric mixer until homogenous.
5. Add the ascorbic acid, ethyl-4-benzoic acid, freshly prepared formaldehyde solution, and tocopherol–oil mixture. Continue mixing until all components are fully dissolved and evenly distributed.
6. Pour the finished diet into 3–4 storage boxes and allow it to cool.

Supplementary Table 1 Glucosinolate (GSL) content in *Arabidopsis thaliana* wild-type and mutant plants.

GSL	Col-0 (wild-type)		*myb28myb29*		*myb28myb29* × *cyp79b2cyp79b3*		*CYP79A2*		Significant difference
	GSL content (mean ± s.e.), μmol · g^{-1} dry weight								Tukey HSD tests in conjunction with one-way ANOVA
Total	20.25±1.60	c *	5.38±0.30	b	0.00±0.00	a	39.23±0.68	d	$F_{3,16}$= 395.5; $P\leq$ 0.001
I3M GSL	2.74±0.13	c	4.69±0.29	d	0.00±0.00	a	1.49±0.06	b	$F_{3,16}$= 148.6; $P\leq$ 0.001
4MOI3M GSL	0.47±0.05	c	0.70±0.04	d	0.00±0.00	a	0.25±0.01	b	$F_{3,16}$= 98.26; $P\leq$ 0.001
3MSOP GSL	1.96±0.17	b	0.00±0.00	a	0.00±0.00	a	2.18±0.03	b	$F_{3,16}$= 192.9; $P\leq$ 0.001
4MSOB GSL	14.15±1.19	b	0.00±0.00	a	0.00±0.00	a	14.85±0.46	b	$F_{3,16}$= 172.0; $P\leq$ 0.001
5MSOP GSL	0.36±0.03	b	0.00±0.00	a	0.00±0.00	a	0.34±0.01	b	$F_{3,16}$= 217.8; $P\leq$ 0.001
8MSOO GSL	0.57±0.10	b	0.00±0.00	a	0.00±0.00	a	0.99±0.17	c	$F_{3,16}$= 23.46; $P\leq$ 0.001
Benzyl GSL	0.00±0.00	a	0.00±0.00	a	0.00±0.00	a	19.14±0.21	b	$F_{3,16}$= 8097; $P\leq$ 0.002
Others**	2.14±0.44	b	0.15±0.02	a	0.00±0.00	a	1.75±0.33	b	$F_{3,16}$= 54.36; $P\leq$ 0.001

*: Lowercase letters in the table denote significant differences.

**: The other GSLs include 4OHI3M GSL, 7MSOH GSL, 4MTB GSL, and 1MOI3M GSL

Supplementary Table 2 Specific activities of recombinant *Spodoptera littoralis* glutathione-*S*-transferase (GST) proteins toward isothiocyanates (ITCs).

GST enzyme	Isothiocyanate Substrate (R-group)							
	allyl ITC	butyl ITC	sec-butyl ITC	iso-butyl ITC	3MSOP ITC	4MSOB ITC	benzyl ITC	2PE ITC
	Specific activity (mean ± s.e.), μmol · mg^{-1} · min^{-1}							
GSTD1	0.70 ± 0.12	0.77 ± 0.04	0.05 ± 0.05	0.39 ± 0.01	-0.18 ± 0.08	0.20 ± 0.04	0.51 ± 0.02	0.48 ± 0.00
GSTD2	0.52 ± 0.13	0.15 ± 0.02	0.03 ± 0.05	0.43 ± 0.03	-0.14 ± 0.01	-0.02 ± 0.01	1.04 ± 0.08	0.40 ± 0.02
GSTD3	1.96 ± 0.33	0.78 ± 0.29	0.47 ± 0.06	0.92 ± 0.06	1.05 ± 0.25	1.50 ± 0.13	7.44 ± 0.29	1.81 ± 0.02
GSTE1	1.19 ± 0.02	0.27 ± 0.02	0.10 ± 0.01	0.23 ± 0.02	1.25 ± 0.08	0.94 ± 0.13	0.38 ± 0.03	0.62 ± 0.04
GSTE2	3.41 ± 0.49	2.67 ± 0.37	1.52 ± 0.08	1.98 ± 0.17	0.79 ± 0.08	0.24 ± 0.10	4.59 ± 0.35	1.88 ± 0.13
GSTE3	1.62 ± 0.09	1.07 ± 0.03	1.00 ± 0.04	0.84 ± 0.03	2.33 ± 0.03	0.80 ± 0.05	2.13 ± 0.12	1.08 ± 0.02
GSTE4	0.34 ± 0.12	-0.17 ± 0.03	0.21 ± 0.15	0.19 ± 0.05	0.28 ± 0.10	0.05 ± 0.06	0.44 ± 0.03	0.25 ± 0.02
GSTE5	1.16 ± 0.05	0.43 ± 0.07	0.20 ± 0.05	0.30 ± 0.04	0.14 ± 0.06	0.54 ± 0.08	1.42 ± 0.10	0.90 ± 0.09
GSTE6	-0.09 ± 0.10	-0.09 ± 0.06	0.01 ± 0.02	0.02 ± 0.07	0.25 ± 0.03	-0.13 ± 0.03	0.01 ± 0.03	-0.27 ± 0.10
GSTE7	0.07 ± 0.07	0.41 ± 0.04	0.01 ± 0.16	-0.08 ± 0.03	-0.33 ± 0.10	-0.03 ± 0.03	0.30 ± 0.05	-0.24 ± 0.13
GSTE8	5.36 ± 0.10	2.55 ± 0.15	3.43 ± 0.18	2.30 ± 0.03	0.78 ± 0.09	0.52 ± 0.27	2.73 ± 0.06	1.28 ± 0.05
GSTE9	55.08 ± 1.20	43.94 ± 1.03	26.77 ± 2.16	21.01 ± 1.45	3.03 ± 0.10	1.99 ± 0.18	26.41 ± 0.68	11.20 ± 0.28
GSTE10	4.34 ± 0.10	1.98 ± 0.05	2.67 ± 0.04	1.49 ± 0.06	6.11 ± 0.00	1.68 ± 0.07	1.53 ± 0.04	1.12 ± 0.04
GSTE11	0.15 ± 0.04	0.01 ± 0.03	0.12 ± 0.10	-0.14 ± 0.03	-0.36 ± 0.08	-0.07 ± 0.02	0.37 ± 0.04	0.04 ± 0.06
GSTE12	2.77 ± 0.15	2.36 ± 0.24	1.36 ± 0.06	1.38 ± 0.01	1.49 ± 0.07	0.83 ± 0.02	3.54 ± 0.12	1.80 ± 0.16
GSTE13	9.91 ± 0.37	9.53 ± 0.28	9.02 ± 1.02	8.62 ± 0.27	3.41 ± 0.09	3.80 ± 0.27	10.85 ± 0.94	8.44 ± 0.32
GSTE14	26.56 ± 0.31	5.82 ± 0.23	7.62 ± 0.76	4.66 ± 0.23	0.74 ± 0.07	2.50 ± 0.00	17.45 ± 0.55	16.48 ± 0.73
GSTE15	1.28 ± 0.07	0.83 ± 0.09	0.22 ± 0.14	0.24 ± 0.05	0.23 ± 0.03	0.33 ± 0.05	0.64 ± 0.05	0.16 ± 0.08
GSTE16	1.93 ± 0.12	1.94 ± 0.02	2.94 ± 0.10	2.24 ± 0.09	1.33 ± 0.08	1.35 ± 0.01	3.78 ± 0.03	1.53 ± 0.03
GSTE17	5.44 ± 0.24	2.93 ± 0.18	2.69 ± 0.03	2.39 ± 0.02	0.06 ± 0.11	1.04 ± 0.02	11.27 ± 0.37	5.16 ± 0.01
GSTO1	0.13 ± 0.02	-0.07 ± 0.13	-0.04 ± 0.04	-0.01 ± 0.08	-0.06 ± 0.01	-0.06 ± 0.09	-0.18 ± 0.10	-0.06 ± 0.05
GSTO2	-0.06 ± 0.11	-0.12 ± 0.06	-0.22 ± 0.04	-0.12 ± 0.06	-0.06 ± 0.02	-0.22 ± 0.06	-0.08 ± 0.13	-0.05 ± 0.03
GSTO3	0.18 ± 0.21	-0.06 ± 0.02	0.01 ± 0.01	0.01 ± 0.02	0.34 ± 0.14	0.12 ± 0.04	-0.03 ± 0.00	-0.08 ± 0.03
GSTS1	-0.03 ± 0.04	-0.13 ± 0.05	-0.04 ± 0.08	-0.14 ± 0.03	0.02 ± 0.04	-0.06 ± 0.02	0.61 ± 0.02	0.29 ± 0.02
GSTS2	0.95 ± 0.03	1.04 ± 0.02	0.88 ± 0.26	1.15 ± 0.07	1.81 ± 0.09	1.82 ± 0.17	8.19 ± 0.62	1.40 ± 0.01
GSTS3	0.66 ± 0.11	1.00 ± 0.22	0.29 ± 0.06	0.78 ± 0.17	2.02 ± 0.03	0.54 ± 0.03	4.65 ± 0.07	1.60 ± 0.02
GSTS4	0.23 ± 0.01	0.31 ± 0.02	0.11 ± 0.02	0.14 ± 0.05	0.06 ± 0.08	0.45 ± 0.02	1.27 ± 0.04	0.70 ± 0.01
GSTS5	7.77 ± 0.25	2.64 ± 0.11	1.38 ± 0.11	2.06 ± 0.06	3.99 ± 0.20	2.07 ± 0.08	20.42 ± 1.04	2.26 ± 0.03
GSTS6	2.74 ± 0.38	1.50 ± 0.24	0.52 ± 0.07	0.70 ± 0.08	0.87 ± 0.05	0.05 ± 0.08	21.67 ± 0.29	3.45 ± 0.14
GSTT1	0.02 ± 0.08	0.14 ± 0.02	0.00 ± 0.02	-0.03 ± 0.02	0.04 ± 0.03	-0.15 ± 0.05	3.47 ± 0.24	0.19 ± 0.04
GSTU1	0.30 ± 0.08	-0.07 ± 0.06	-0.02 ± 0.04	0.05 ± 0.01	0.19 ± 0.02	0.08 ± 0.18	-0.03 ± 0.01	-0.02 ± 0.02
GSTU2	3.92 ± 0.29	1.74 ± 0.15	0.45 ± 0.08	1.67 ± 0.09	1.93 ± 0.13	0.37 ± 0.06	3.83 ± 0.21	2.35 ± 0.08
GSTZ1	-0.18 ± 0.03	-0.03 ± 0.05	-0.11 ± 0.05	0.13 ± 0.00	-0.36 ± 0.03	0.17 ± 0.02	0.18 ± 0.04	-0.08 ± 0.08
GSTZ2	-0.62 ± 0.07	0.01 ± 0.04	-0.02 ± 0.02	-0.09 ± 0.05	-0.09 ± 0.03	-0.01 ± 0.01	0.15 ± 0.05	0.03 ± 0.08

Supplementary Table 3 HPLC-MS/MS parameters used for the multiple reaction monitoring (MRM) analyses.

Metabolites	mass spectrometer	Ionization mode	Q1 m/z	Q3 MRM fragment	Retention time (min)	Dp (V)	EP (V)	CE (V)	CXP (V)
4MSOB ITC	API5000	Positive mode	178.11	114	2.5	51	8	13	4
4MSOB ITC-GSH			485.11	179.1	2	76	8	29	6
4MSOB ITC-Cys-Gly			356.07	136.1	1.9	46	8	15	4
4MSOB ITC-Cys			299.06	136.1	1.7	51	8	15	4
4MSOB ITC-cyclic-Cys			265.11	201	1.1	51	8	25	4
GSH	API5000	Negative mode	306.2	143	6.2	-60	-9	-28	-2
GSSG			611.2	306	9.5	-60	-7	-34	-6
Gly-FMOC	API5000	Negative mode	296	74	4.3	-60	-3	-10	-4
labGly-FMOC			299	77	4.3	-60	-3	-10	-4
Cys-FMOC-I			342	146	5.8	-60	-8	-12	-9
labCys-FMOC-I			346	150	5.8	-60	-8	-12	-9
(cys)2(fmoc)2-II			683.3	152	5.3	-60	-8	-34	-15
lab(cys)2(FMOC)2-II			691.3	156	5.3	-60	-8	-34	-15
labPhe-FMOC			396	174	5	-60	-3	-10	-4

Metabolites	mass spectrometer	Ionization mode	Q1 m/z	Q3 MRM fragment	Retention time (min)	Dp (V)	EP (V)	CE (V)	CXP (V)
Ala	API6500	Positive mode	90.1	44.1	0.4	20	5.5	17	4
Ser			106	60.1	0.4	20	4.5	15	4
Pro			116.1	70	0.6	20	7.5	19	4
Val			118.1	72.2	0.6	20	5	13	4
Thr			120.1	74.2	0.4	20	4.5	13	4
Ile+Leu			132.2	86.1	0.9	20	4.5	13	4
Asp			134.1	74.1	0.4	20	5.5	19	4
Glu			148.1	102.1	0.4	20	5.5	15	4
Met			150.2	104.1	0.7	20	4	13	4
His			156.2	110.1	0.4	20	5.5	17	4
Phe			166.2	120.2	2.1	20	6	17	4
Arg			175.1	70.1	0.4	20	6	31	4
Tyr			182.1	136.2	1.1	20	7	17	4
Asn			133.1	74.1	0.4	20	4.5	21	4
Gln			147.1	130	0.4	20	6	13	4
Trp			205.2	188.1	2.8	20	4.5	13	6
Lys			147.1	84.1	0.4	20	6	23	4
U-13C,15N-Ala			94.1	47.1	0.4	20	5.5	17	4
U-13C,15N-Ser			110	63.1	0.4	20	4.5	15	4
U-13C,15N-Pro			122.1	75	0.6	20	7.5	19	4
U-13C,15N-Val			124.1	77.2	0.6	20	4.5	15	4
U-13C,15N-Thr			125.1	78.2	0.4	20	5	13	4
U-13C,15N-Ile			139.2	92.1	0.9	20	4.5	13	4
U-13C,15N-Asp			139.1	77.1	0.4	20	10	19	4
U-13C,15N-Glu			154.1	107.1	0.4	20	5.5	15	4
U-13C,15N-Met			156.2	109.1	0.7	20	4	13	4
U-13C,15N-His			165.2	118.1	0.4	20	5.5	17	4
U-13C,15N-Phe			176.2	129.2	2.1	20	6	17	4
U-13C,15N-Arg			185.1	75.1	0.4	20	6	31	4
U-13C,15N-Tyr			192.1	145.2	1.1	20	7	17	4
U-13C,15N-Gln			154.1	136	0.4	20	6	13	4
U-13C,15N-Lys			155.1	90.1	0.4	20	6	23	4
D5-Trp			210	193	2.8	20	4.5	13	6

Supplementary Table 4. Chemical standards and compound information.

Compounds	Supplier	CAS number
Sinalbin	Isolated from *Sinapis alba* as described in Thies,1989[85]	Cas:19253-84-0
D,L-Sulforaphane (4MSOB ITC)	Biozol/TRC	Cas:4478-93-7
Allyl isothiocyanate	Sigma-Aldrich	Cas:57-06-7
Benzyl isothiocyanate	Sigma-Aldrich	Cas:622-78-6
2-Phenylethyl isothiocyanate	Sigma-Aldrich	Cas:2257-09-2
Butyl isothiocyanate	Aldrich	Cas:592-82-5
Isobutyl isothiocyanate	Alfa Aesar/Avocado Res.Chem.	Cas:591-82-2
sec-Butyl isothiocyanate	ABCR Karlsruhe	Cas:4426-79-3
Iberin (3MSOP ITC)	Santa Cruz Biotechnology	Cas: 505-44-2
Erucin (4MTB ITC)	Abcam Netherlands B.V.	Cas:4430-36-8
L-Glutathione reduced (GSH)	Sigma-Aldrich	Cas:70-18-8
L-Glutathione oxidized (GSSG)	Sigma-Aldrich	Cas:27025-41-8
Cys-Gly	Sigma-Aldrich	Cas:19246-18-5
L-Cysteine	Sigma-Aldrich	Cas:52-90-4
L-(+)-Lysine monohydrochloride	Duchefa	Cas:657-27-2
Amino acid standard	Sigma-Aldrich	Product No. AAS18
FMOC-chloride	Fluka	Cas:28920-43-6
D,L-Sulforaphane glutathione (4MSOB ITC-GSH)	Biozol/TRC	Cas:289711-21-3
4MSOB ITC-Cys-Gly	Synthesized as described in Schramm,2012[30] and Sun,2023[45]	Cas:877224-85-6
D,L-Sulforaphane-L-cysteine (4MSOB ITC-Cys)	Santa Cruz Biotechnology	Cas:364083-21-6
4MSOB ITC-Cyclic-Cys	This paper	Cas:1621991-12-5
4MSOB ITC-Lys	TRC	Cas:1211456-38-0
4MTB ITC-Cys-Gly	This paper	Cas:1656269-86-1
4MTB ITC-Cys	This paper	Cas:1656269-87-2
4MTB ITC-Cyclic-Cys	This paper	
Allyl ITC-GSH	Synthesized as described in Sun,2023[45]	Cas:179474-42-1
Allyl ITC-Cys	Synthesized as described in Sun,2023[45]	Cas:53329-93-4
Benzyl ITC-Cys-Gly	This paper	Cas:62959-31-3
Benzyl ITC-Cys	This paper	Cas:370065-67-1
Benzyl ITC-Lys	This paper	Cas:1211456-34-6

Compounds	Supplier	CAS number
2PE ITC-GSH	Synthesized as described in Sun,2023[45]	Cas:149415-79-2
2PE ITC-Cys-Gly	Synthesized as described in Sun,2023[45]	Cas:177715-39-8
2PE ITC-Cys	Synthesized as described in Sun,2023[45]	Cas:53330-02-2
2PE ITC-Cyclic-Cys	This paper	Cas:2365179-40-2
2PE ITC-Lys	TRC	Cas:1211456-36-8
Butyl ITC-Cys	This paper	
Benzonase nuclease	Merck KGaA	Cat. No. 70746-3
FastDigest BamHI	Thermo Fisher Scientific	Cat. No. FD0054
FastDigest XhoI	Thermo Fisher Scientific	Cat. No. FD0694
FastDigest SacI	Thermo Fisher Scientific	Cat. No. FD1134
FastDigest HindIII	Thermo Fisher Scientific	Cat. No. FD0505
Quick start Bradford 1× dye reagent	Bio-RAD	Cat. No. 5000205
Ni-NTA agrose resin	Qiagen	Cat. No. 30230
PageBlue protein staining solution	Thermo Fisher Scientific	Cat. No. 24620
Protease-inhibitor mix HP	Serva Electrophoresis	Cat. No. 39106.03
TRIzol reagent	Invitrogen	Cat. No. 15596026
DEAE-Sephadex A-25 columns	Sigma-Aldrich	Cas:12609-80-2
Sulfatase from Helix pomatia	Sigma-Aldrich	Cas:9016-17-5
Phusion DNA Polymerase	Thermo Fisher Scientific	Cat. No. F630L
T4 DNA Ligase (5 U/μL)	Invitrogen	Cat. No. EL0011
Bovine serum albumin standard pre-diluted set	Thermo Fisher Scientific	Cat. No. 23208
RNase-free DNase set	Qiagen	Cat. No. 79254
SuperScript III reverse transcriptase kits	Invitrogen	Cat. No. 18080093
Brilliant III ultra-fast SYBR green QPCR master mix	Agilent Technologies	Cat. No. 600883

Supplementary Table 5. Basic information of GST genes and GST proteins.

Insect	Class	Name	Genbank	Length	Lenght	MW	AlphaFold protein structure database		
			Accession	(bp)	(aa)	(kDa)	protein	profile	pLDDT score
Spodoptera littoralis	δ	SlGSTD1	MH177577	666	221	24.3			
		SlGSTD2	PQ722167	738	245	27.6			
		SlGSTD3	PQ722168	672	223	25	Glutathione S-transferase delta 4	AF-A0A075X3S8-F1-v4	97.5
	ε	SlGSTE1	MH177580	654	217	23.9			
		SlGSTE2	PQ722169	672	223	24.6			
		SlGSTE3	PQ722170	669	222	25.1			
		SlGSTE4	PQ722171	693	230	26.1			
		SlGSTE5	PQ722172	687	228	26.2			
		SlGSTE6	PQ722173	603	200	22.1			
		SlGSTE7	PQ722174	699	232	26.5			
		SlGSTE8	PQ722175	657	218	25			
		SlGSTE9	PQ722176	654	217	24.4	Glutathione S-transferase epsilon 9	AF-A0A075X2I7-F1-v4	97.06
		SlGSTE10	PQ722177	654	217	24.6			
		SlGSTE11	PQ722178	702	233	26.2			
		SlGSTE12	PQ722179	657	218	25			
		SlGSTE13	PQ722180	654	217	25.1			
		SlGSTE14	PQ722181	654	217	25.1			
		SlGSTE15	PQ722182	657	218	24.8			
		SlGSTE16	PQ722183	669	222	25.6			
		SlGSTE17	PQ722184	672	223	24.6			

Insect	Class	Name	Genbank	Length	Lenght	MW	AlphaFold protein structure database		
			Accession	(bp)	(aa)	(kDa)	protein	profile	pLDDT score
Spodoptera littoralis	ζ	SlGSTZ1	PQ722164	663	220	25.2			
		SlGSTZ2	PQ722165	639	212	24.3			
	θ	SlGSTT1	PQ722166	687	228	26.4			
	σ	SlGSTS1	MH177603	606	201	22.8			
		SlGSTS2	PQ722158	615	204	23.2			
		SlGSTS3	PQ722159	615	204	23.6			
		SlGSTS4	MH177606	615	204	23.1			
		SlGSTS5	PQ722160	618	205	24.1	Glutathione S-transferase sigma 5	AF-A0A075X3S3-F1-v4	94.75
		SlGSTS6	PQ722161	639	212	24.6	Glutathione-S-transferase sigma class	AF-A0A3G1ZLD9-F1-v4	94.06
	ω	SlGSTO1	PQ722162	765	254	29			
		SlGSTO2	PQ722163	849	282	32.6			
		SlGSTO3	MH177602	723	240	28.7			
	NC	SlGSTU1	MH177610	699	232	27.2			
		SlGSTU2	MH177611	645	214	23.8			
Drosophila melanogaster	ε	DmGSTE7	NP_611329.1						
	δ	DmGSTD1	NP_524326.1						
		DmGSTD2	NP_524912.1						
Scaptomyza flava	δ	SfGSTD1	AGY31886.1						
Scaptomyza nigrita	δ	SnGSTD1	AGL93472.1						
Scaptomyza caliginosa	δ	ScGSTD1	AGL93467.1						
Spodoptera litura	ε	SlitGSTE1	AAS79891.1						

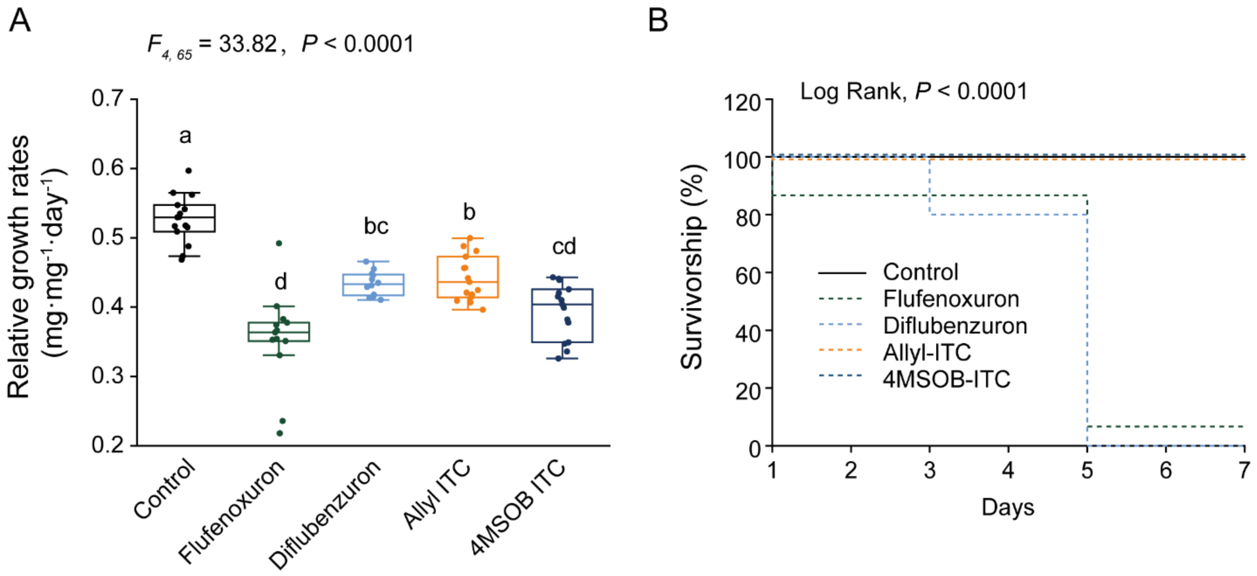

Supplementary Figure 1 Inhibition of *Spodoptera littoralis* larval growth by isothiocyanate (ITC) and insecticides. (**A**) Larval growth of *S. littoralis* was negatively affected by the presence of insecticides in the artificial diets (n = 15), with flufenoxuron causing a significant reduction in growth. Relative growth rates ($mg\cdot mg^{-1}\cdot day^{-1}$) were calculated as the natural logarithm (Ln) of final mass minus initial mass, divided by the number of feeding days (4 days for all treatments). (**B**) Larval survivorship was significantly affected by insecticide exposure (n = 15). Abbreviations: 4MSOB ITC, 4-methylsulfinylbutyl isothiocyanate. Box plots show the interquartile range (25th to 75th percentile), with median values indicated by horizontal lines and whiskers representing data range and outliers. Significant differences among means (±SE) in panel **A** were determined by one-way ANOVA followed by Tukey's HSD test. Survival analysis in panel **B** was performed using Kaplan–Meier survival estimates. Different lowercase letters indicate statistically significant differences (P < 0.05).

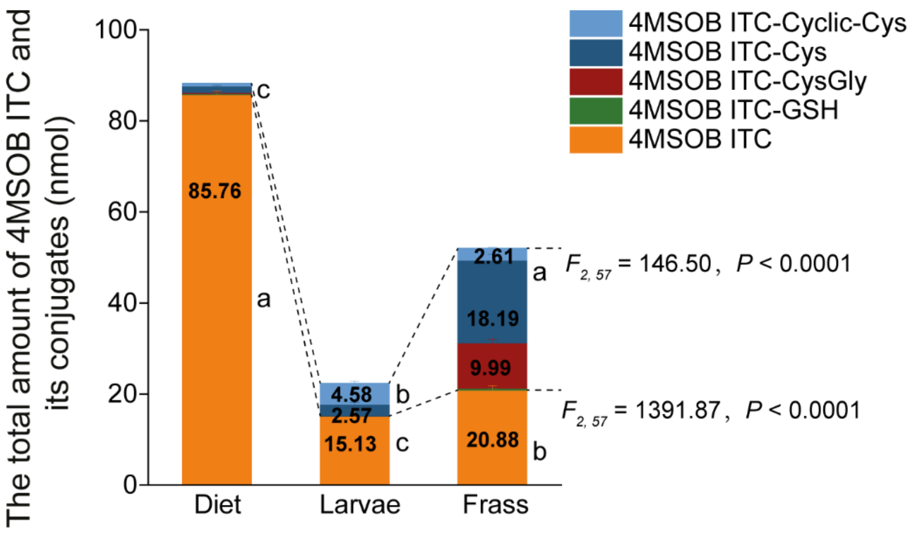

Supplementary Figure 2 Quantitative conversion of 4MSOB ITC into its conjugates during *S. littoralis* feeding. Total amounts of 4MSOB ITC and its conjugates were measured in the consumed diet, *S. littoralis* larvae, and collected frass (n = 20). Bold numbers in graph represent the mean values of 4MSOB ITC and its conjugates. Detailed measurements are provided in the raw data file. Statistical differences among means (±SE) of 4MSOB ITC or its conjugates were assessed using one-way ANOVA followed by Tukey's HSD test. Different lowercase letters indicate statistically significant differences (P < 0.05).

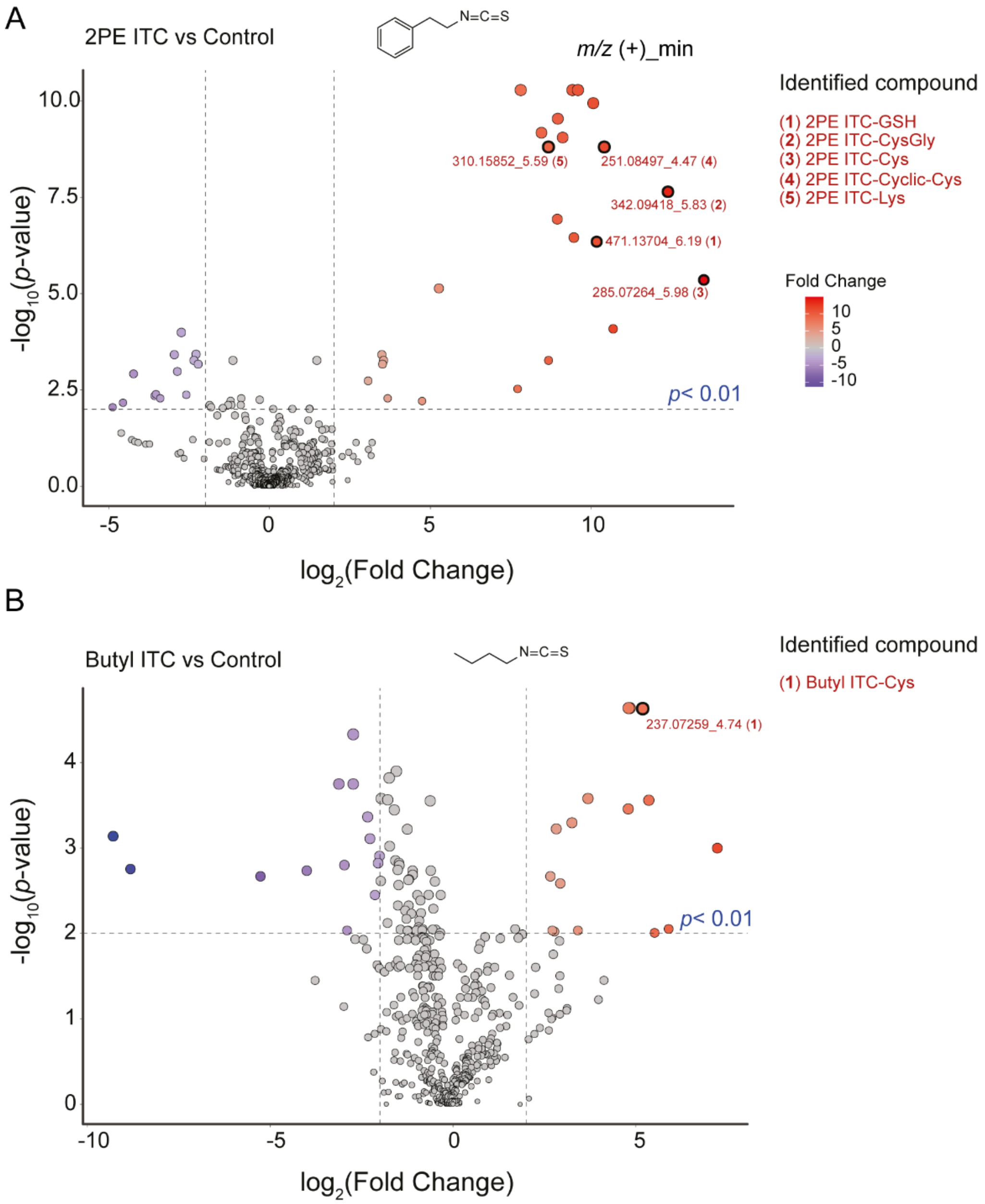

Supplementary Figure 3 Metabolism of ITCs by *S. littoralis* via the mercapturic acid pathway and lysine conjugation. Volcano plots of extracted LC-MS/MS features from non-targeted Q-TOF (UHPLC-HRMS, positive mode) analyses of *S. littoralis* larvae fed on an artificial diet containing 2PE ITC (A) or butyl ITC (B) compared to larvae that fed on an artificial diet without ITC (negative control). $n = 5$ for each treatment. Detailed features with significant differences are listed in Supplementary Data 7. Significant differences between means (±SE) were determined by two sample t-tests ($P < 0.01$).

^{1}H NMR with water presaturation full range in MeOH-d_3, 238 K

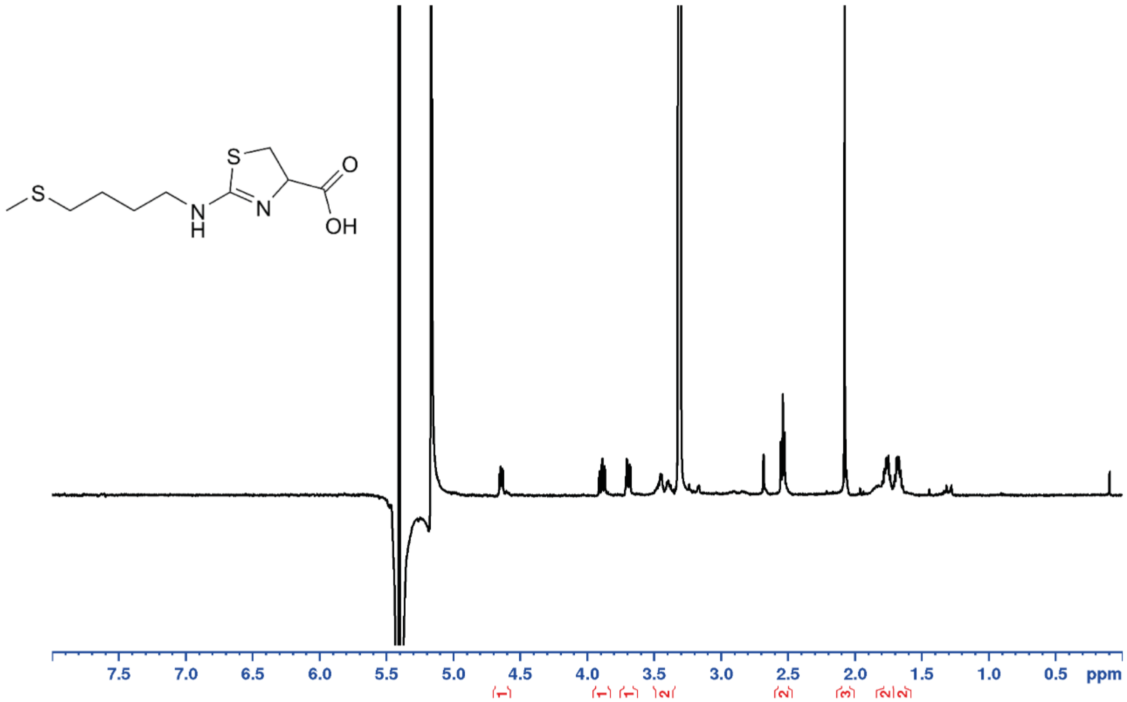

[13]C full range in MeOH-d_3, 238 K

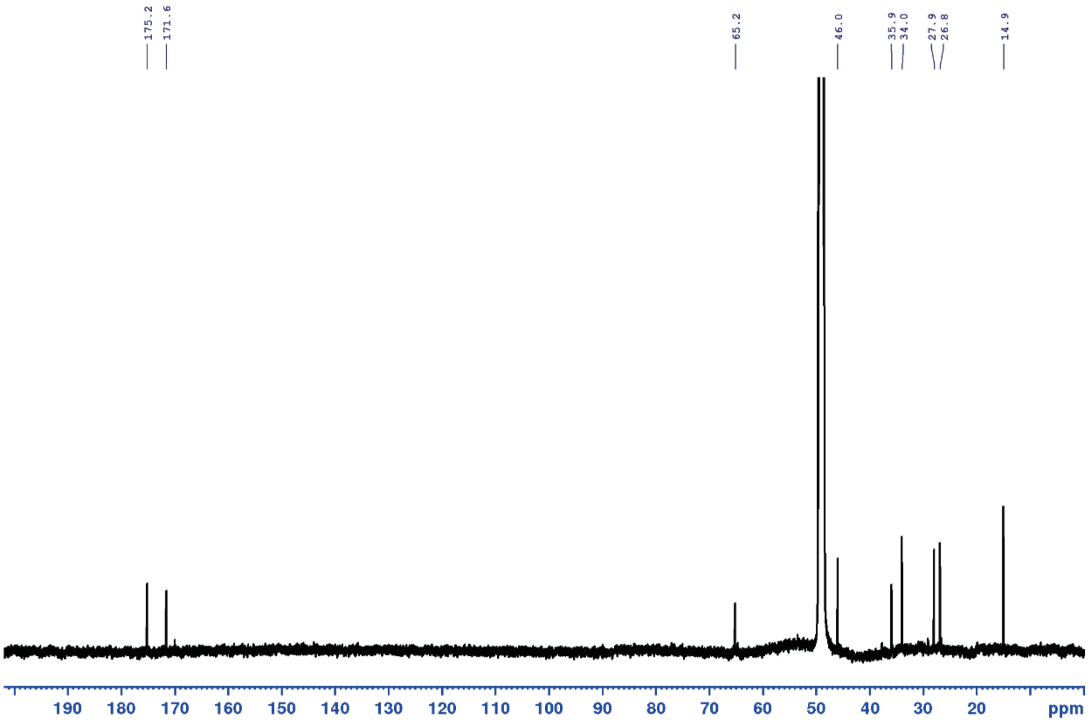

Supplementary Figure 4a NMR spectra for 4MTB ITC-Cyclic-Cys

^{1}H NMR with water presaturation full range in MeOH-d_3, 238 K

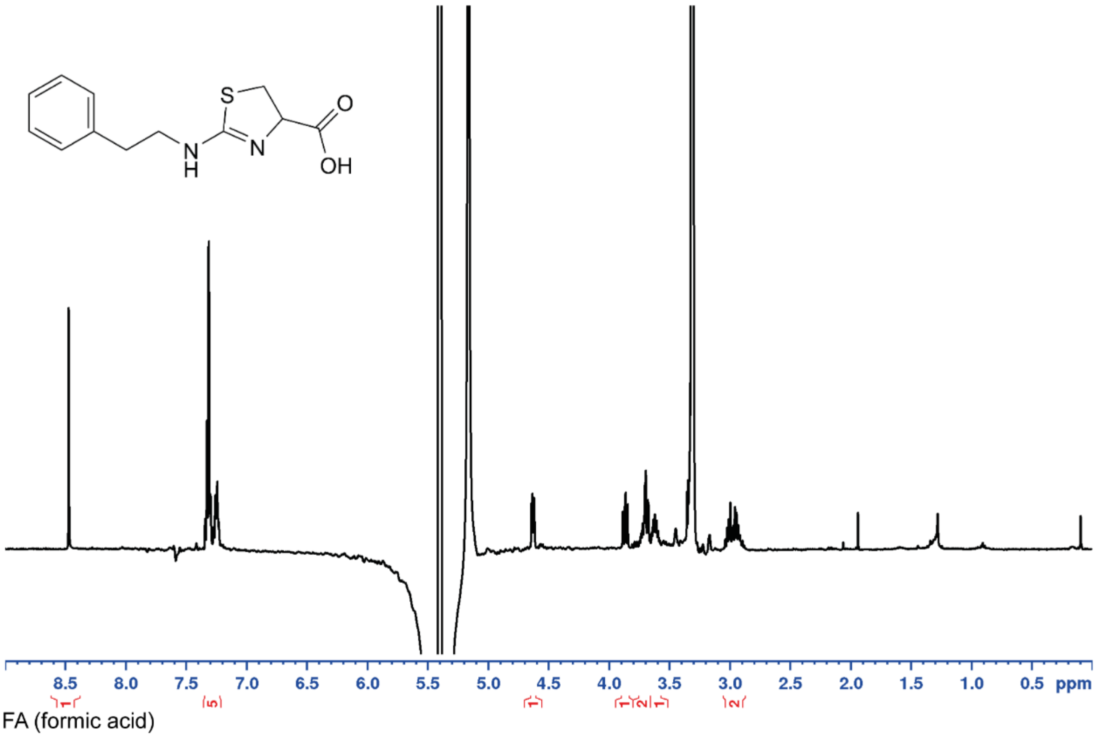

^{13}C full range in MeOH-d_3, 238 K

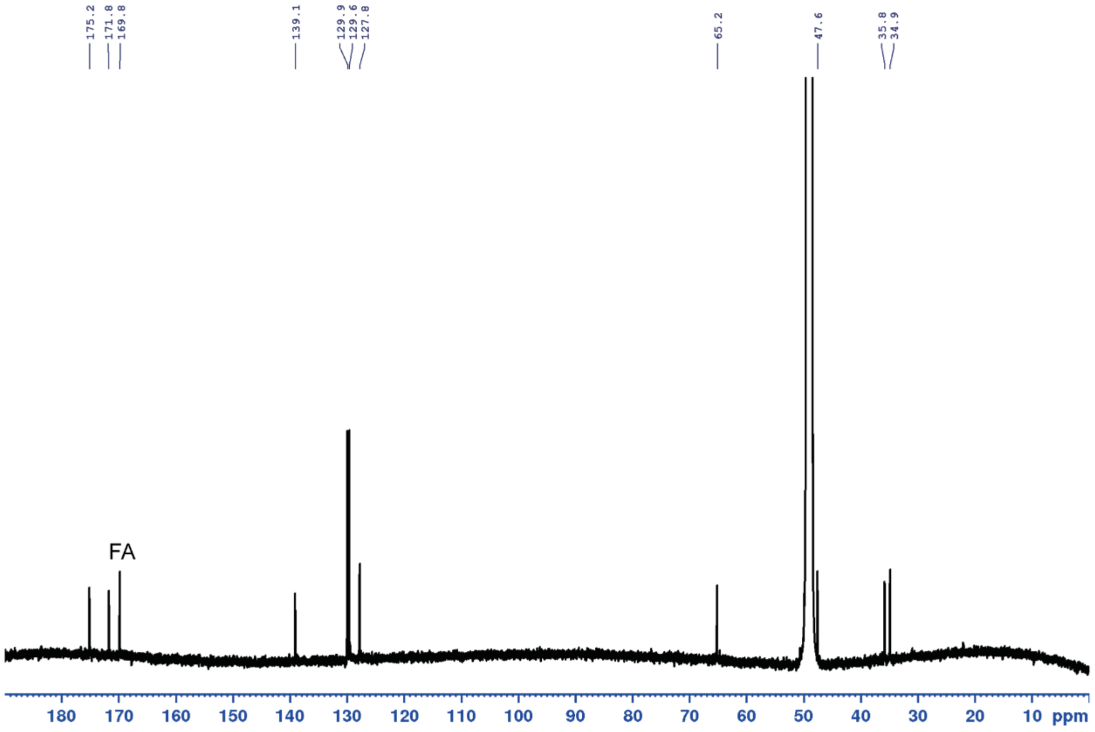

Supplementary Figure 4b NMR spectra for 2PE ITC-Cyclic-Cys

^1H NMR with water presaturation full range in D_2O, 298 K

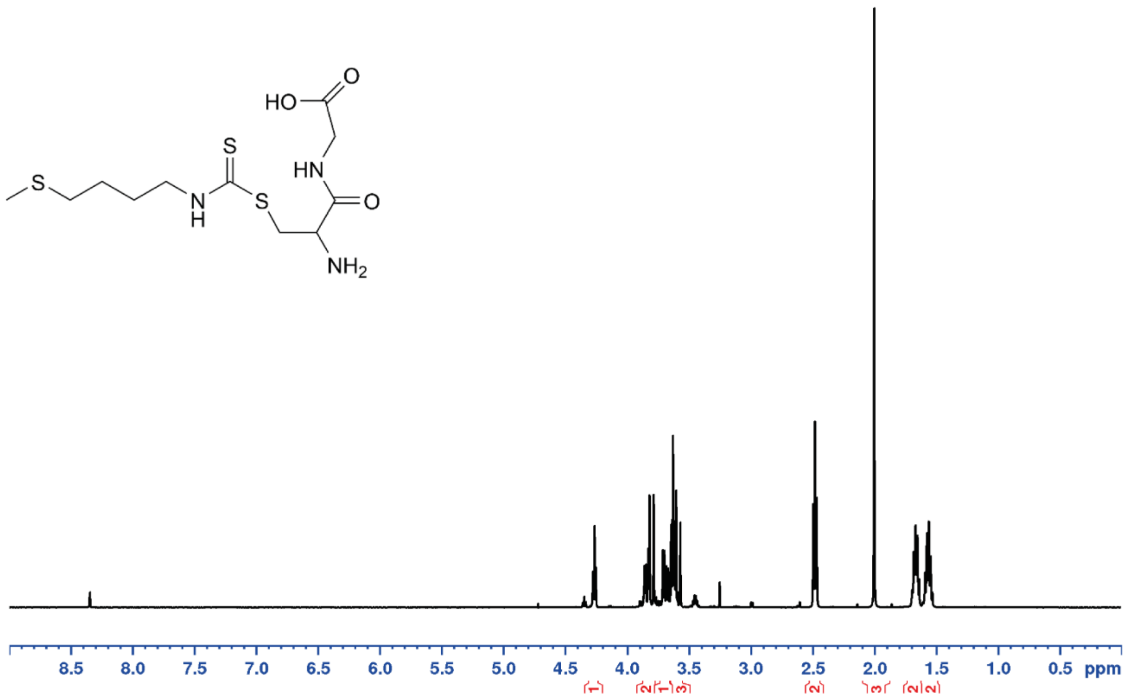

DEPTQ full range in D_2O, 298 K

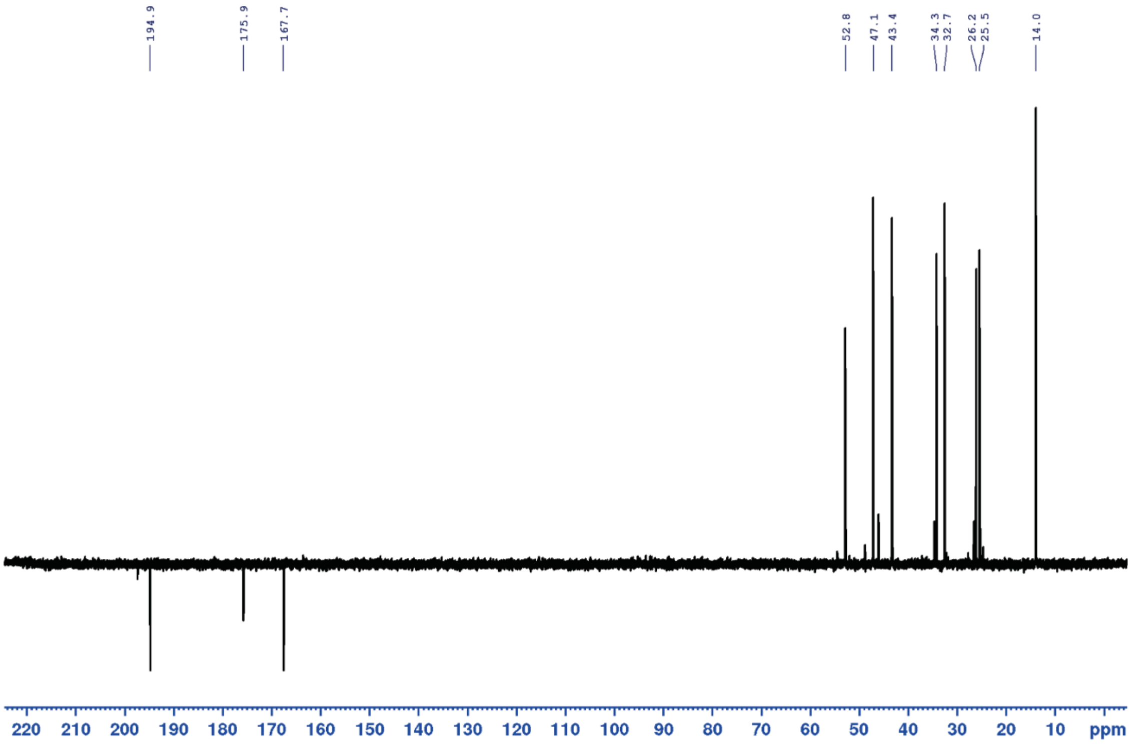

Supplementary Figure 4c NMR spectra for 4MTB ITC-CysGly

^{1}H NMR with water presaturation full range in D_2O, 298 K

Due to impurities and water presaturation, some integrals do not match with the structure

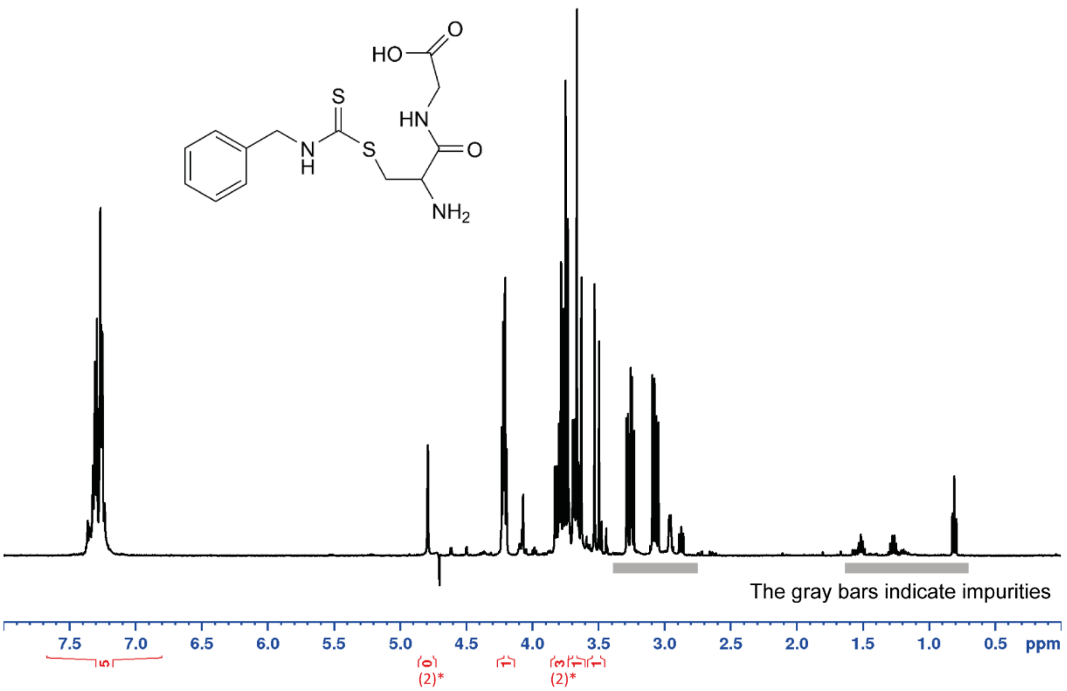

*The value in parentheses is the integral value expected from the structure

DEPTQ full range in D_2O, 298 K

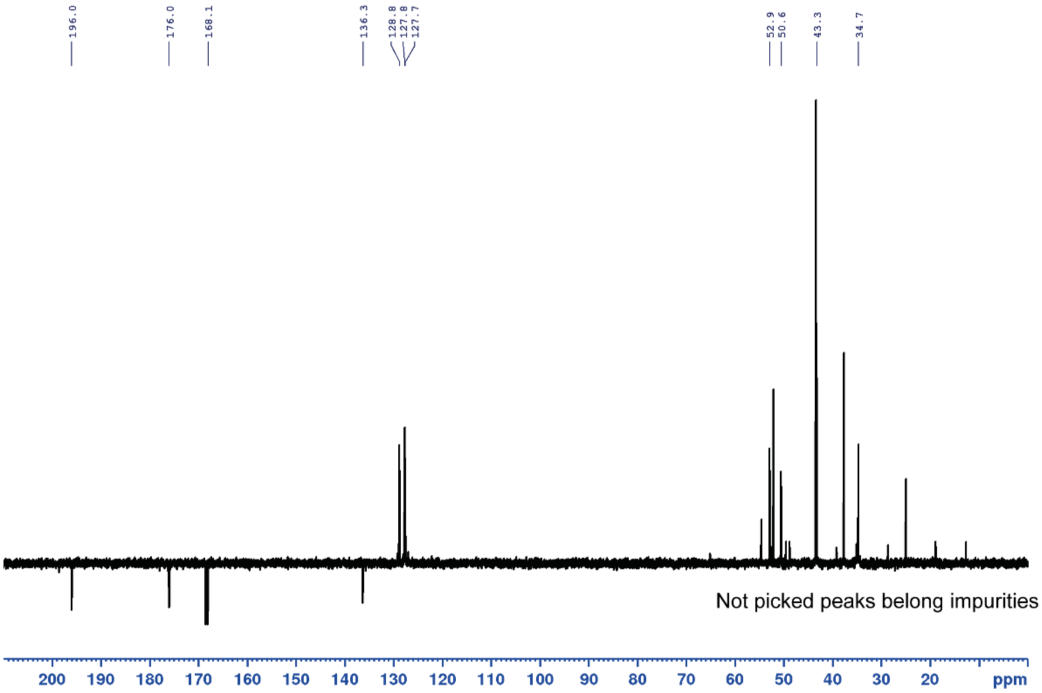

Supplementary Figure 4d NMR spectra for Benzyl ITC-CysGly

^{1}H NMR with water presaturation full range in D_2O, 298 K

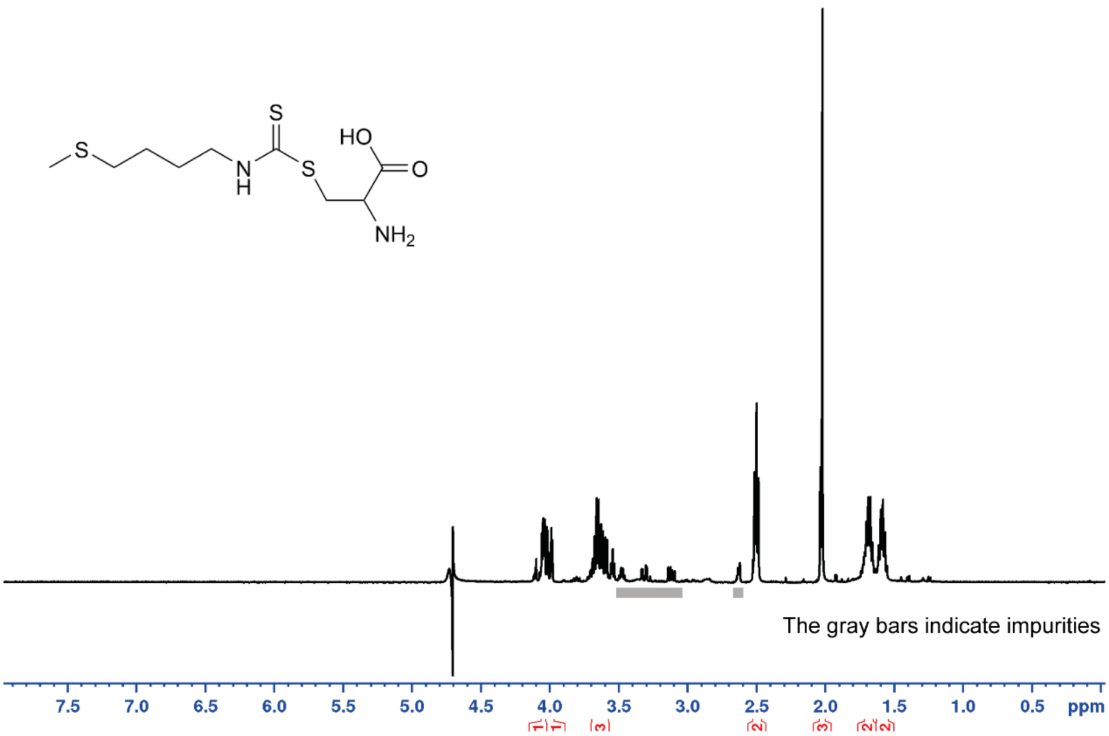

DEPTQ full range in D_2O, 298 K

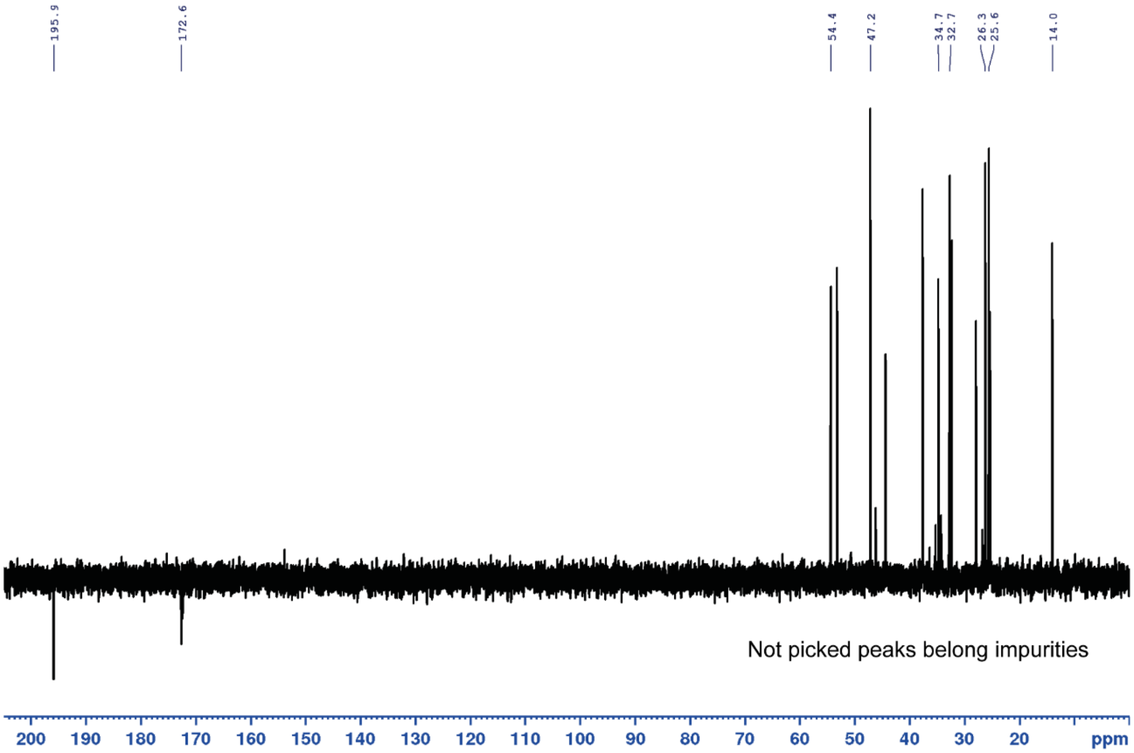

Supplementary Figure 4e NMR spectra for 4MTB ITC-Cys

^1H NMR with water presaturation full range in MeOH-d_3, 298 K

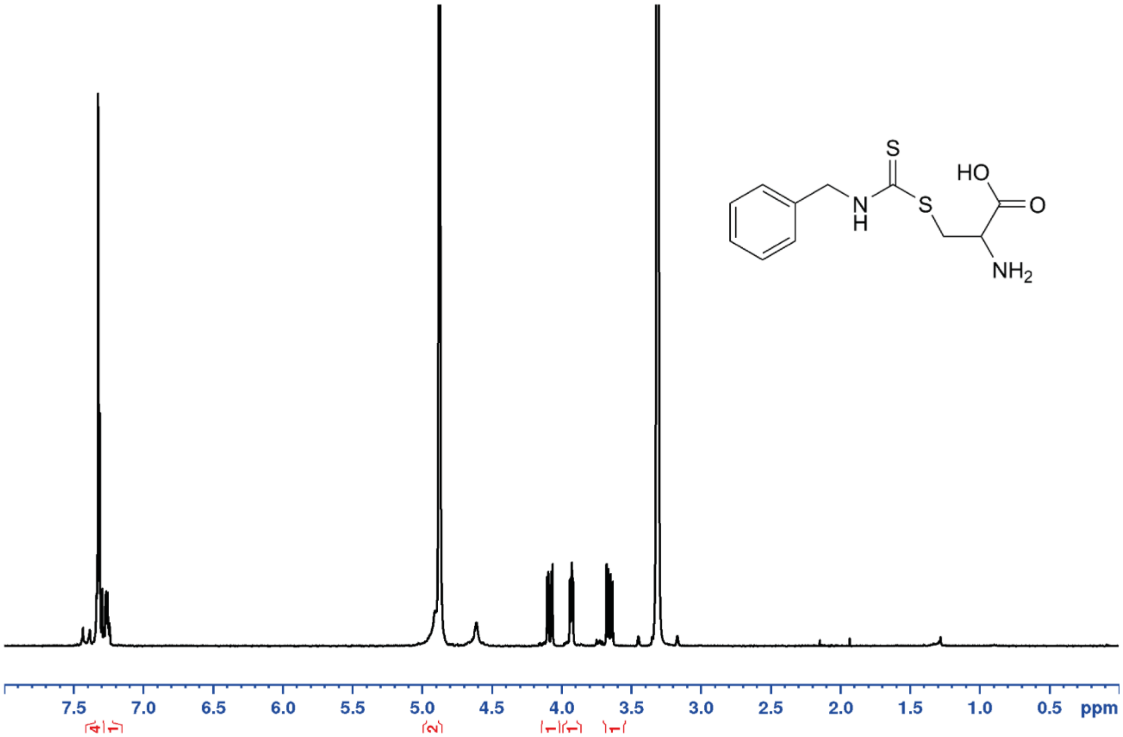

^{13}C full range in MeOH-d_3, 298 K

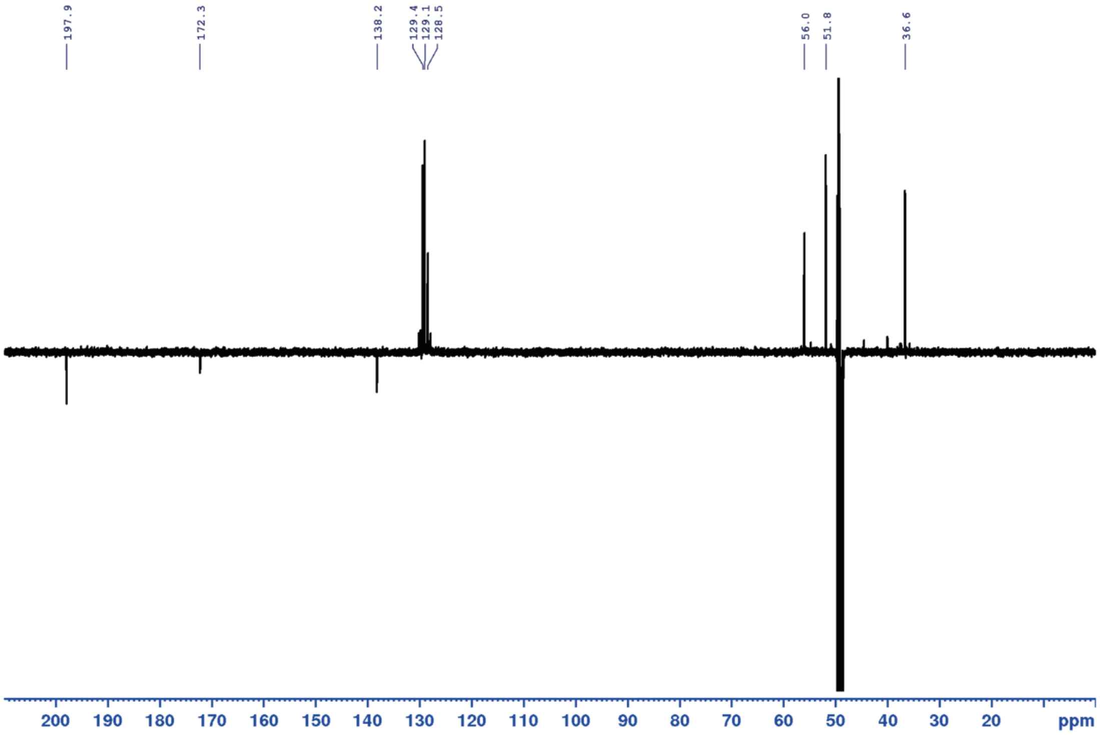

Supplementary Figure 4f NMR spectra for Benzyl ITC-Cys

^{1}H NMR with water presaturation full range in D_2O, 298 K

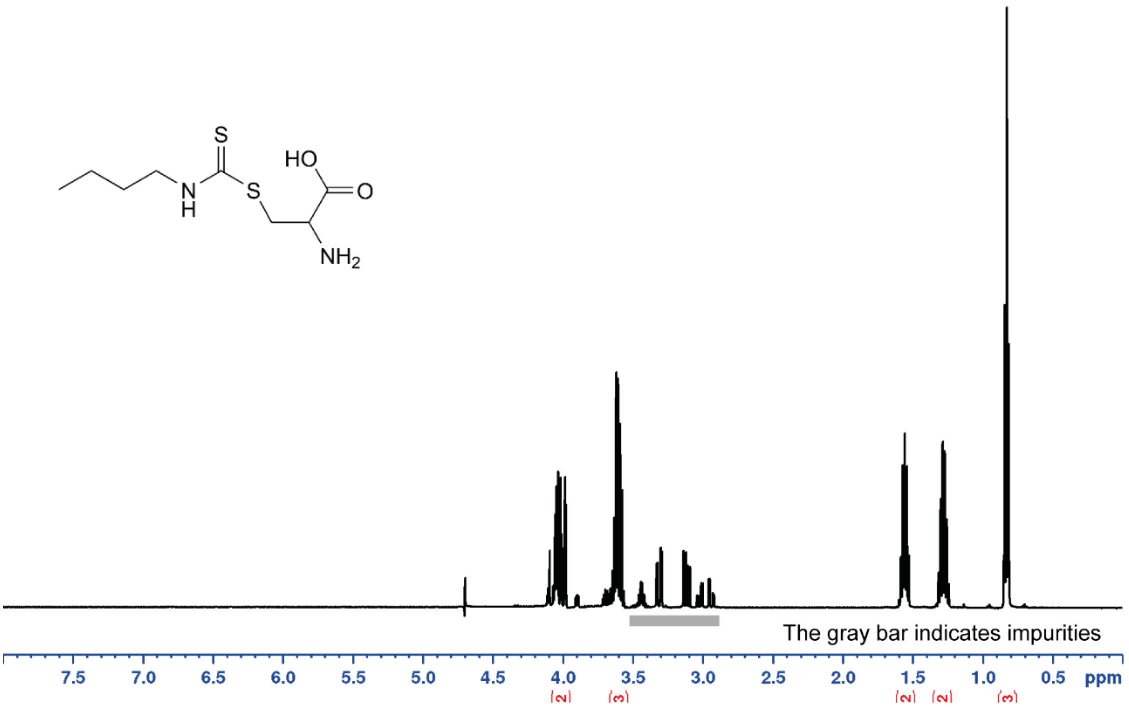

DEPTQ full range in D_2O, 298 K

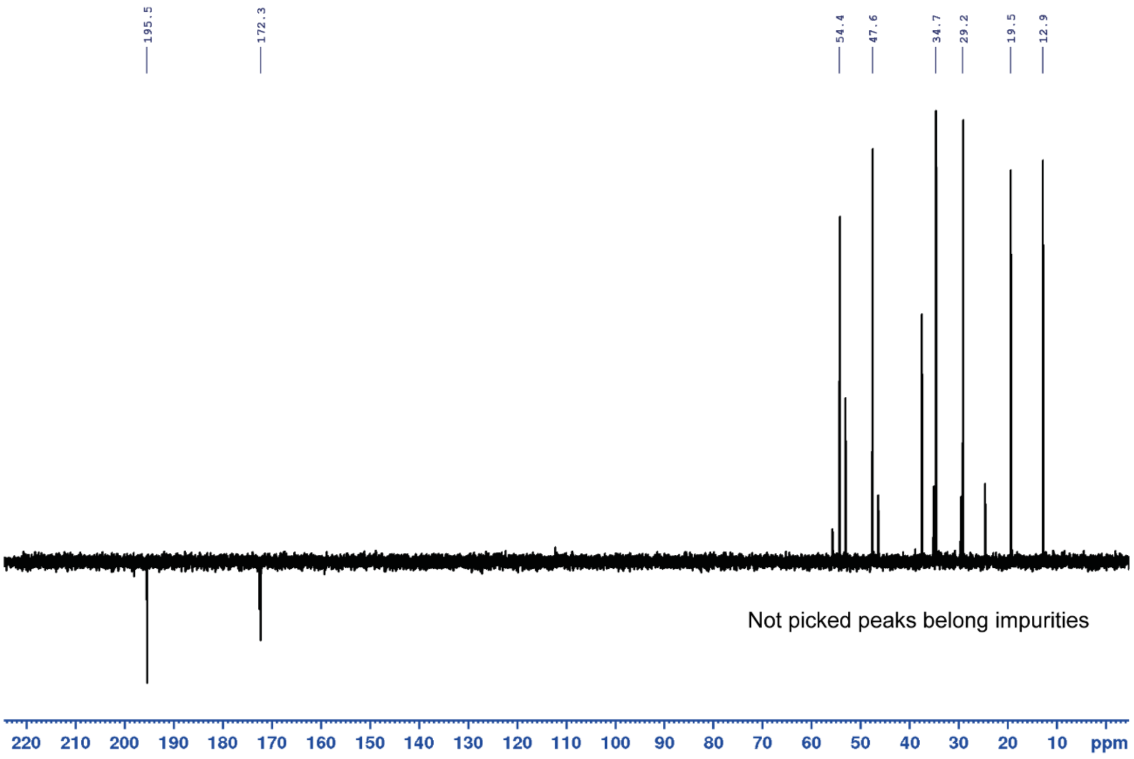

Supplementary Figure 4g NMR spectra for Butyl ITC-Cys

^{1}H NMR with water presaturation full range in MeOH-d_3, 298 K

Due to very broad signals, some integrals do not match with the structure

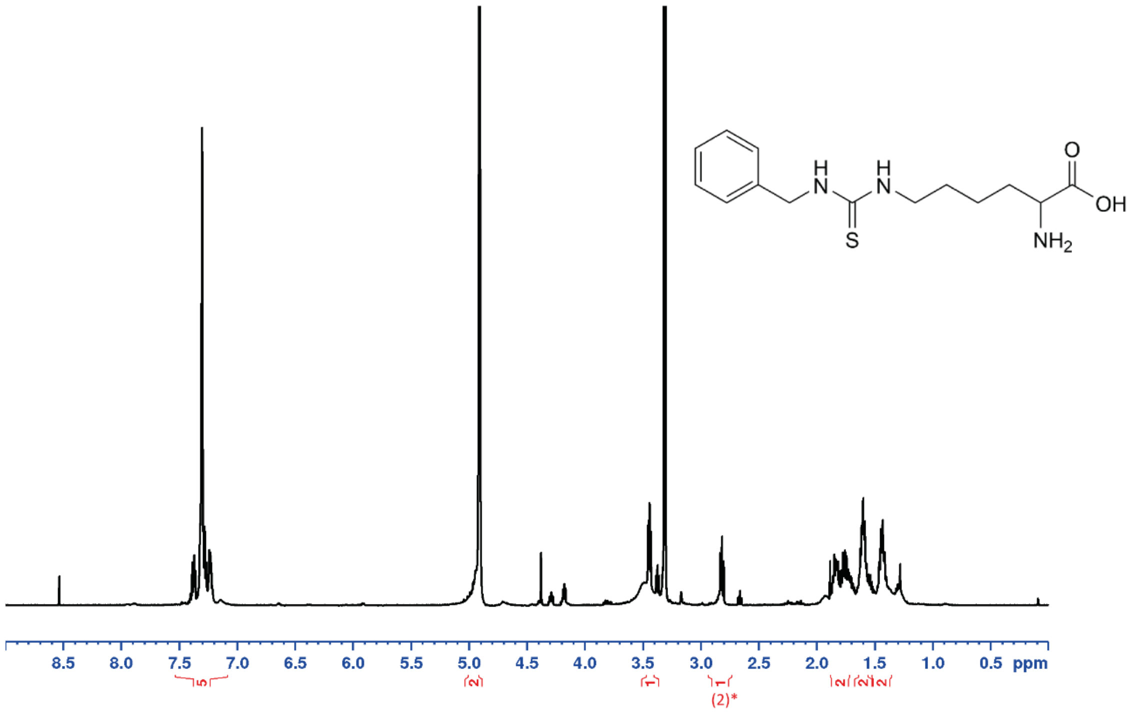

*The value in parentheses is the integral value expected from the structure

^{13}C full range in MeOH-d_3, 298 K

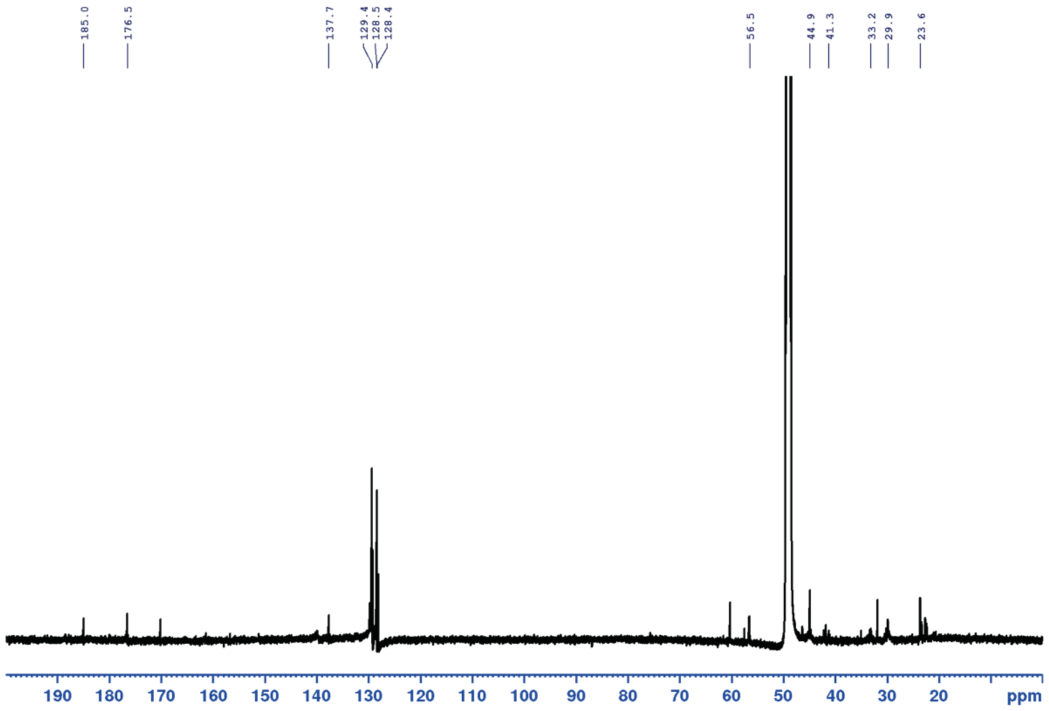

Supplementary Figure 4h NMR spectra for Benzyl ITC-Lys

Supplementary Figure 5

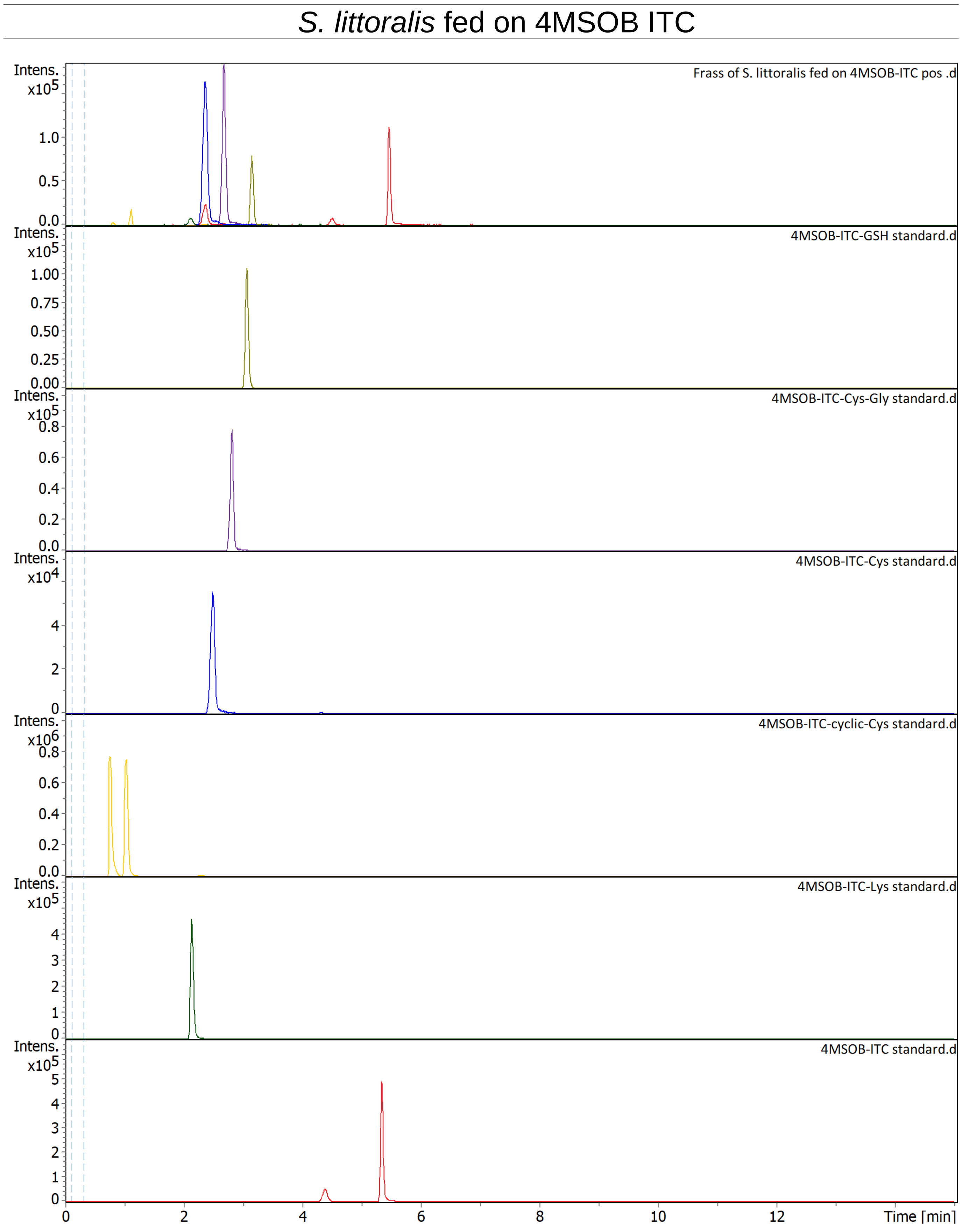

4MSOB ITC-GSH

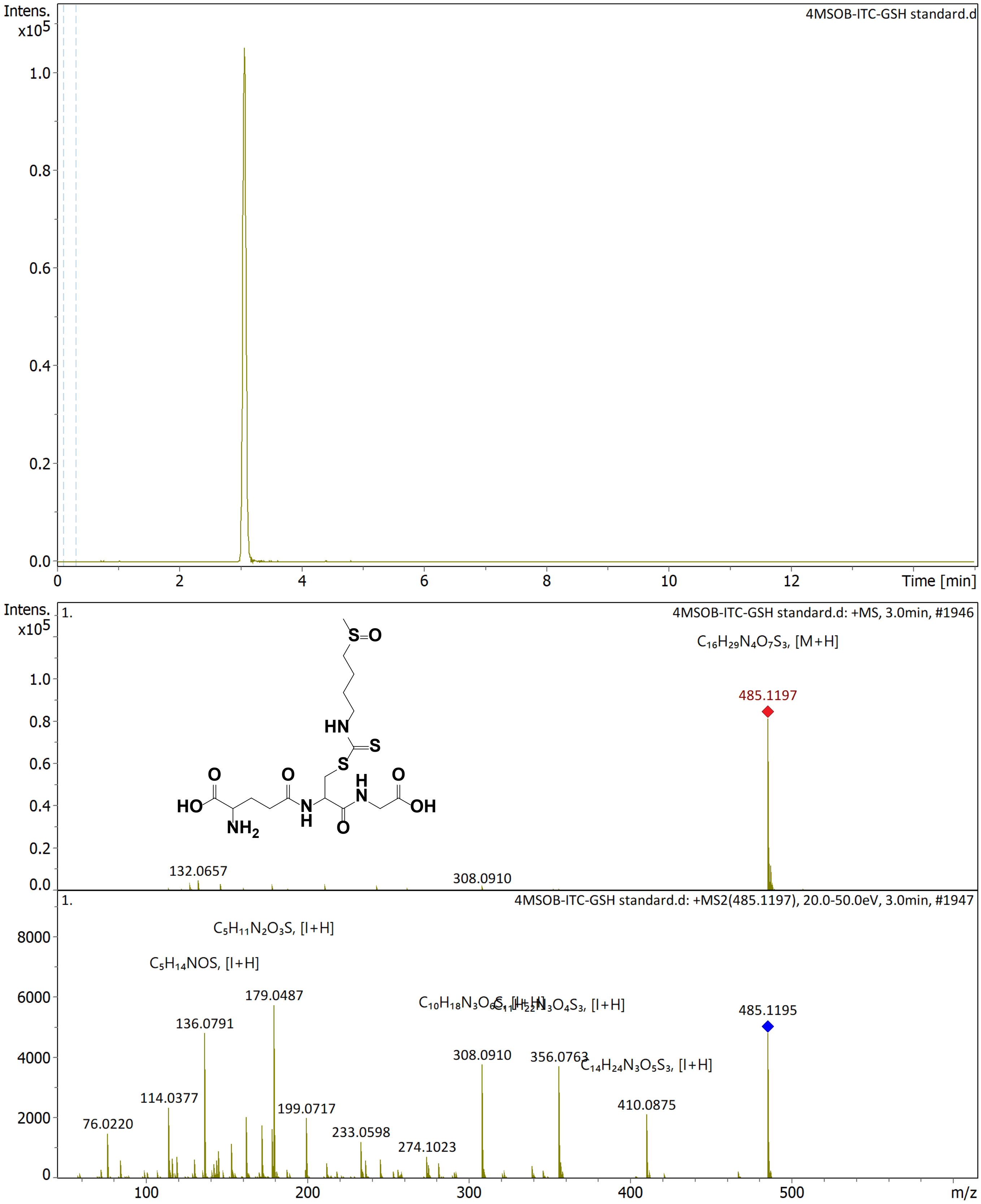

4MSOB ITC-CysGly

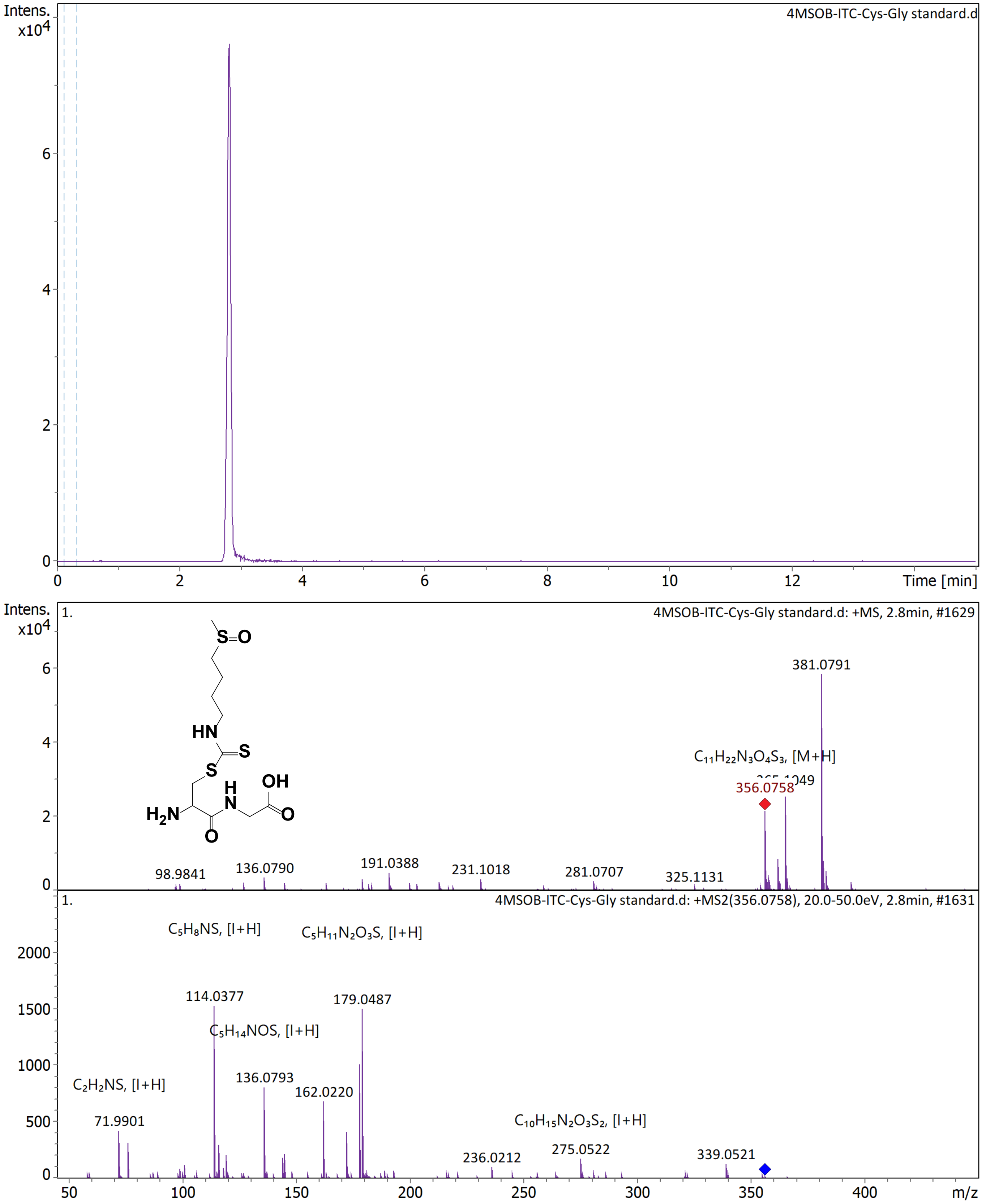

4MSOB ITC-Cys

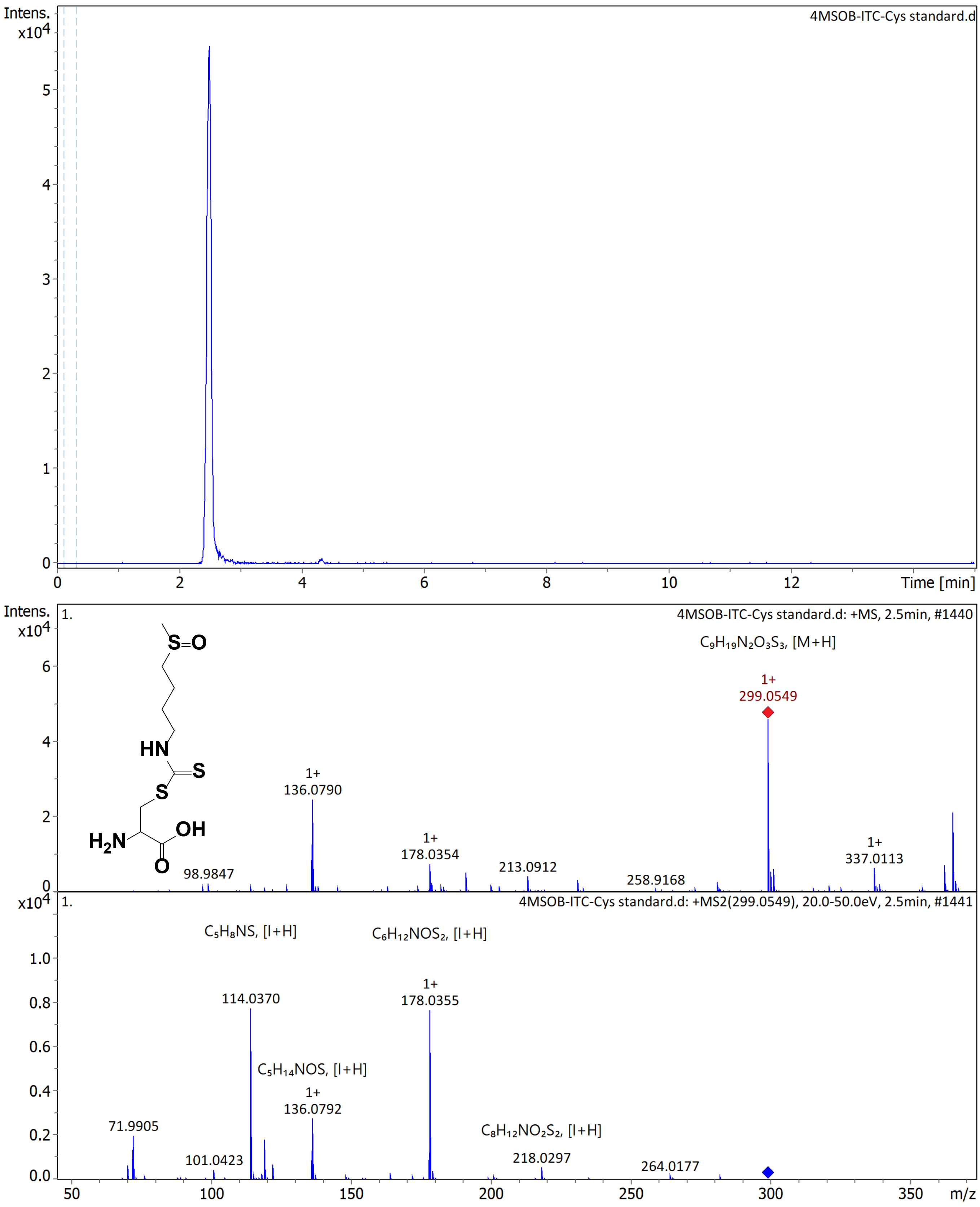

4MSOB ITC-Cyclic-Cys

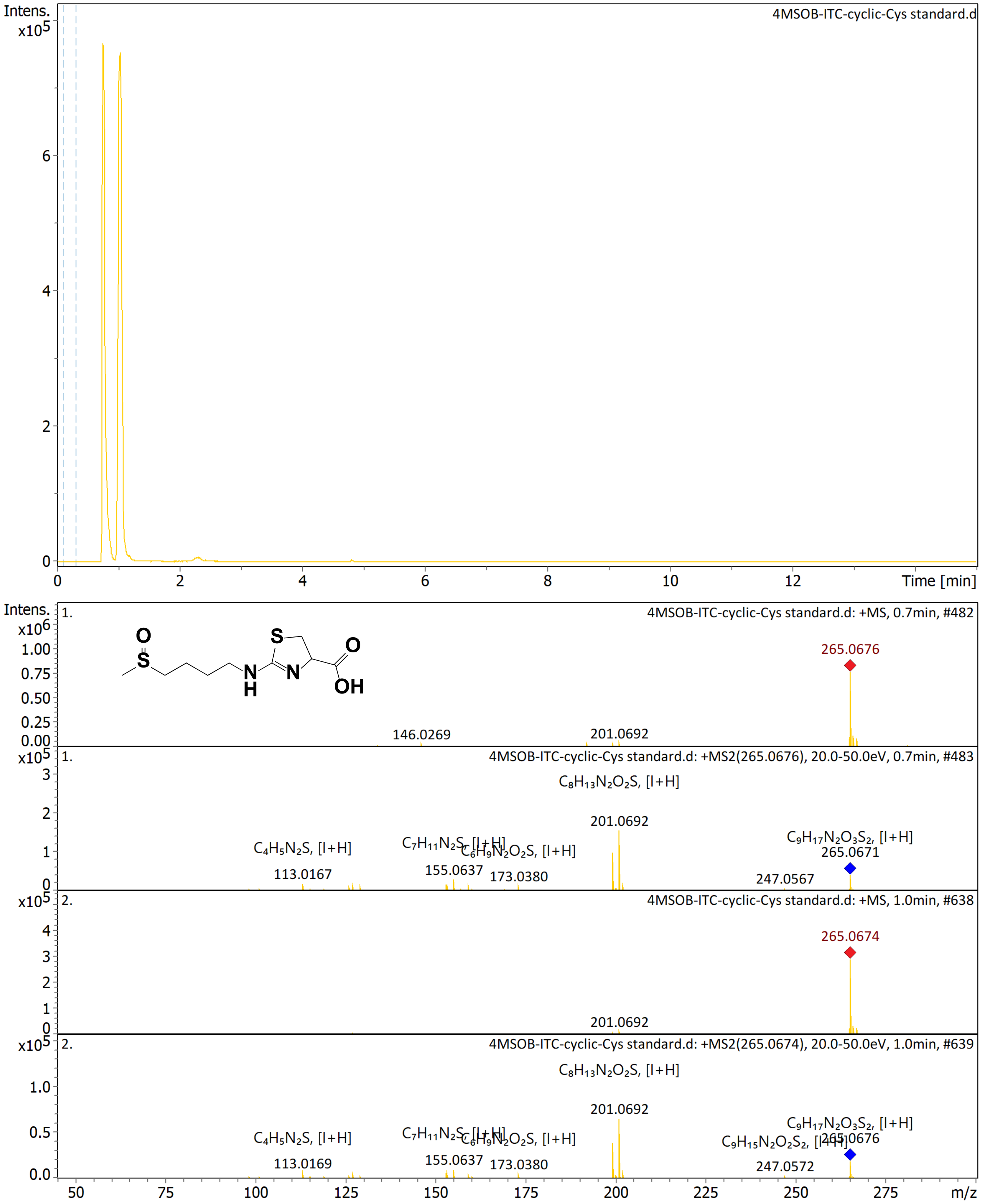

4MSOB ITC-Lys

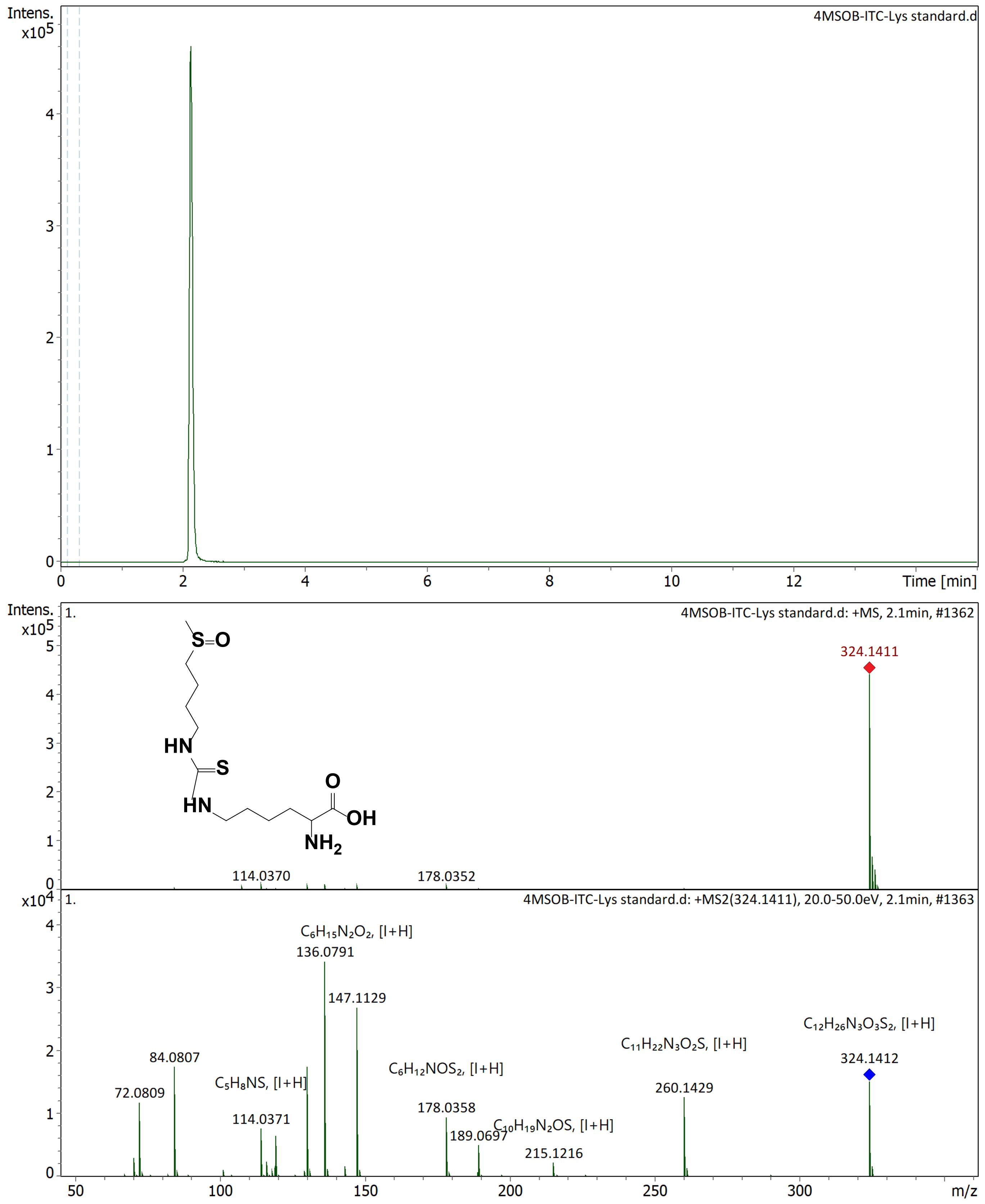

4MSOB ITC

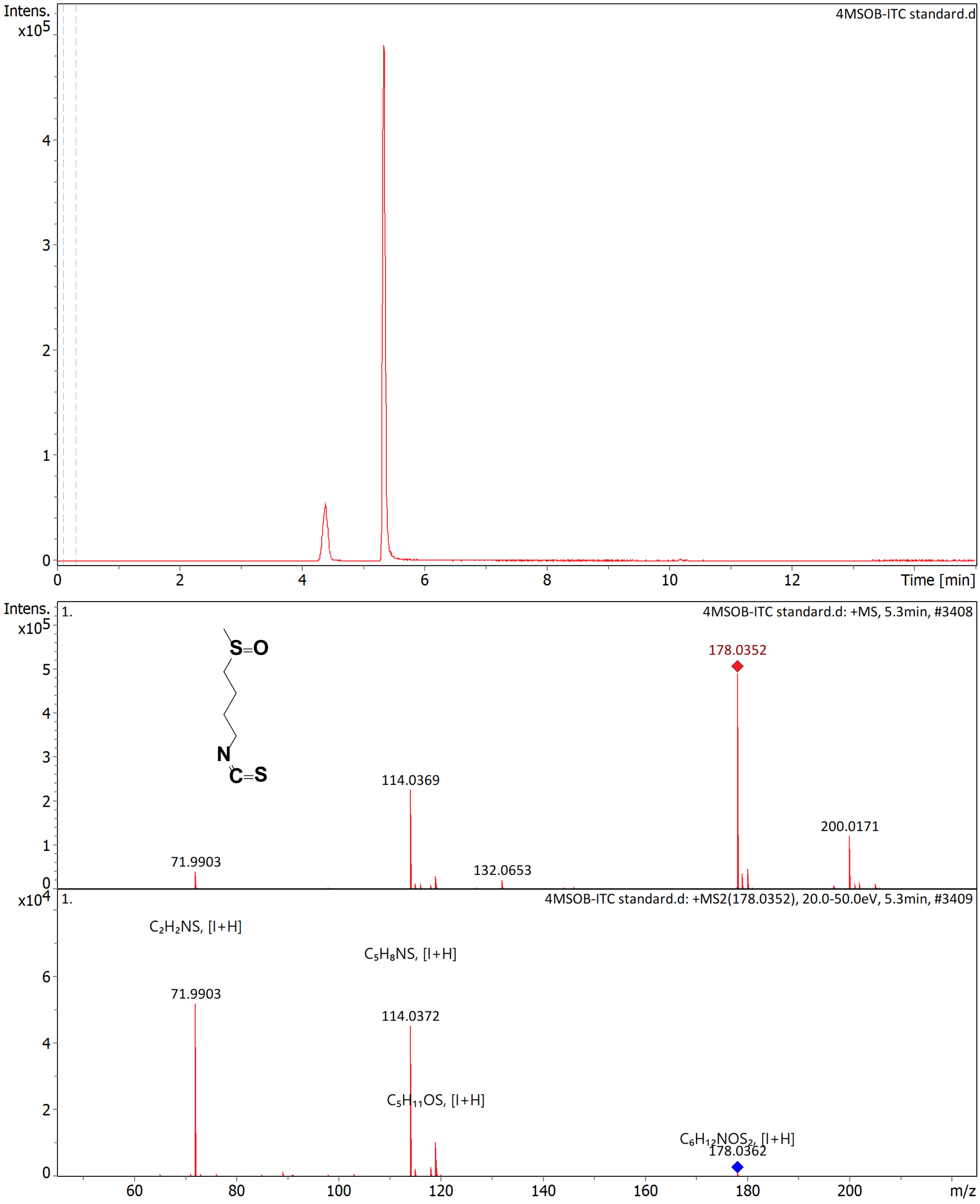

Intens.
x10^5
4MSOB-ITC standard.d
Time [min]
1.
4MSOB-ITC standard.d: +MS, 5.3min, #3408
S=O
N
C=S
178.0352
114.0369
200.0171
71.9903
132.0653
x10^4
4MSOB-ITC standard.d: +MS2(178.0352), 20.0-50.0eV, 5.3min, #3409
C2H2NS, [I+H]
C5H8NS, [I+H]
71.9903
114.0372
C5H11OS, [I+H]
C6H12NOS2, [I+H]
178.0362
m/z

S. littoralis fed on 4MSOB ITC

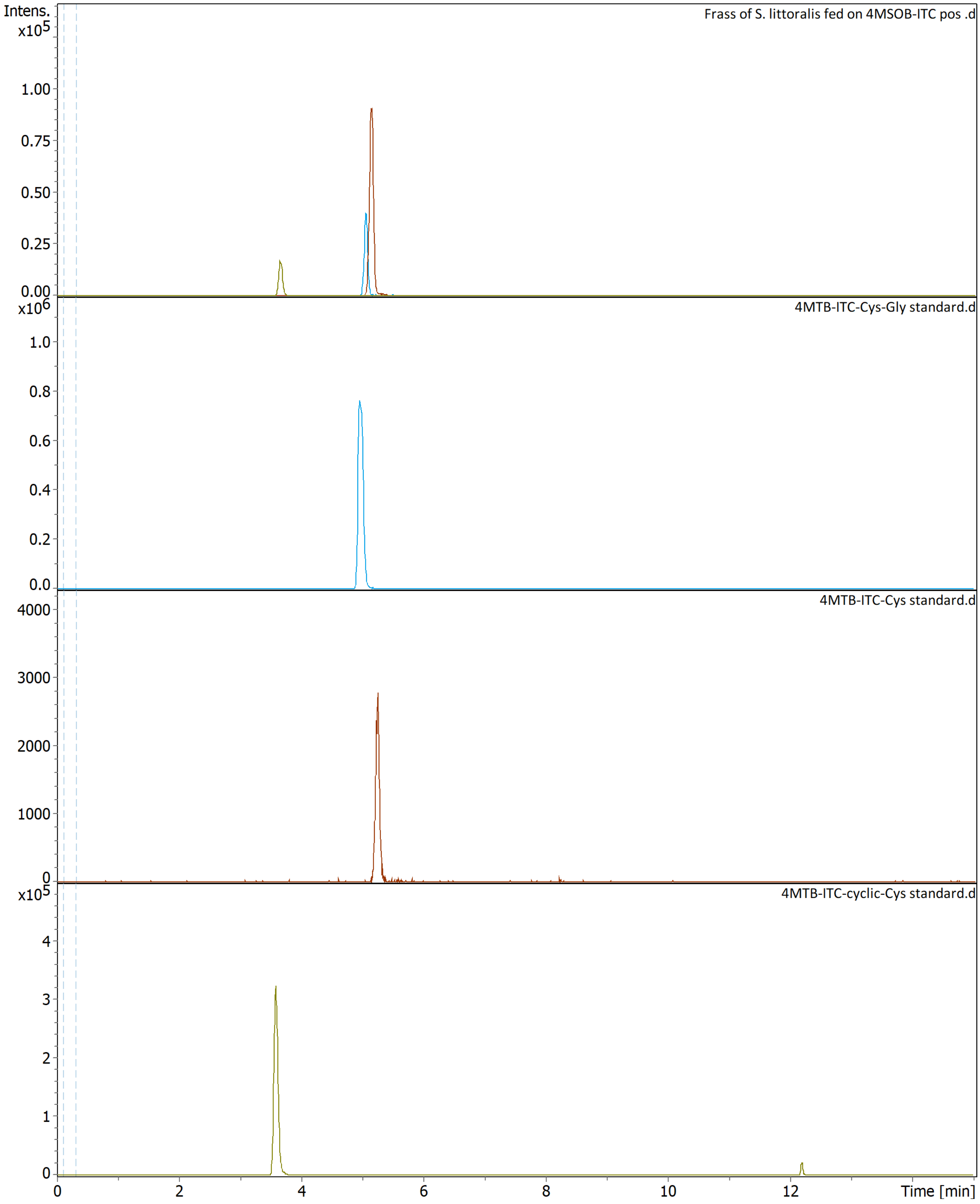

4MTB ITC-CysGly

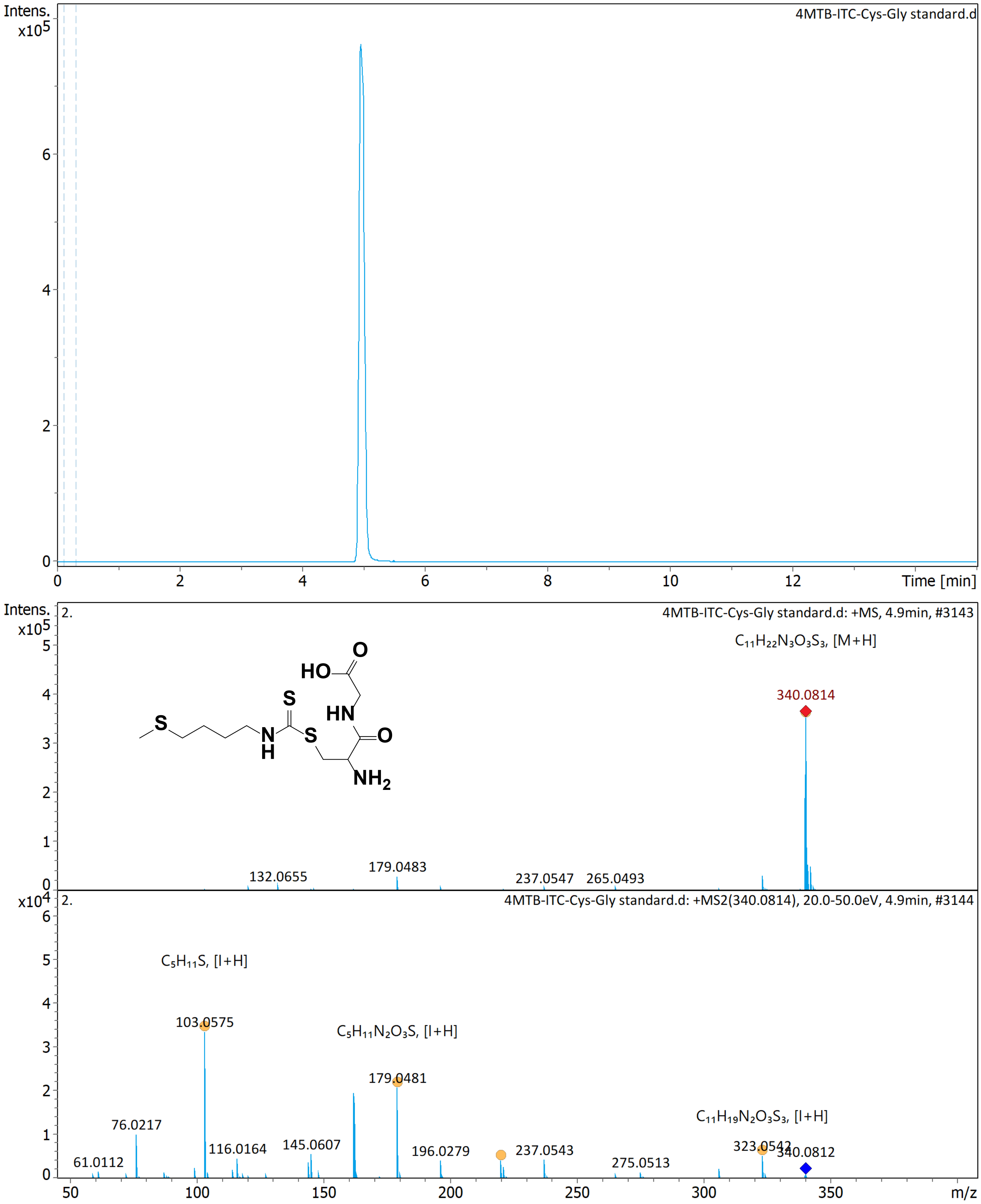

4MTB ITC-Cys

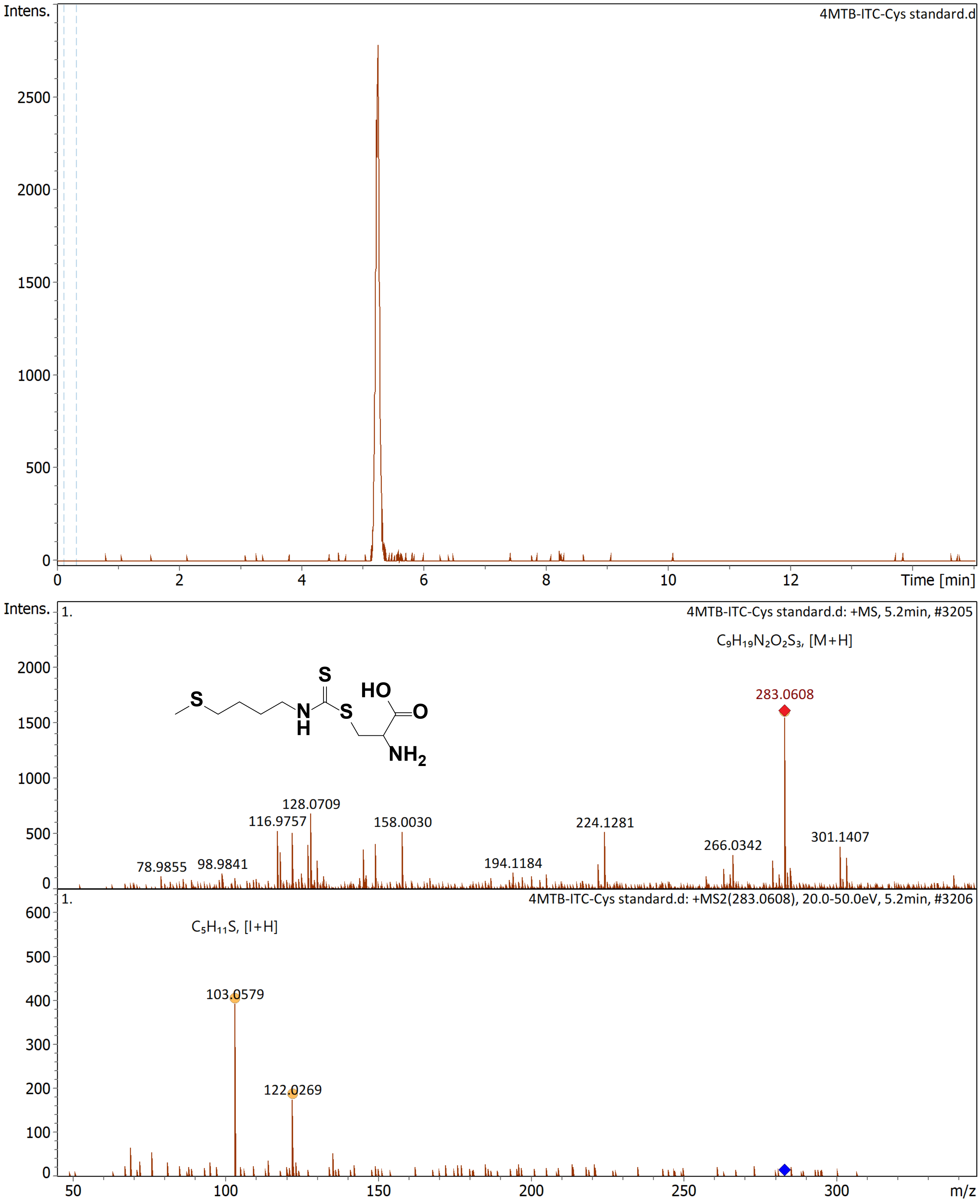

Intens.
4MTB-ITC-Cys standard.d
Time [min]
1.
4MTB-ITC-Cys standard.d: +MS, 5.2min, #3205
C9H19N2O2S3, [M+H]
283.0608
128.0709
116.9757
158.0030
224.1281
266.0342
301.1407
78.9855
98.9841
194.1184
4MTB-ITC-Cys standard.d: +MS2(283.0608), 20.0-50.0eV, 5.2min, #3206
C5H11S, [I+H]
103.0579
122.0269
m/z

4MTB ITC-Cyclic-Cys

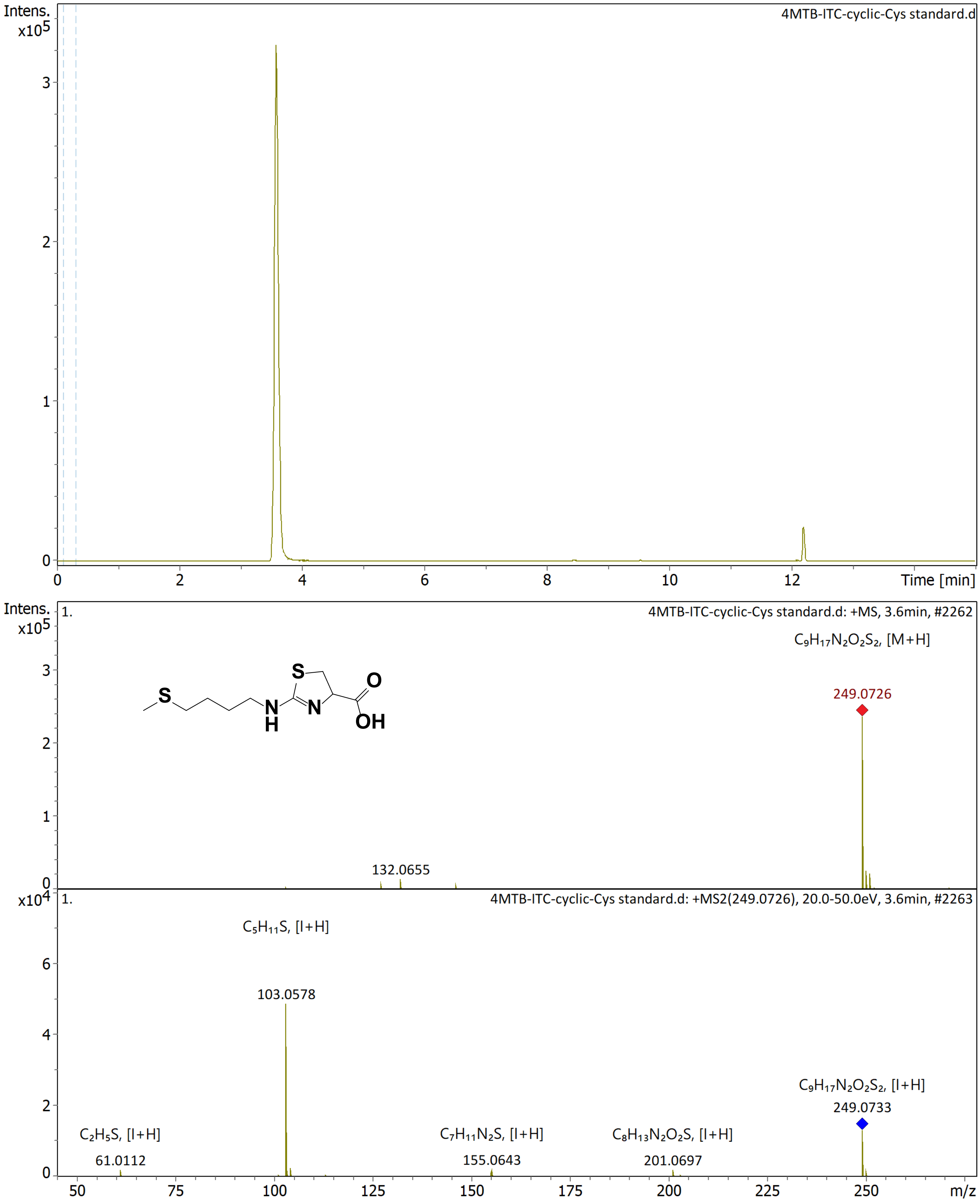

S. littoralis fed on Allyl ITC

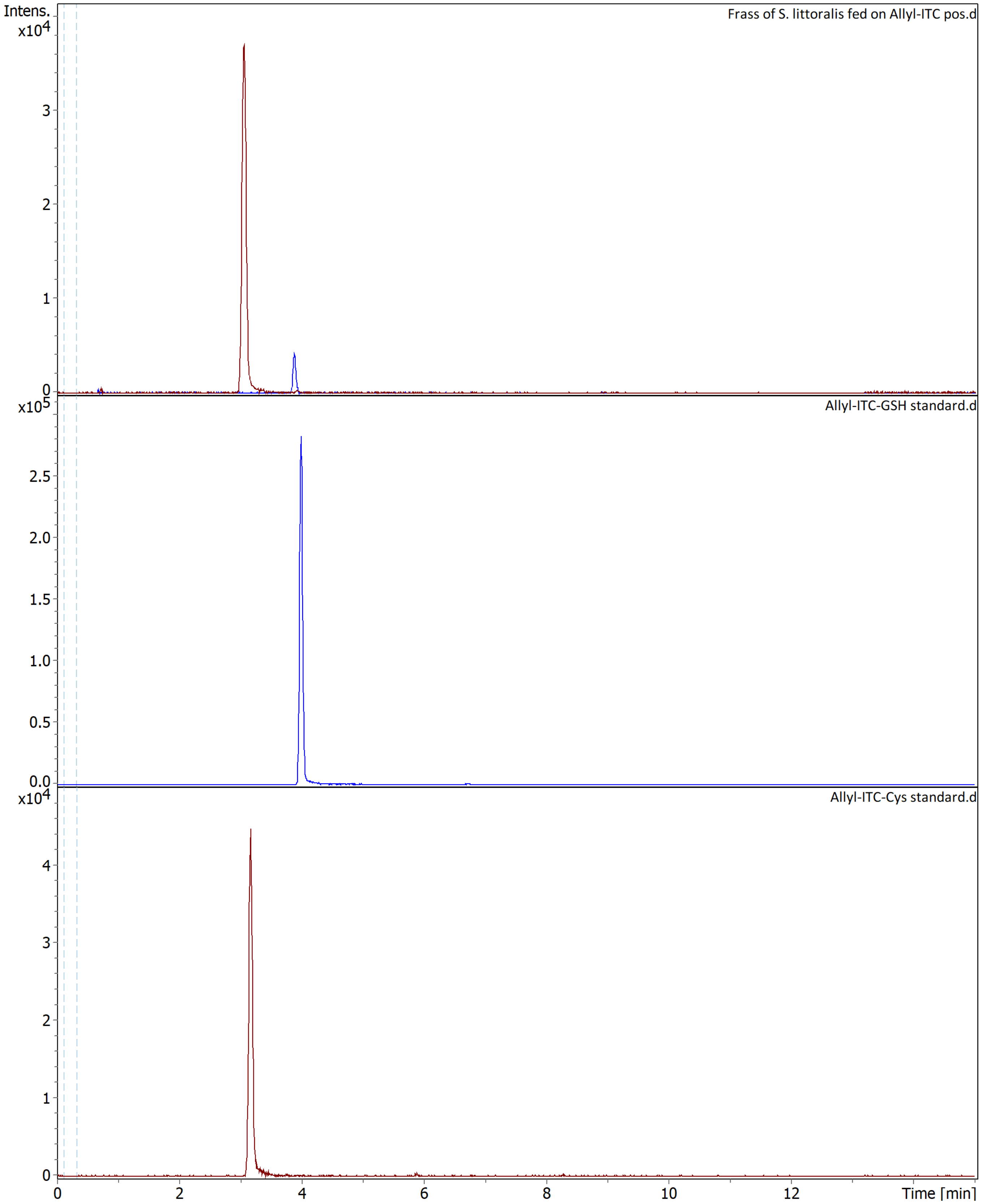

Allyl ITC-GSH

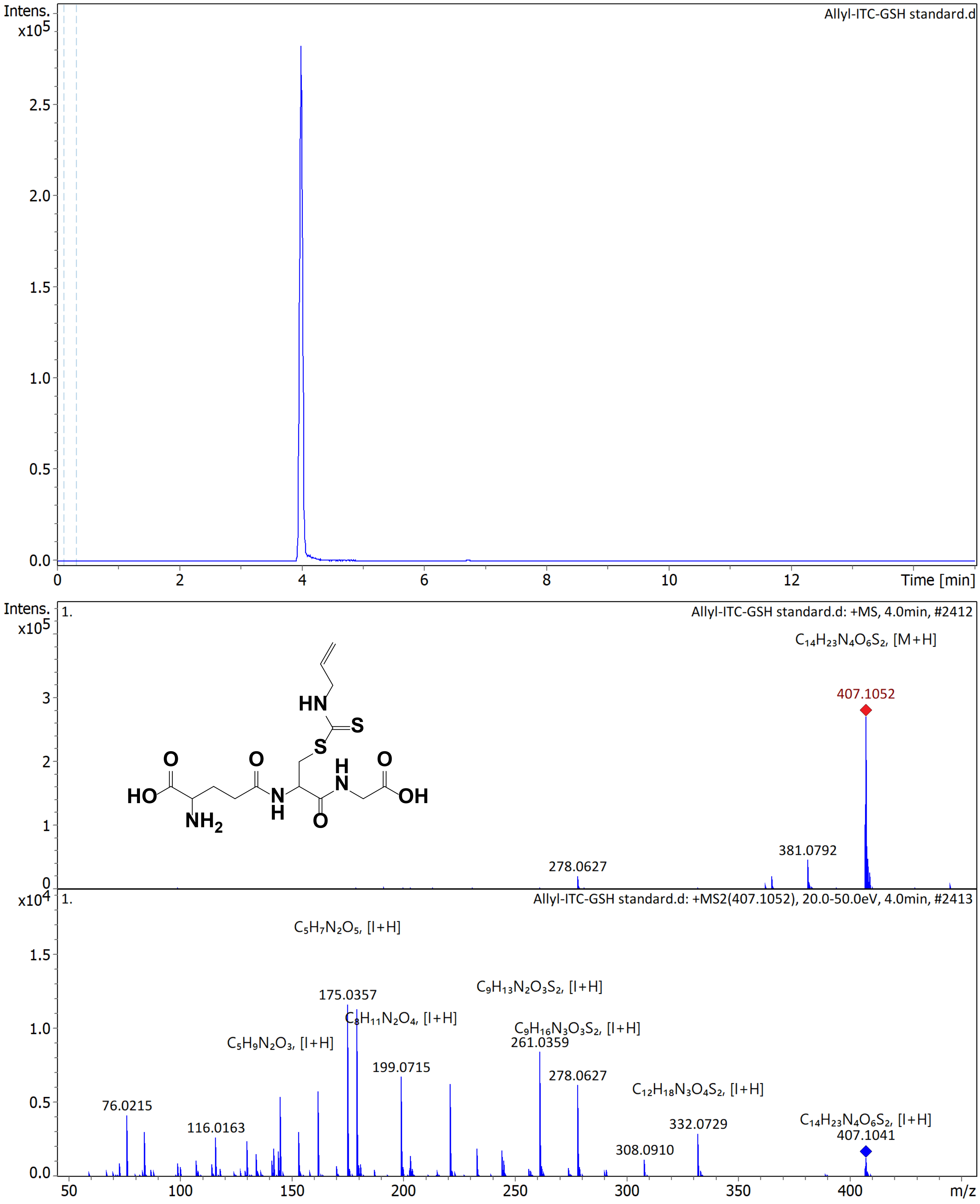

Allyl ITC-Cys

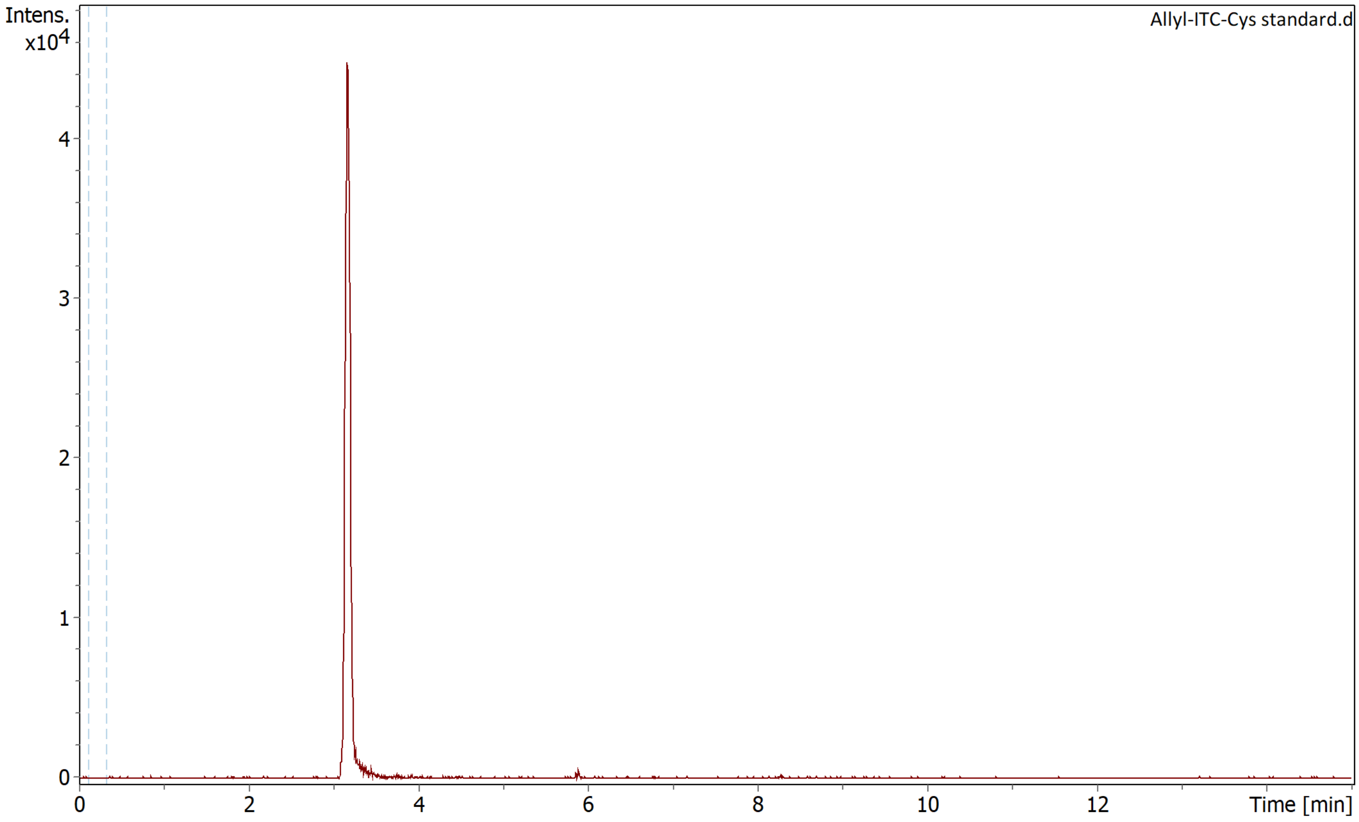

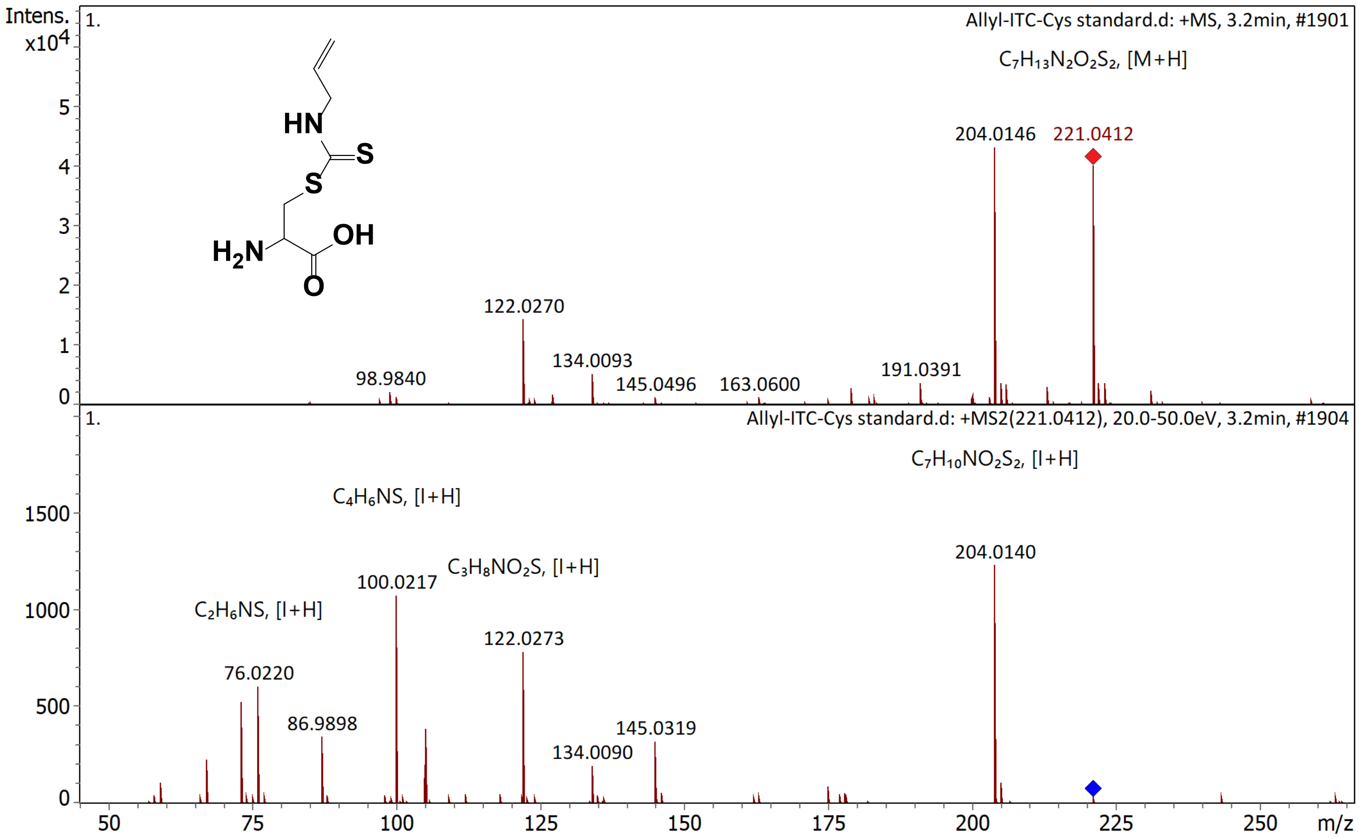

S. littoralis fed on Benzyl ITC

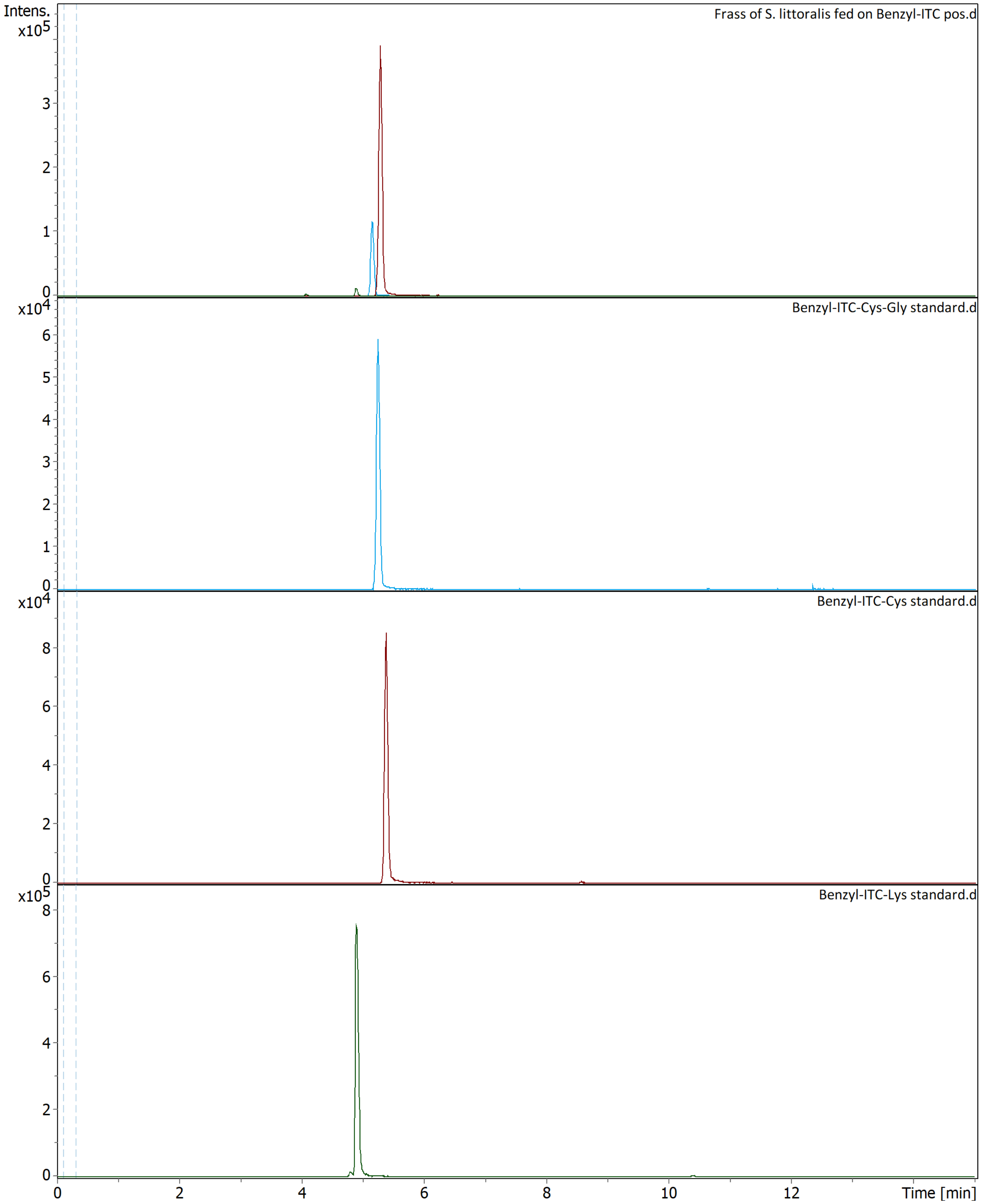

Benzyl ITC-CysGly

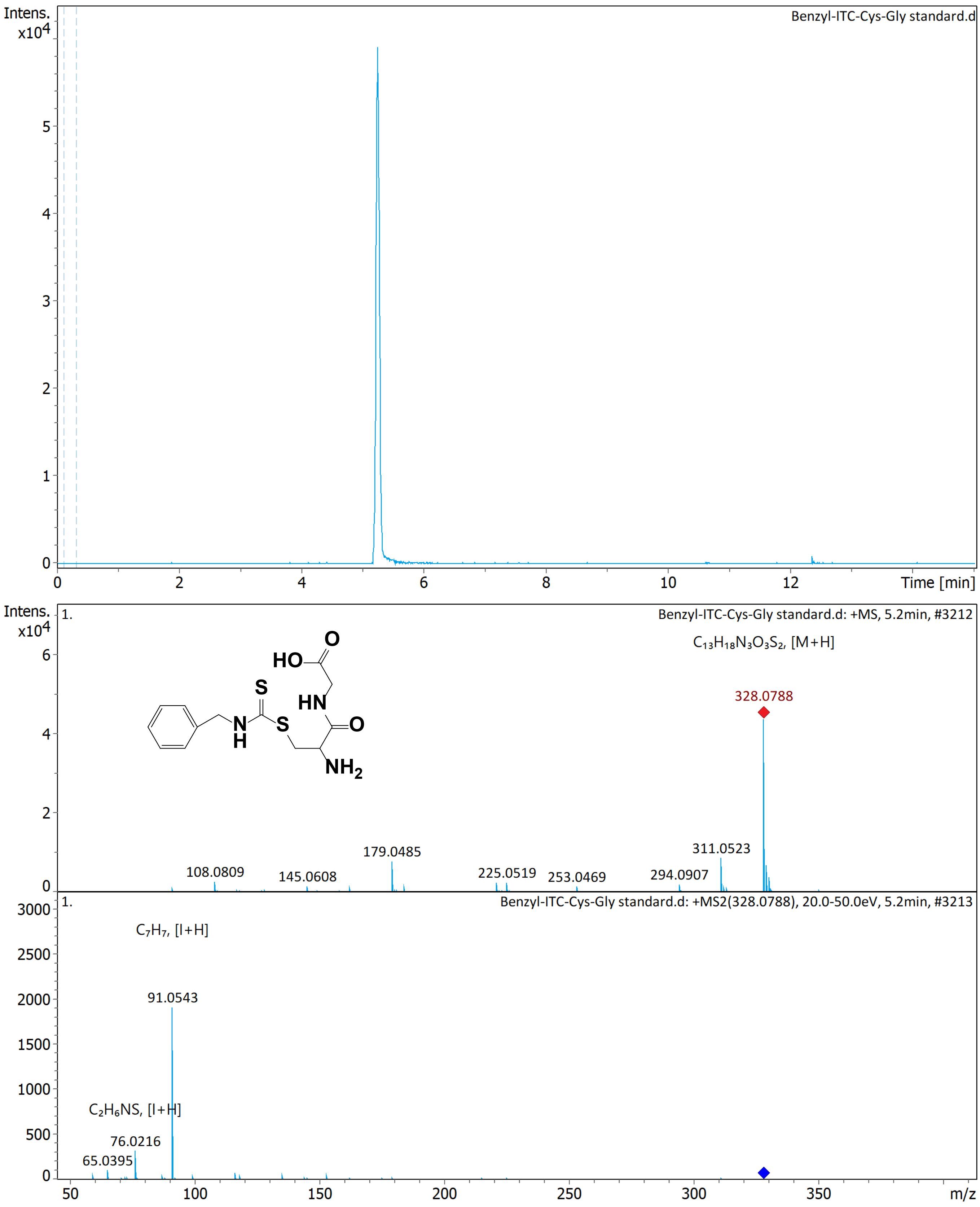

Benzyl ITC-Cys

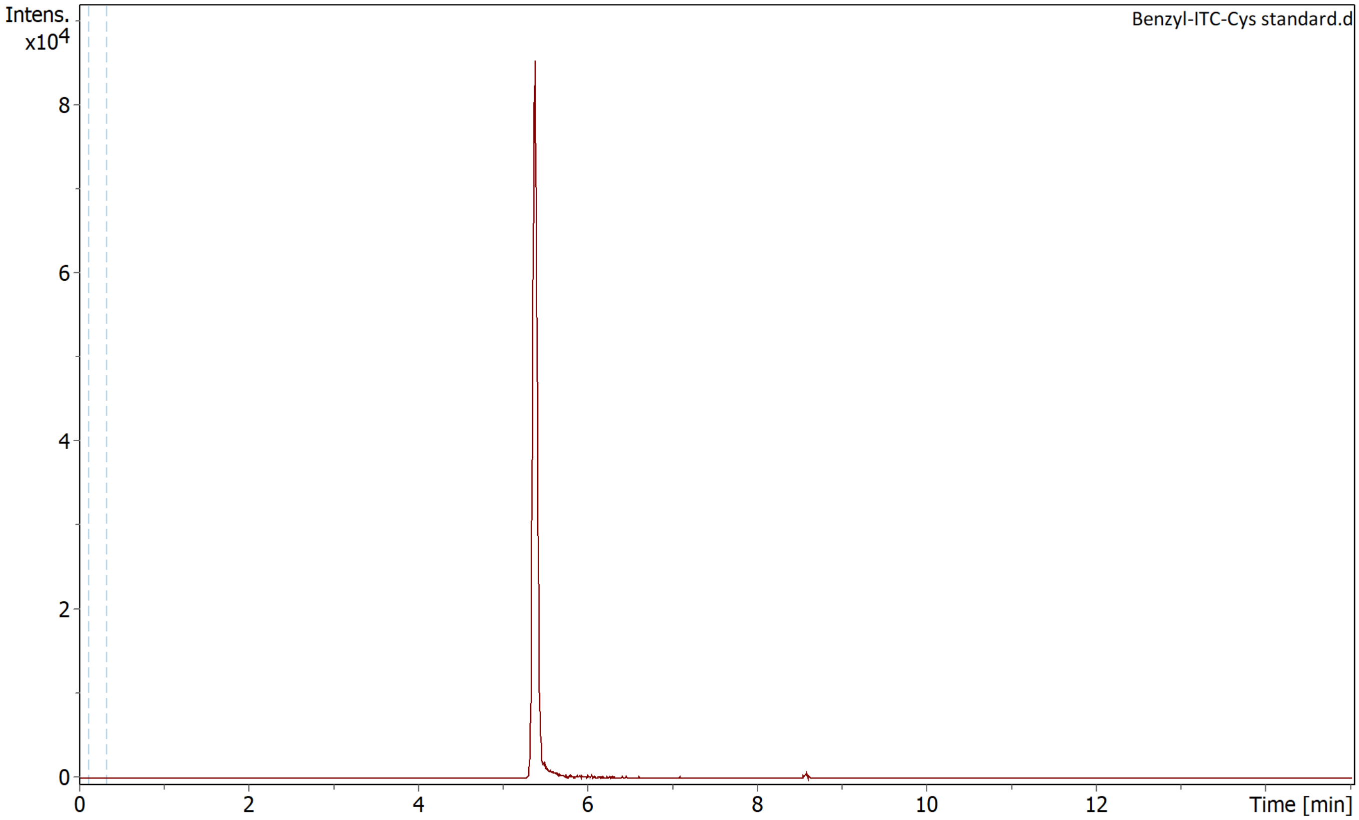

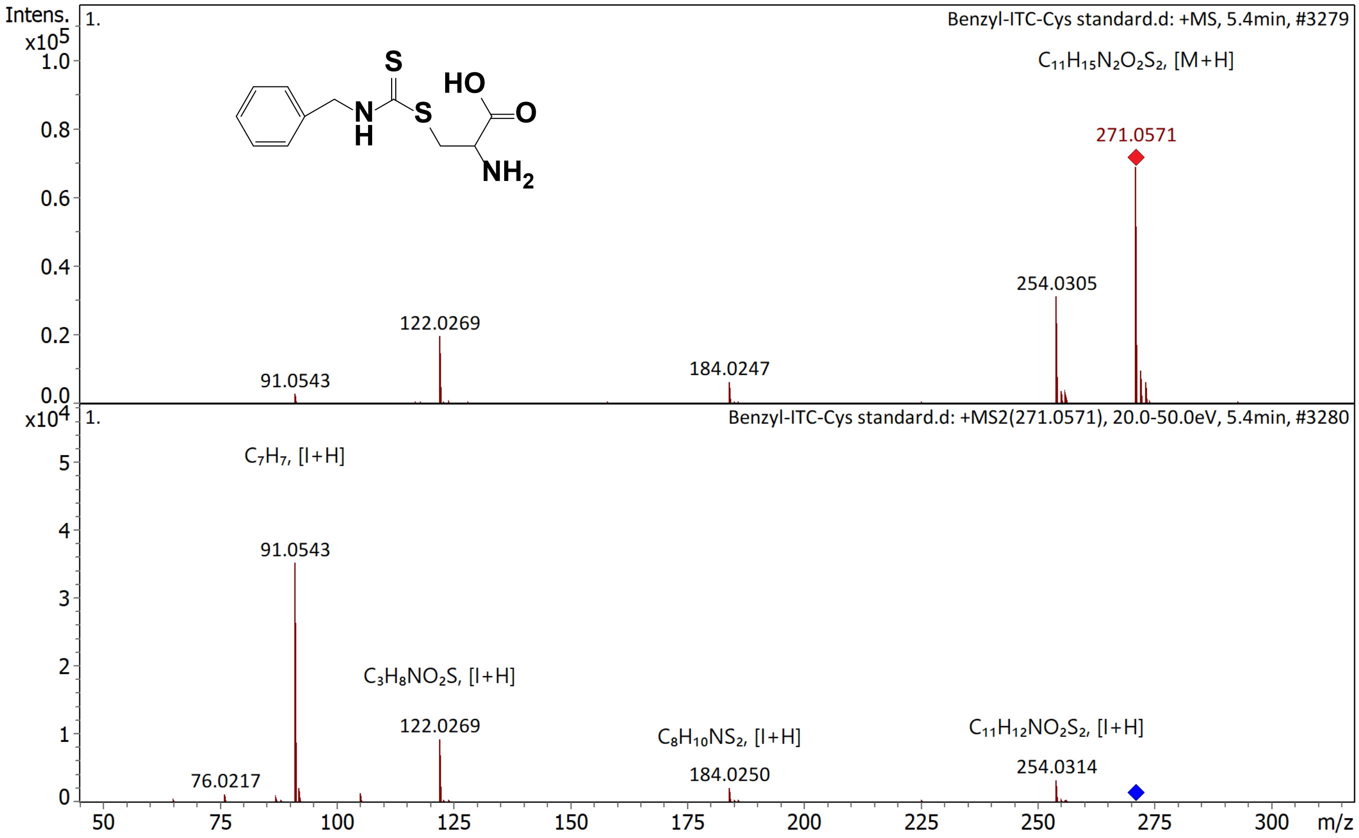

Benzyl ITC-Lys

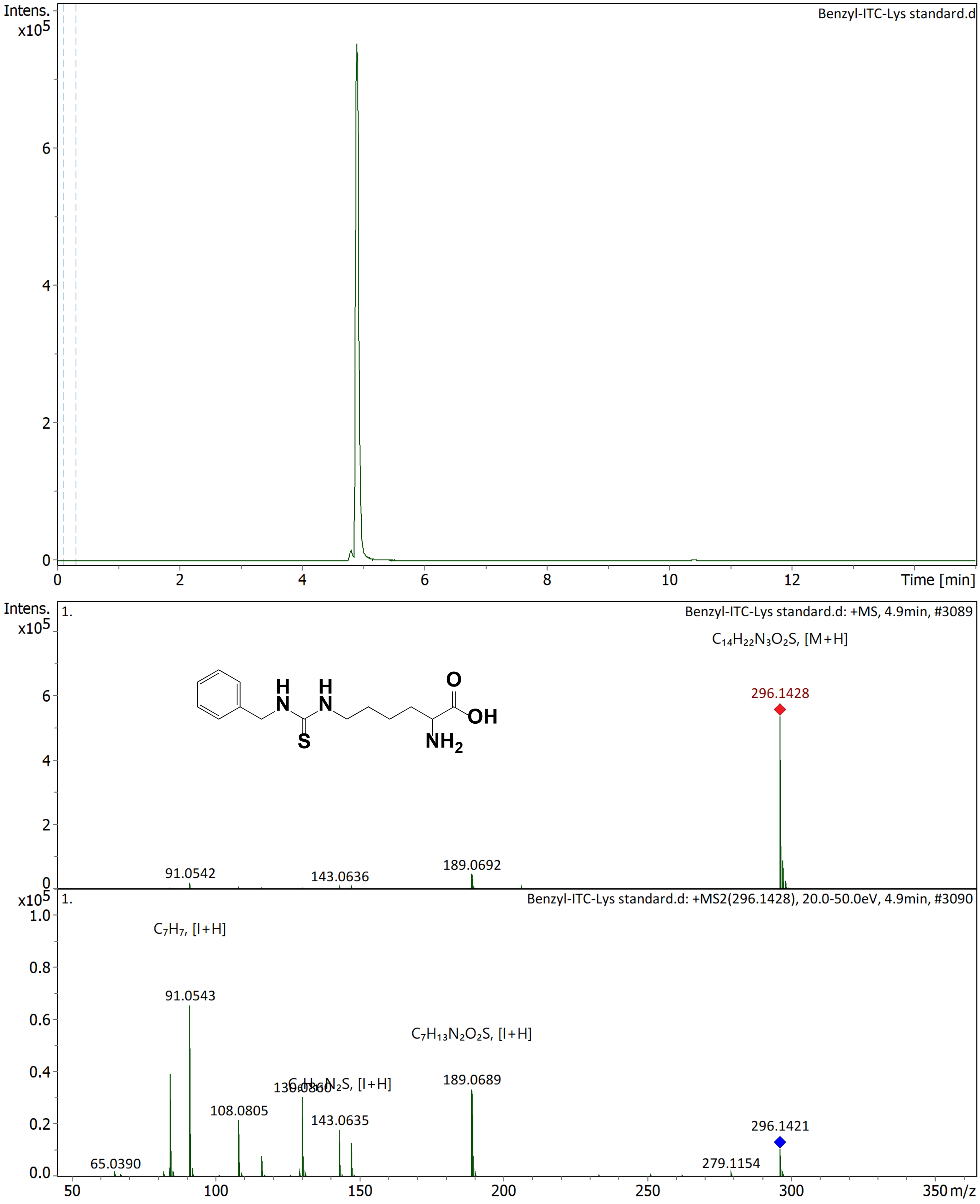

S. littoralis fed on 2PE ITC

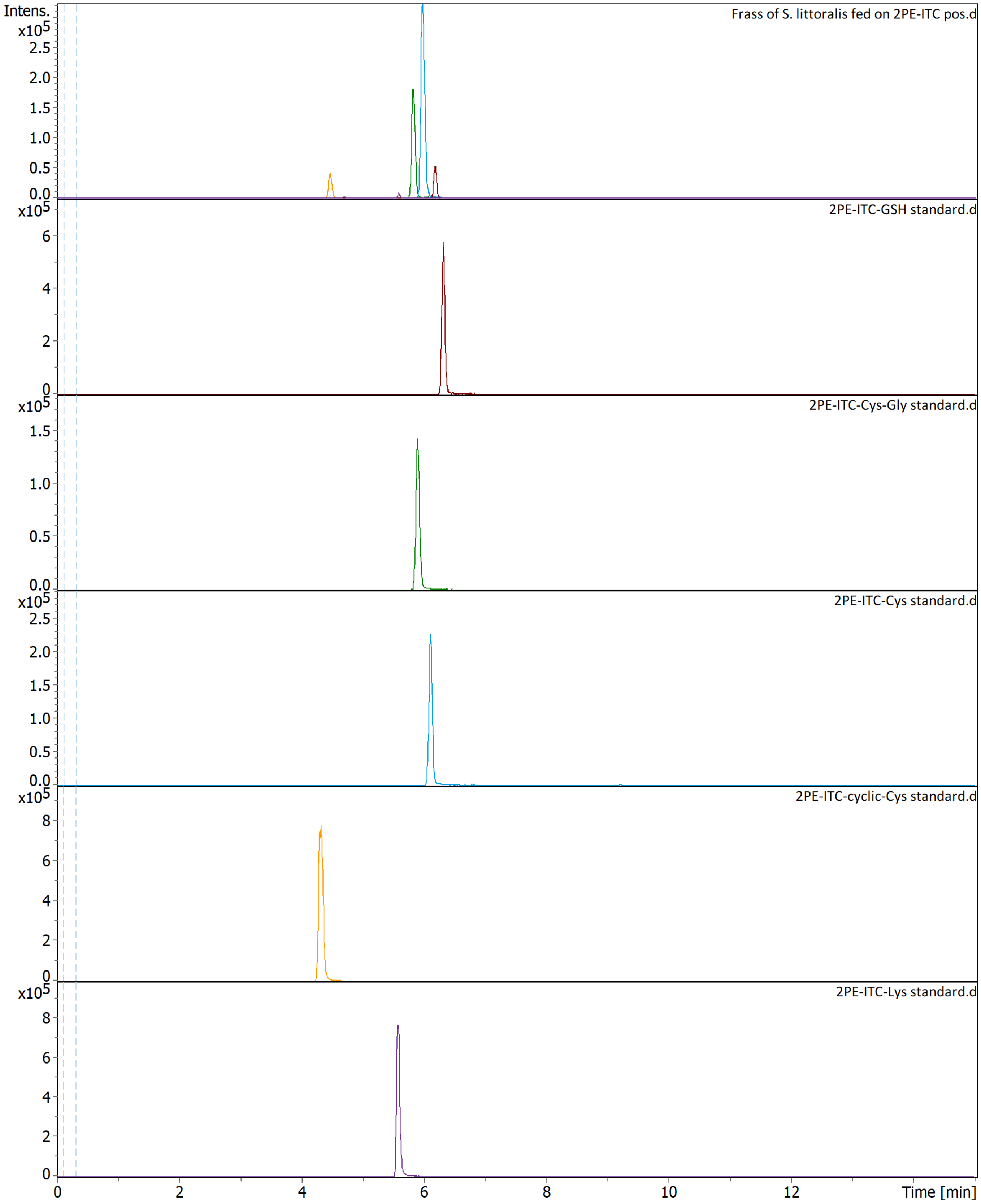

2PE ITC-GSH

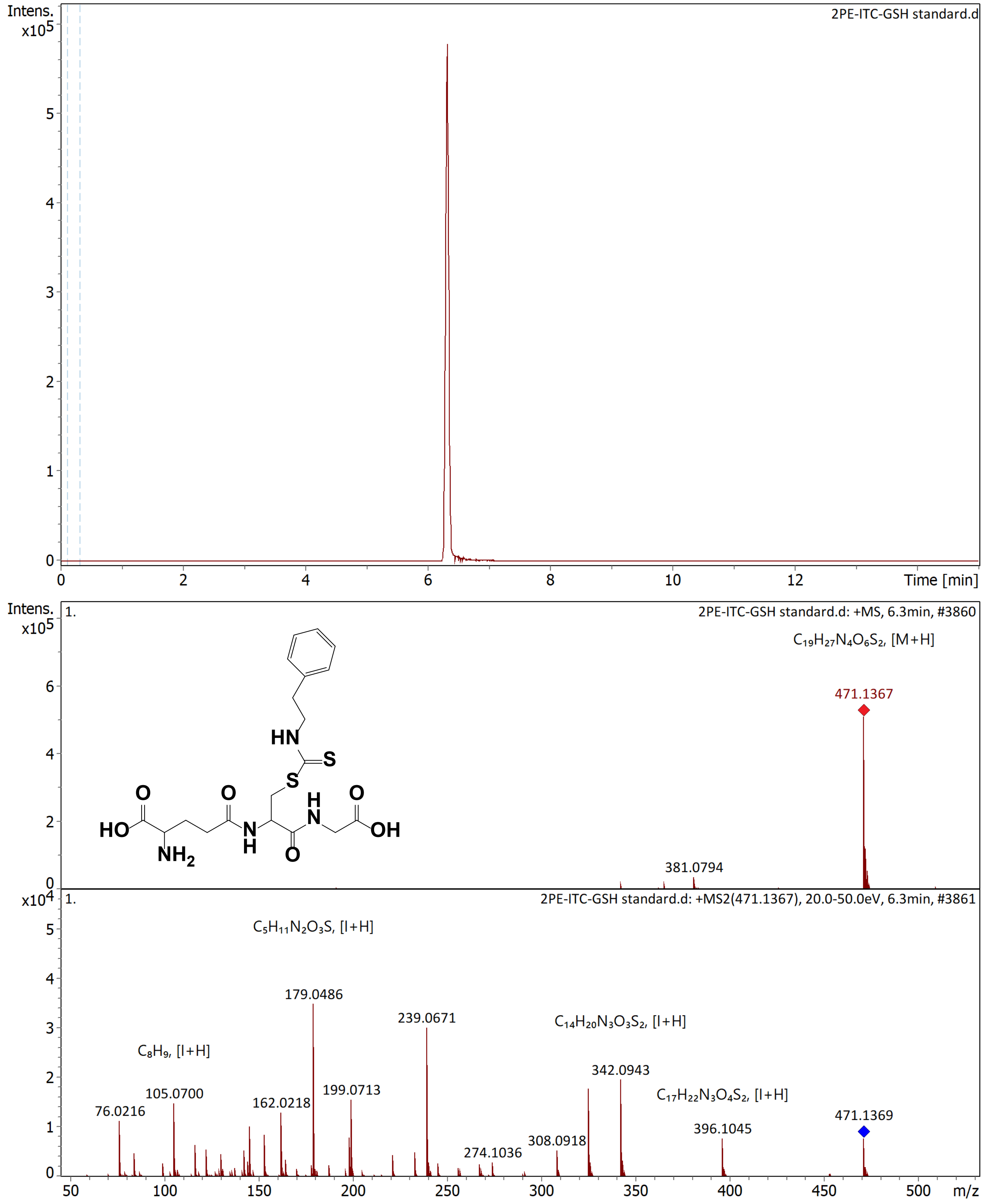
Intens.
x10^5
2PE-ITC-GSH standard.d
Time [min]
Intens.
x10^5
1.
2PE-ITC-GSH standard.d: +MS, 6.3min, #3860
C19H27N4O6S2, [M+H]
471.1367
381.0794
HN
S
S
O
O
H
N
O
HO
N
H
O
OH
NH2
x10^4
1.
2PE-ITC-GSH standard.d: +MS2(471.1367), 20.0-50.0eV, 6.3min, #3861
C5H11N2O3S, [I+H]
179.0486
239.0671
C14H20N3O3S2, [I+H]
C8H9, [I+H]
342.0943
105.0700
199.0713
C17H22N3O4S2, [I+H]
76.0216
162.0218
471.1369
396.1045
308.0918
274.1036
m/z

2PE ITC-CysGly

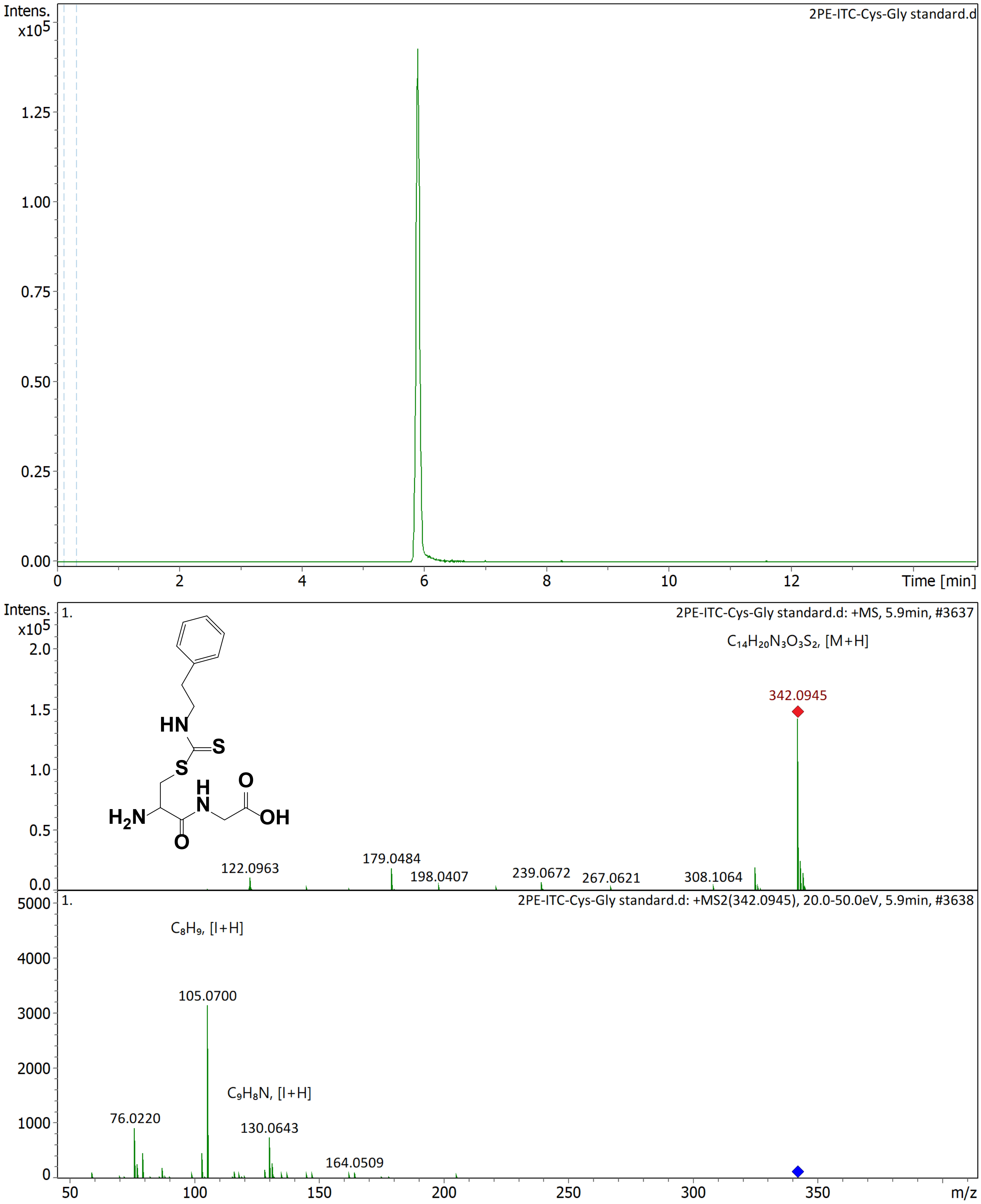

2PE ITC-Cys

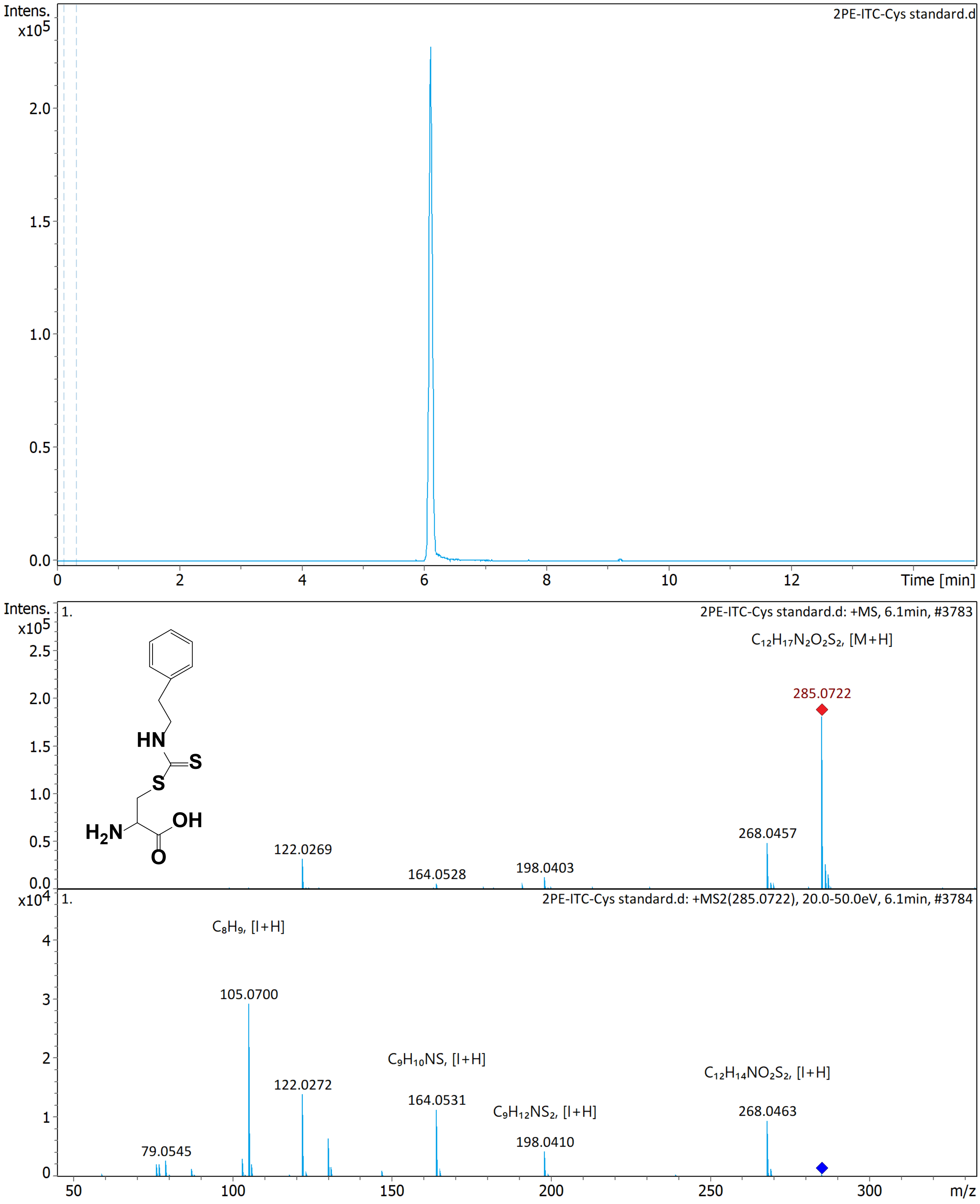

Intens. x10^5
2PE-ITC-Cys standard.d
Time [min]
1.
2PE-ITC-Cys standard.d: +MS, 6.1min, #3783
C12H17N2O2S2, [M+H]
285.0722
268.0457
122.0269
164.0528
198.0403
HN
S
S
H2N
OH
O
x10^4
2PE-ITC-Cys standard.d: +MS2(285.0722), 20.0-50.0eV, 6.1min, #3784
C8H9, [I+H]
105.0700
122.0272
79.0545
C9H10NS, [I+H]
164.0531
C9H12NS2, [I+H]
198.0410
C12H14NO2S2, [I+H]
268.0463
m/z

2PE ITC-Cyclic-Cys

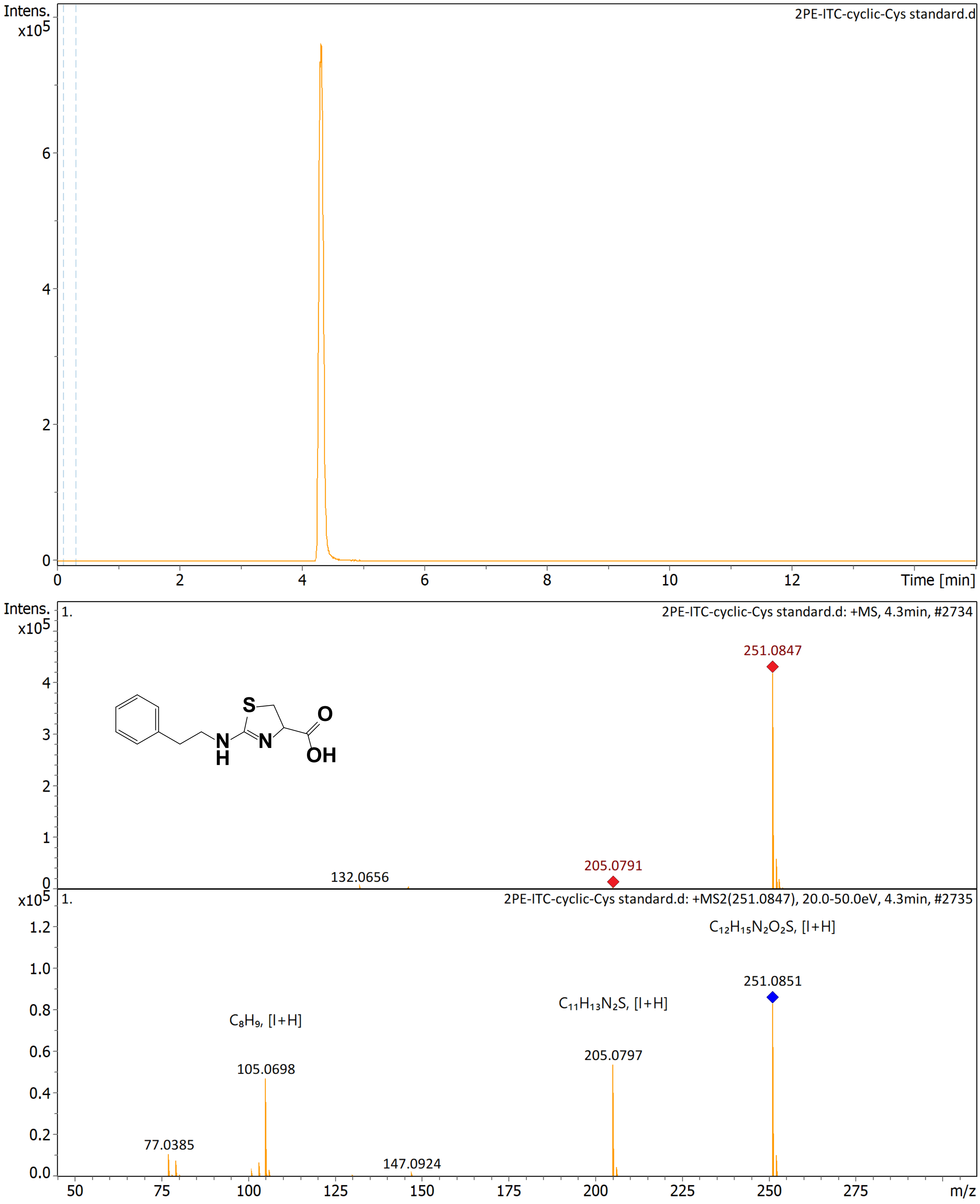

2PE ITC-Lys

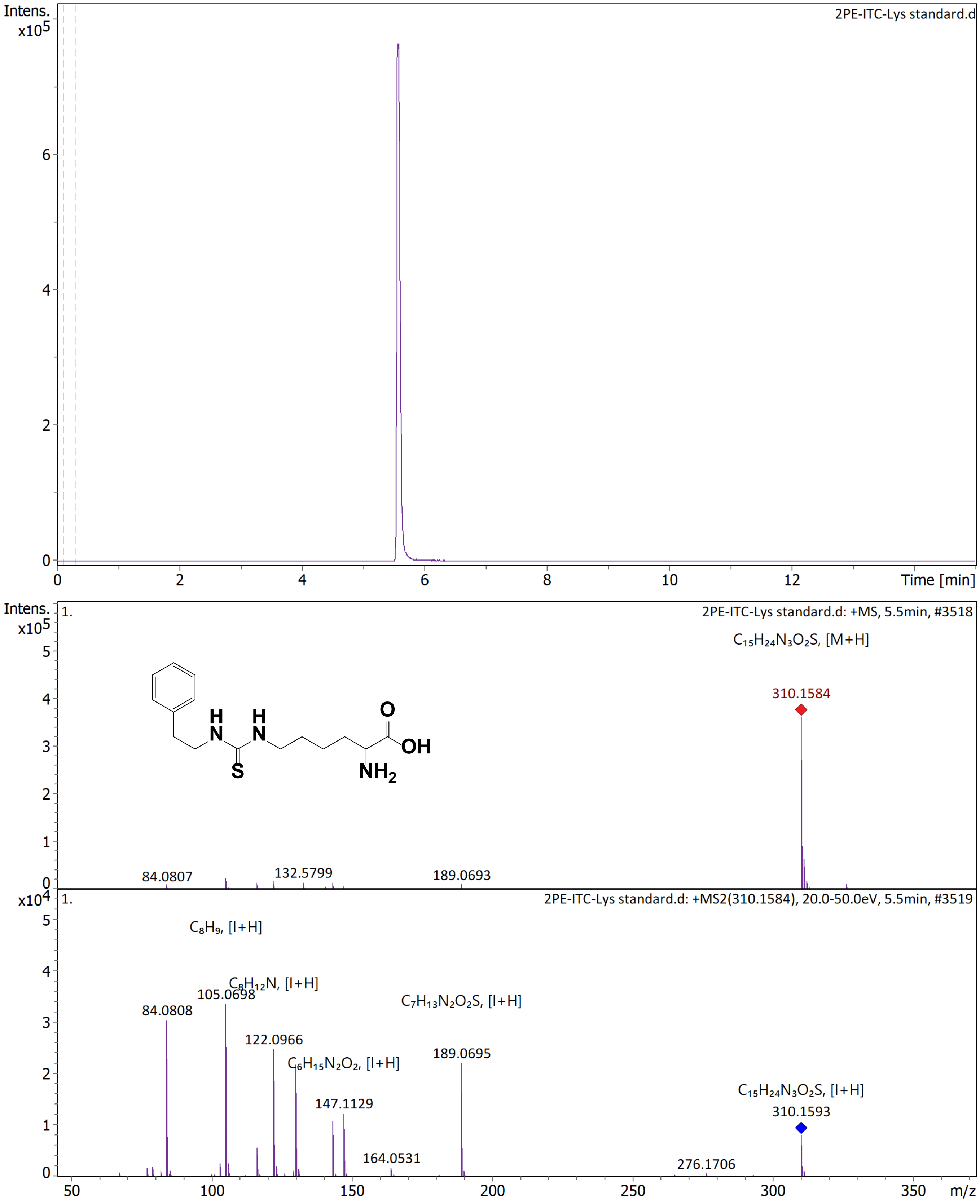
Intens.
x10^5
2PE-ITC-Lys standard.d
Time [min]
1.
2PE-ITC-Lys standard.d: +MS, 5.5min, #3518
C15H24N3O2S, [M+H]
310.1584
84.0807
132.5799
189.0693
x10^4
2PE-ITC-Lys standard.d: +MS2(310.1584), 20.0-50.0eV, 5.5min, #3519
C8H9, [I+H]
C8H12N, [I+H]
105.0698
84.0808
C7H13N2O2S, [I+H]
122.0966
C6H15N2O2, [I+H]
189.0695
147.1129
164.0531
C15H24N3O2S, [I+H]
310.1593
276.1706
m/z

S. littoralis fed on Butyl ITC

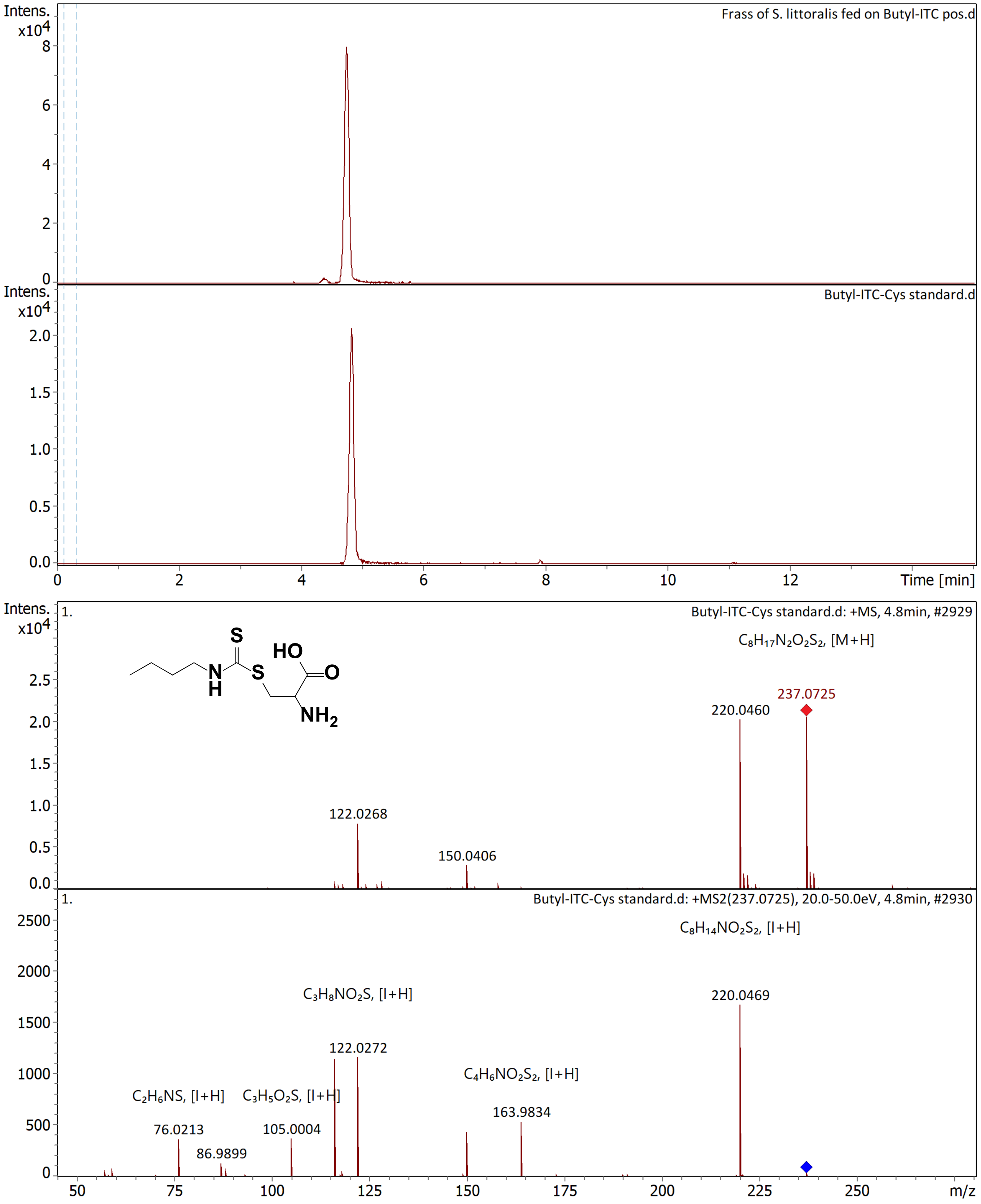

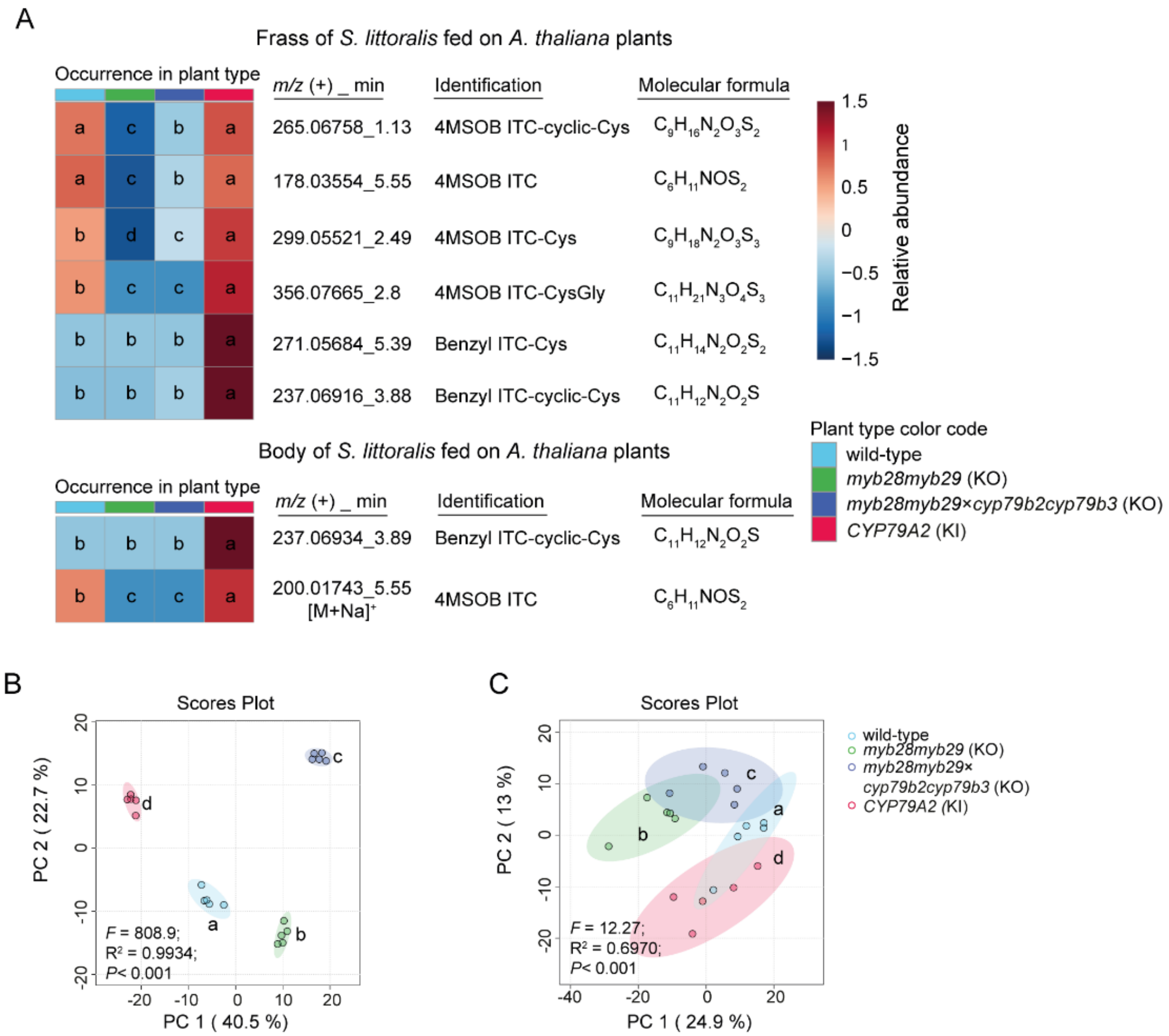

Supplementary Figure 6 The detectable ITCs and ITC conjugates found in *S. littoralis* fed on glucosinolate (GSL)-containing *Arabidopsis thaliana* plants. (A) Non-targeted metabolomics analyses indicate the major metabolites of 4MSOB-GSL and benyzl-GSL in larval frass and bodies of *S. littoralis* fed on *A. thaliana* wild-type plants, *myb28myb29* KO mutants, *myb28myb29×cyp79b2cyp79b3* KO mutants, and *CYP79A2* KI mutants (n = 5). Color key for plant type is given above the table. Data were normalized based on sample weight and $\log_{10}$-transformed. (B-C) Principal component analysis (PCA) plots show the variation among metabolites in larval frass (B) or bodies (C) of *S. littoralis* fed on *A. thaliana* plants. LC-MS/MS features were extracted from non-targeted Q-TOF (UHPLC-HRMS, positive mode) analyses (n = 5). Detailed metabolomics analyses of frass and bodies of *S. littoralis* fed on *A. thaliana* plants are listed in Supplementary Data 3 and Supplementary Data 4. Significant differences between means (±SE) were determined by Fisher's LSD tests in conjunction with one-way ANOVA in A, and by pair-wise PERMANOVA in B and C. Different lowercase letters denote significant differences (P < 0.05).

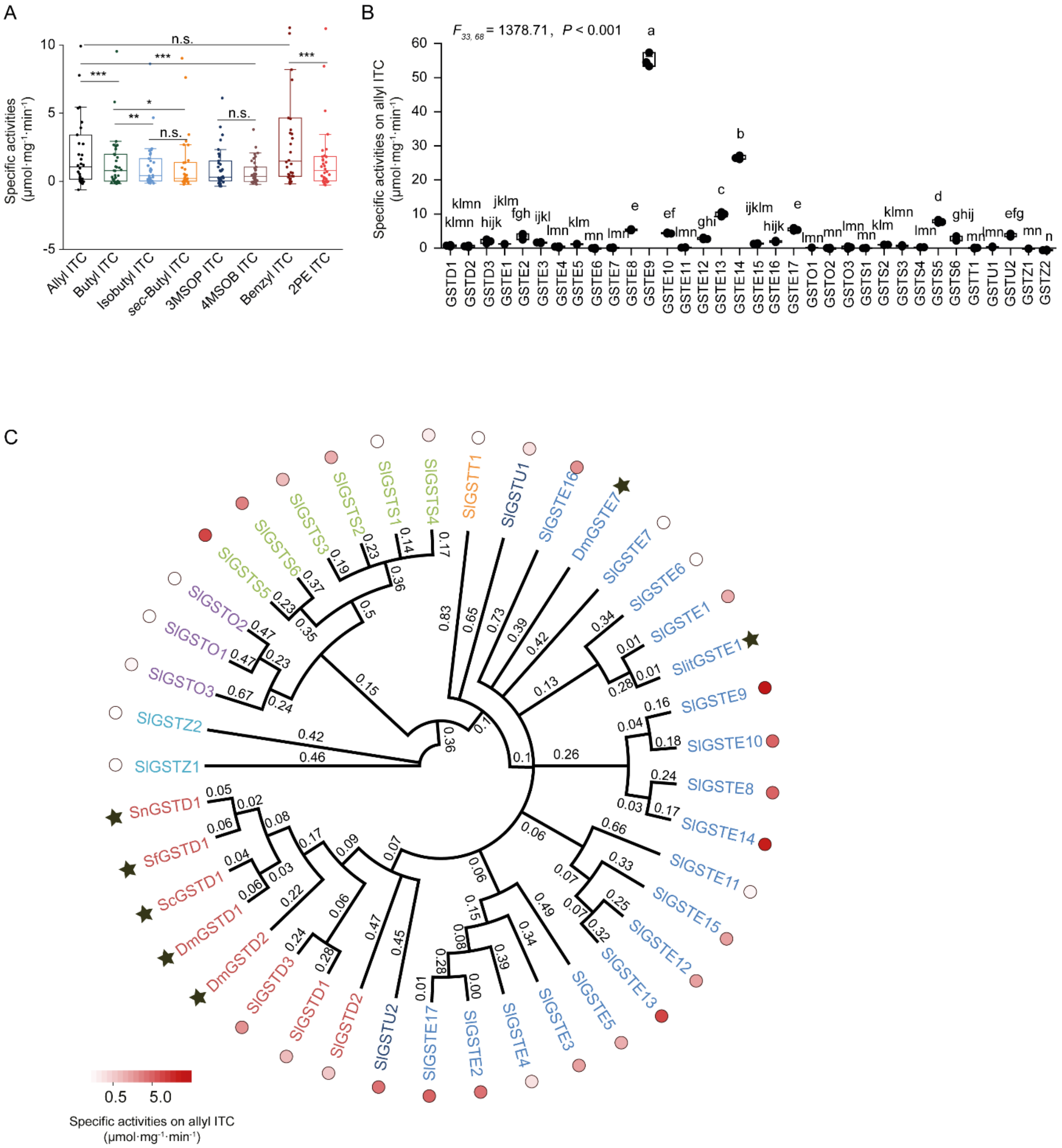

Supplementary Figure 7 Enzymatic activity and phylogenetic analysis of glutathione-*S*-transferases (GSTs) catalyzing the conjugation of GSH with ITCs from this study and the literature. (**A**) *S. littoralis* GST enzyme activities for the conjugation of GSH with various ITCs. The average activity of each GST enzyme was treated as a single data point, and the total number of data points for each ITC was 34. (**B**) Activities of *S. littoralis* His-tagged GSTs in catalyzing the conjugation of allyl ITC with GSH (n =3). (**C**) Phylogenetic tree was built from amino-acid sequences of *S. littoralis* GSTs and previously reported insect

GSTs known to catalyze GSH–ITC conjugation. Circle color indicates the level of *S. littoralis* His-tagged GST activity with allyl ITC; dark stars show the GST proteins from other insects. Species abbreviations: Dm, *Drosophila melanogaster*; Sf, *Scaptomyza flava*; Sn, *Scaptomyza nigrita*; Sc, *Scaptomyza caliginosa*; Slit, *Spodoptera litura*. The GeneBank accession numbers of the protein sequences are indicated in Supplementary Table 5. The analysis was conducted using neighbor joining method in Clustal Omega 1.2.2 program. The scale bar represents amino acid substitutions per site. Significant differences between means (±SE) were determined using a paired-sample Wilcoxon signed-rank test for all pairwise combinations in **A**, with detailed results provided in Supplementary Data 8, and using one-way ANOVA followed by Tukey's HSD test in **B**. In **A**, asterisks indicate statistically significant differences between groups (n.s.: $P \geq 0.05$; *:$P < 0.05$; **: $P < 0.01$; ***: $P < 0.001$). In **B**, different lowercase letters denote statistically significant differences at $P < 0.05$.

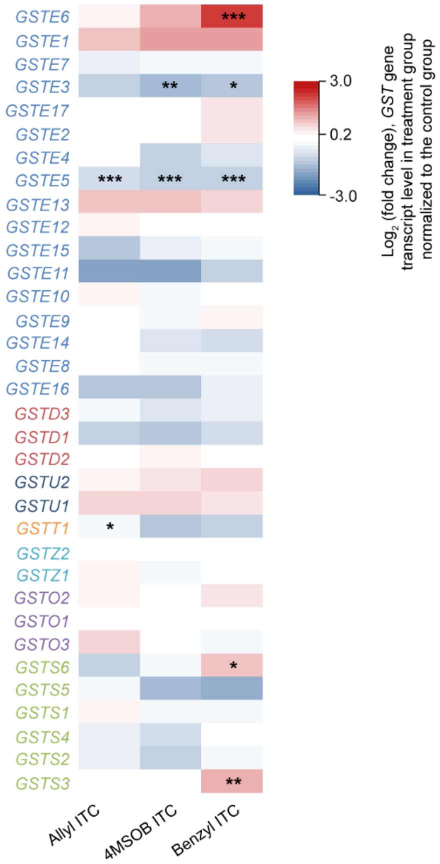

Supplementary Figure 8 Inducibility of *GST* genes in *S. littoralis* larval midgut caused by feeding on artificial diets containing ITCs. The expression level of *GST* genes (relative to *rpl13*) of *S. littoralis* larvae fed on artificial diet containing allyl ITC, benzyl ITC and 4MSOB ITC (1 µmol per g diet) was normalized to the control group larvae fed on artificial diet without ITC. Significant differences between means (±SE) for each gene were determined by Tukey HSD tests in conjunction with one-way ANOVA. The asterisks in the graph show significant differences (*, $p < 0.05$; **, $p < 0.01$; ***, $p < 0.001$) between the ITC-treated group and the control group.

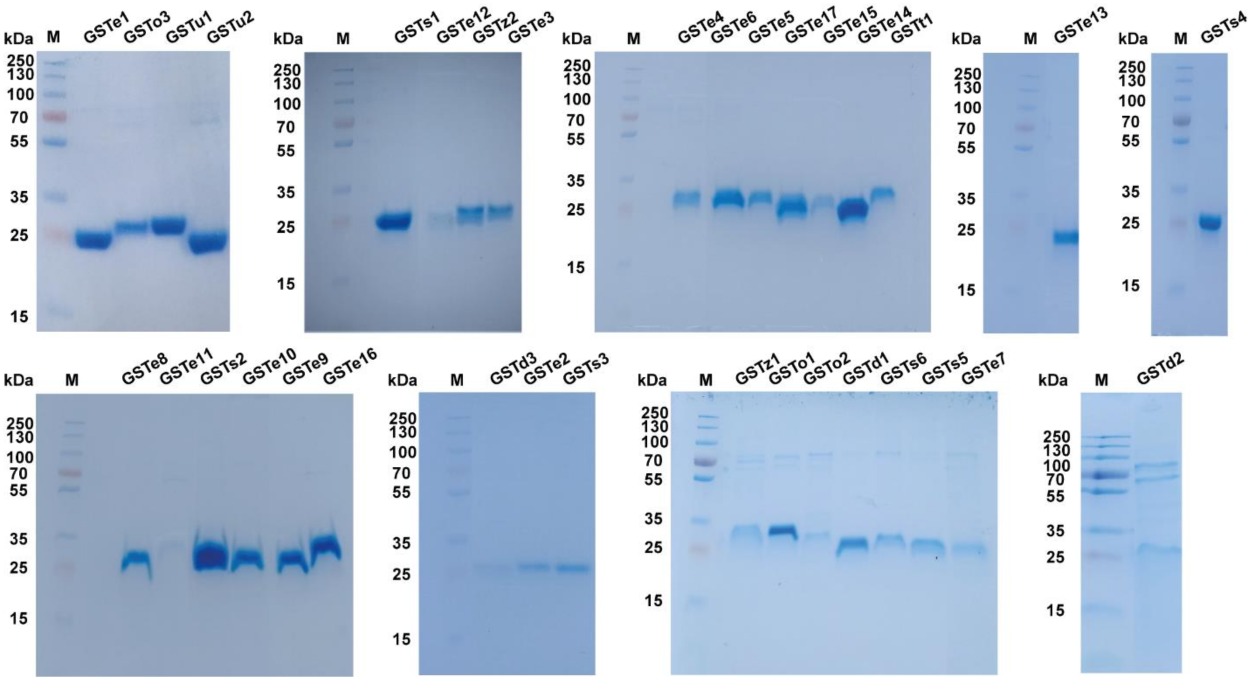

Supplementary Figure 9 SDS-PAGE analysis of purified *S. littoralis* His-tagged GST proteins expressed in the *Escherichia coli* BL21 (DE3) strain. *S. littoralis GST* sequences were independently inserted into the pET28a vector and transformed into *E. coli* to heterologously express recombinant His-tagged proteins. Proteins were purified via His-tag affinity. The molecular weights of the proteins are listed in Supplementary Table 5. The lanes were rearranged to exclude duplicate or non-expressed samples. The marker and protein samples shown in the same image were run on the same electrophoretic gel. The original electrophoretic gel image is available in the Edmond Data Repository.

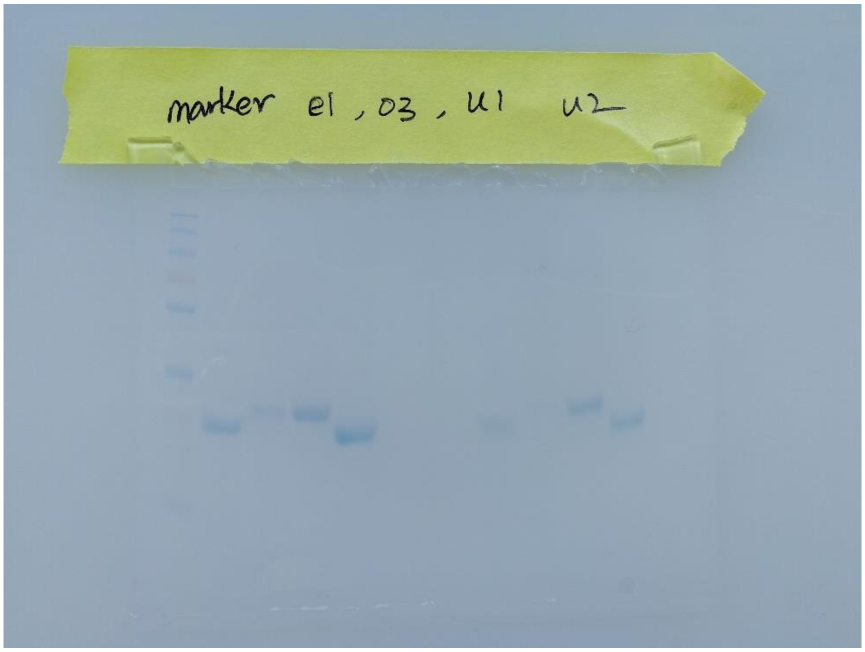

Supplementary Figure 10a The original electrophoretic gel of GSTE1, GSTO3, GSTU1, and GSTU2.

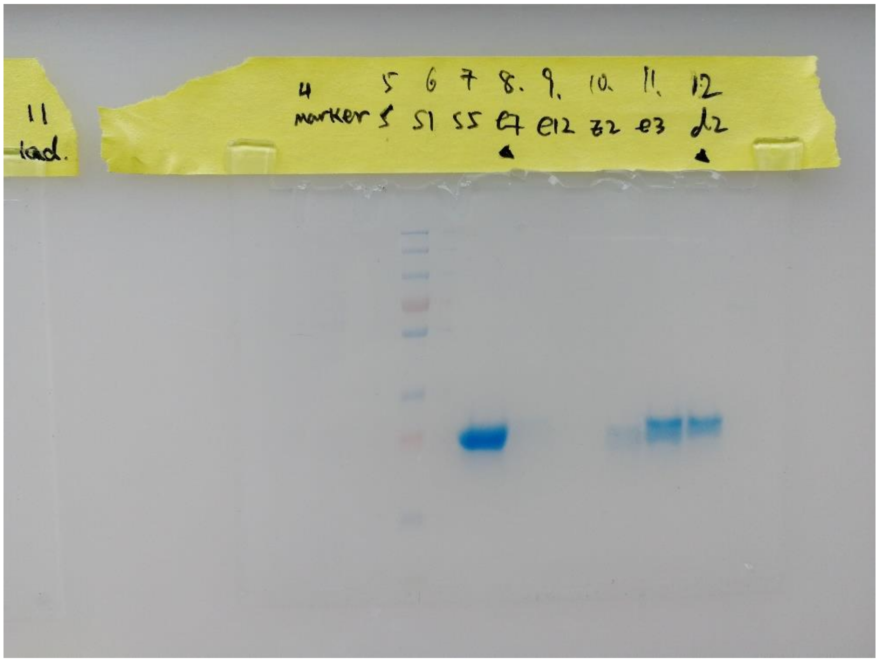

Supplementary Figure 10b The original electrophoretic gel of GSTS1, GSTE12, GSTZ2, and GSTE3.

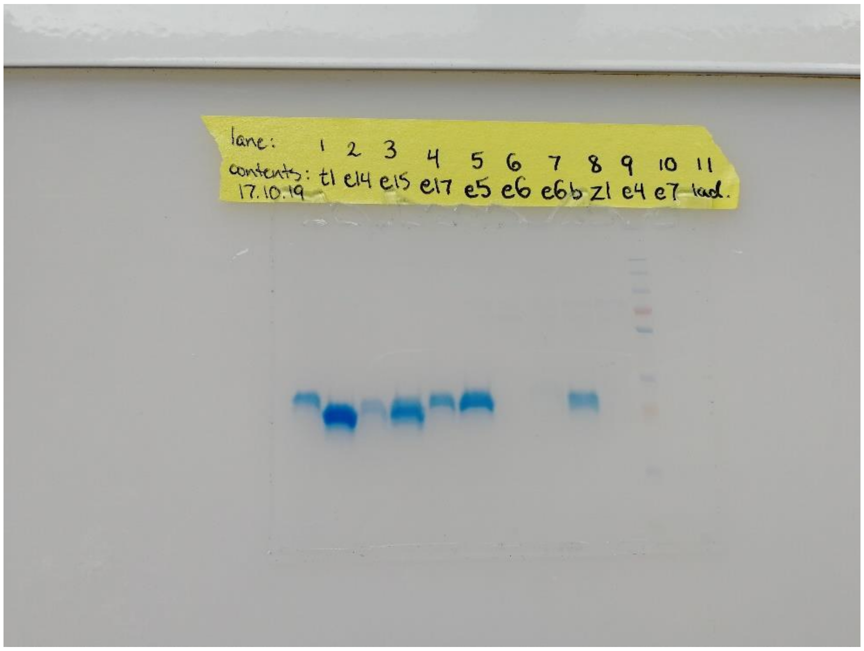

Supplementary Figure 10c The original electrophoretic gel of GSTE4, GSTE6, GSTE5, GSTE17, GSTE15, GSTE14, and GSTT1.

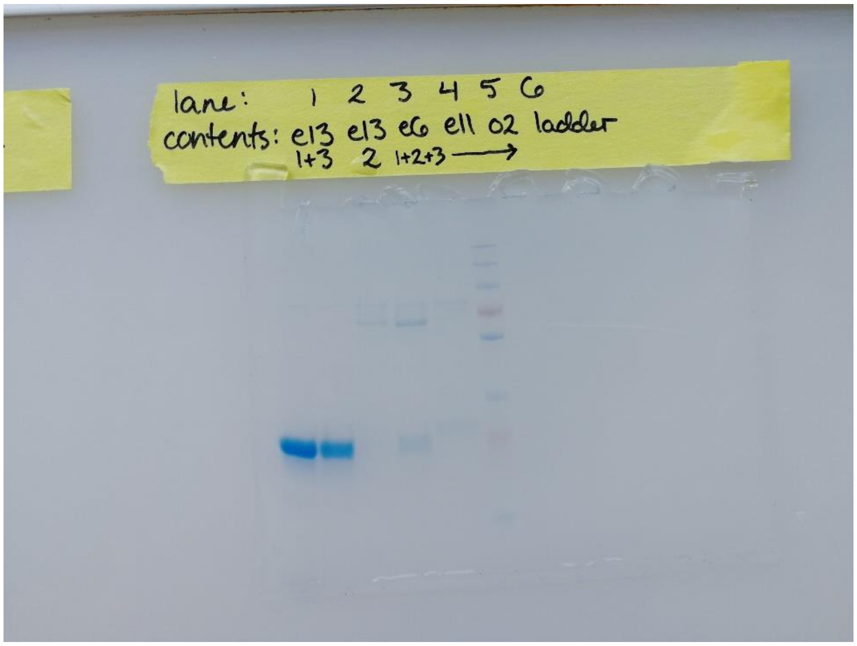

Supplementary Figure 10d The original electrophoretic gel of GSTE13.

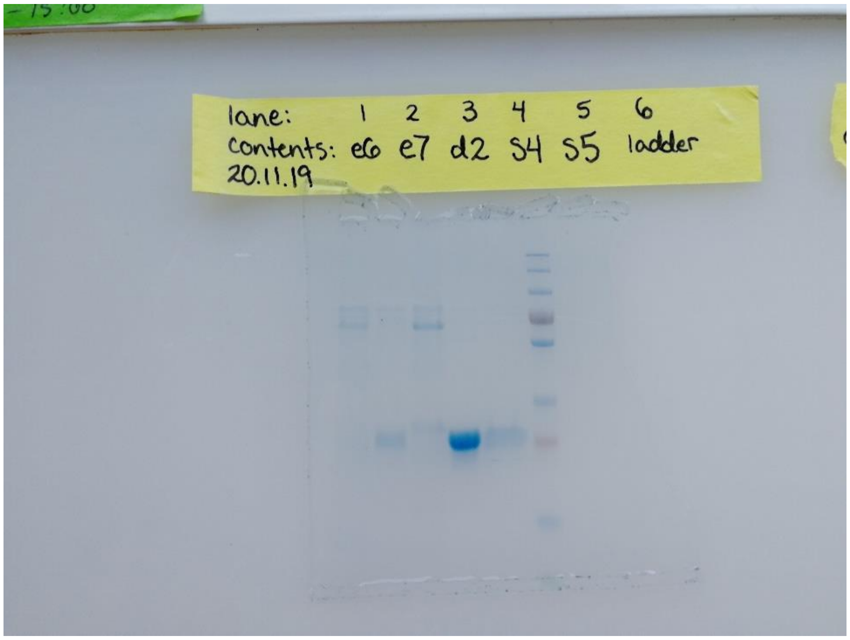

Supplementary Figure 10e The original electrophoretic gel of GSTS4.

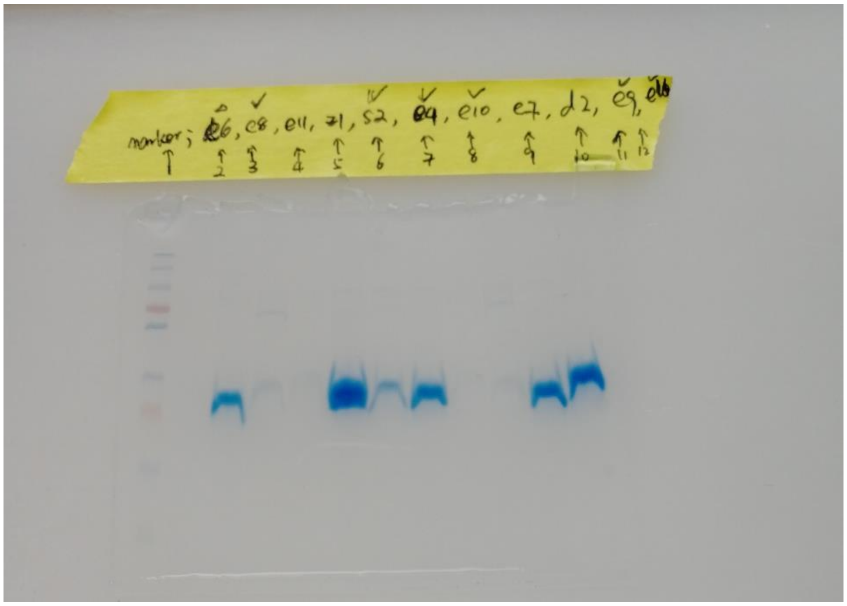

Supplementary Figure 10f The original electrophoretic gel of GSTE8, GSTE11, GSTS2, GSTE10, GSTE9, and GSTE16.

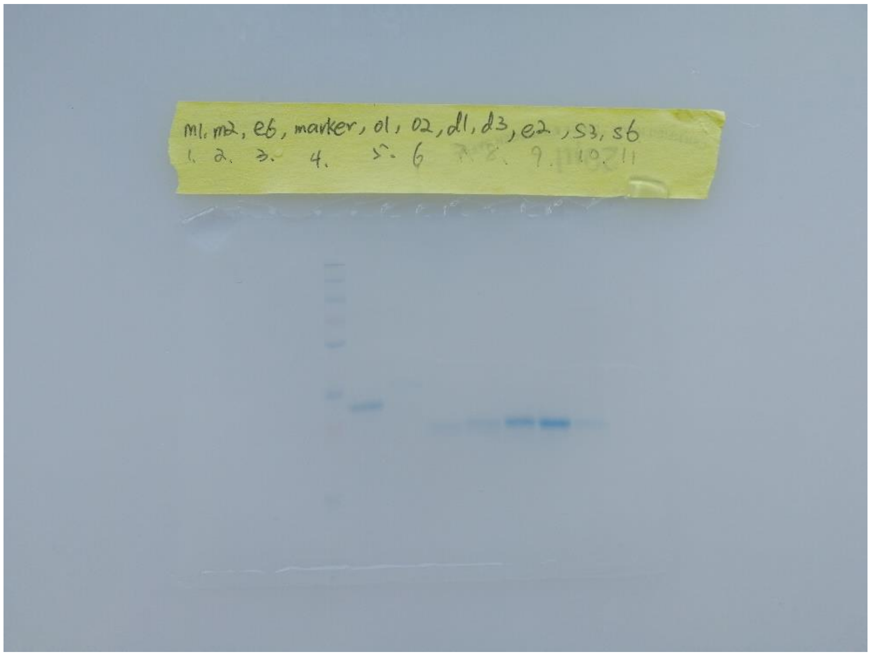

Supplementary Figure 10g The original electrophoretic gel of GSTD3, GSTE2, and GSTS3.

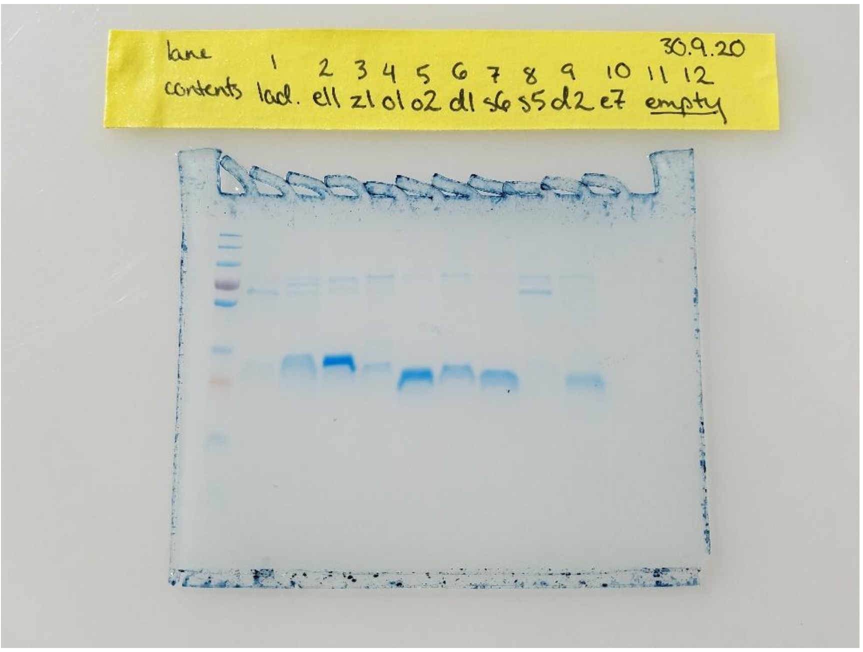

Supplementary Figure 10h The original electrophoretic gel of GSTZ1, GSTO1, GSTO2, GSTD1, GSTS6, GSTS5, and GSTE7.

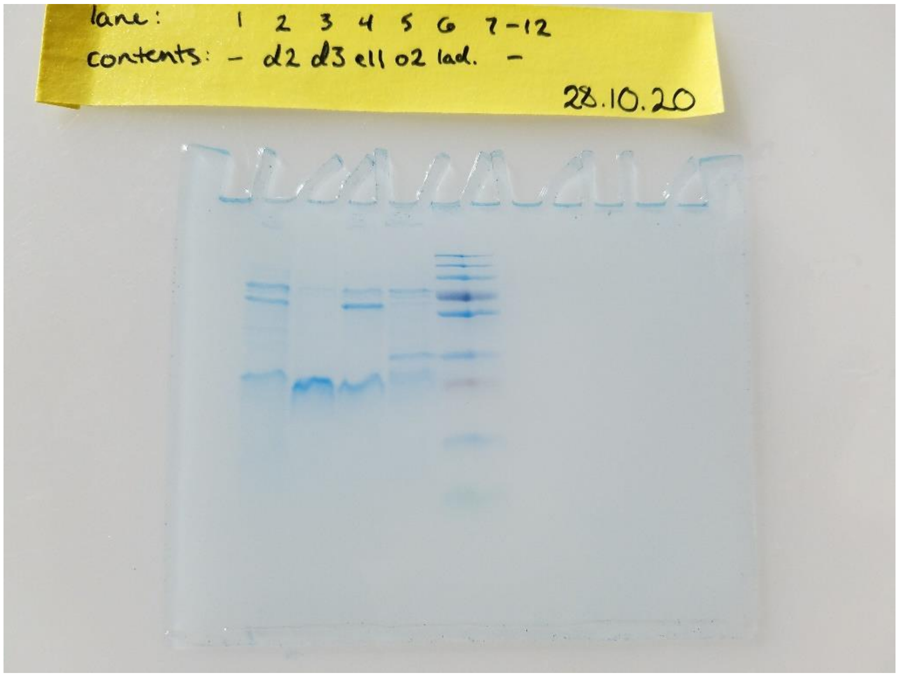

Supplementary Figure 10i The original electrophoretic gel of GSTD2.
