# Supplementary material for: Multiple glutathione-S-transferases detoxify diverse glucosinolate-based defenses of Brassicales plants in a generalist lepidopteran herbivore (Spodoptera littoralis)
Source: Commun Biol. 2025 Jun 17;8:931. doi: 10.1038/s42003-025-08346-8 (PMC12174363; doi:10.1038/s42003-025-08346-8)
Supplement: Supplementary file 3 — Description of Additional Supplementary Files [file 42003_2025_8346_MOESM3_ESM.pdf]

Description of Additional Supplementary Files

File name: Supplementary Data 1

Description: Metabolomics analyses of the frass of *S. littoralis* fed on artificial diets containing ITCs.

File name: Supplementary Data 2

Description: Metabolomics analyses of the bodies of *S. littoralis* fed on artificial diets containing ITCs.

File name: Supplementary Data 3

Description: Metabolomics analyses of the frass of *S. littoralis* fed on *A. thaliana* wild-type and mutant plants.

File name: Supplementary Data 4

Description: Metabolomics analyses of the bodies of *S. littoralis* fed on *A. thaliana* wild-type and mutant plants.

File name: Supplementary Data 5

Description: Primers used for gene cloning and qPCR.

File name: Supplementary Data 6

Description: The significantly different metabolites formed by *S. littoralis* incubated with ITCs compared with control treatment.

File name: Supplementary Data 7

Description: Identity % of *S. littoralis* GSTs based on amino acid sequences.

File name: Supplementary Data 8

Description: The source data behind all graphs and tables in the paper
